# Supplementary material for: Establishing and using a genetic database for resolving identification of fish species in the Sea of Galilee, Israel
Source: PLoS One. 2022 May 19;17(5):e0267021. doi: 10.1371/journal.pone.0267021 (PMC9119452; doi:10.1371/journal.pone.0267021)
Supplement: Attachment — Submitted filename: PONE-D-20-39138_R1_reviewer.pdf [file pone.0267021.s005.pdf]

PLOS ONE

Establishing and using a genetic database for resolving identification of fish species in the Sea of Galilee, Israel

--Manuscript Draft--

**Manuscript Number:**	PONE-D-20-39138R1
**Article Type:**	Research Article
**Full Title:**	Establishing and using a genetic database for resolving identification of fish species in the Sea of Galilee, Israel
**Short Title:**	DNA barcoding the fish species of the Sea of Galilee
**Corresponding Author:**	Lior David<br>The Hebrew University of Jerusalem<br>Rehovot, ISRAEL
**Keywords:**	Lake Kinneret; DNA Barcoding; Fish species identification; Molecular Taxonomy; freshwater fish; species conservation; Jordan valley
**Abstract:**	Freshwaters are a very valuable resource in arid areas, such as Mediterranean countries. Freshwater systems are vulnerable ecological habitats, significantly disturbed globally and especially in arid areas. The Sea of Galilee is the largest surface freshwater body in the Middle East. It is an isolated habitat supporting unique fish populations, including endemic species and populations on the edge of their distribution range. Using the Sea of Galilee for water supply, fishing and recreation has been placing pressure on these fish populations. Therefore, efficient monitoring and effective actions can make a difference in the conservation of these unique fish populations. To set a baseline and develop molecular tools to do so, in this study, DNA barcoding was used to establish a database of molecular species identification based on sequences of Cytochrome C Oxidase subunit I gene. DNA barcodes for 22 species were obtained and deposited in Barcode of Life Database. Among these, 12 barcodes for 10 species were new to the database and different from those already there. Barcode sequences were queried against the database and similar barcodes from the same and closely related species were obtained. Disagreements between morphological and molecular species identification were identified for five species, which were further studied by phylogenetic and genetic distances analyses. These analyses suggested the Sea of Galilee contained hybrid fish of some species and other species for which the species definition should be reconsidered. Notably, the cyprinid fish defined as Garra rufa , should probably be considered as Garra jordanica . Taken together, along with data supporting reconsideration of species definition, this study sets the basis for further using molecular tools for monitoring fish populations, understanding their ecology, and effectively managing their conservation in this unique and important habitat and in the region.
**Order of Authors:**	Roni Tadmor-Levi
	Tomer Borovski
	Evgeniya Marcos-Hadad
	James Shapiro
	Gideon Hulata
	Daniel Golani
	Lior David
**Response to Reviewers:**	The authors would like to thank the editor and reviewers for their constructive detailed comments. We followed these comments one by one revising the text accordingly. It is evident from this letter of response and also from the very busy 'track changes' version. Following these useful suggestions, we now feel that the revised version is a significant improvement over the initial submission to the level ready for publication. Bellow is a point-by-point response to the specific comments (Also provided as a separate file).<br><br>Response to editor’s points:

Powered by Editorial Manager® and ProduXion Manager® from Aries Systems Corporation

1. We went over the manuscript and accompanied files to ensure they meet PLOS ONE style requirements. The revised files are submitted.
2. We would like our manuscript to be considered for the Freshwater Ecosystems collection.
3. We publicly released the repository data as stated in the Data Availability section.
4. Figure 2 was edited and provided at higher resolution. Additionally, we added a copyright statement in figure 2 legend.

Reviewer #1
Comment: This paper describes very well the DNA barcoding of the freshwater fish found in Israel (mainly the Sea of Galileea). All required analyses were well done and I only found two digitation mistakes in species names: line 304 ghorensis, and line 353: Garra (italic). I also do not think that the authors should use names as Garra spp. because this ssp. means species of this genus and when we cite the genus we are already talking about its species.
Response: We appreciate the recommendation. The two digitations mistakes were fixed and spp. was deleted from the text.

Reviewer #2:
Abstract
The authors summarize the main research questions and key findings very well. Using the abstract as a foundation, the introduction and discussion should highlight these points in order to strengthen them.
Response: Accordingly, we almost did not change the abstract, except for a few sentences to emphasizing the points recommended by the reviewer in the discussion section.

Introduction
DNA barcoding was used to establish a database of molecular species identification based on sequences of CO1 gene for the Sea of Galilee, Israel. Applied DNA barcoding to samples taken mainly from the Sea of Galilee to address taxonomic questions and build a reference barcoding database for molecular identification of fish in the context of the Sea of Galilee and the region.
Comment: The study should more explicitly indicate that it is not building a new database but rather adding to an existing database such as BOLD. More information of the BOLD database should be included in the introduction as well as the standardised rules that apply to DNA barcoding. The study fails to relate to previous research in this area. The authors should include more examples, information and scenarios as well as reference the related literature, especially by including more recently published work. Hence, a stronger link between the literature and the topic of this manuscript is required. In the materials and methods section, you mention that methods were adopted from Borovski et al. (2018), however, no mention of this manuscript was included in the Introduction and Lines 71-74 were directly taken from it with no changes. The main point of the study which is to identify fish species from the Sea of Galilee and surrounding regions using DNA barcoding to build a reference database for effective management/conservation does not come across strongly. In other words, the exact reason for this study other than building a database is not explicitly clear, is it for the management of invasive species, monitoring of native species? The link to this being mentioned earlier in the introduction needs to be made clearer towards the end of the introduction.
Response: see below a point-by-point response.

Corrections:
Comment: L50-L51: Remove sentence.
Response: Sentence was removed and replaced by the suggested one.

Comments: L55: Please add a reference for the last bit of this sentence and change the sentence to read as follows, “Despite freshwater habitats only comprising of 1% of the Earth’s surface water, they support 43% (13 000 species) of the world’s freshwater fish [2,5], rich in biodiversity across all trophic levels [reference, e.g. Vörösmarty et al., 2010; Carpenter et al., 2011]”.
Response: sentence was changed as suggested.

Comment: L55-L57: Remove this sentence, as the content has been incorporated in

	the previous sentence at the beginning of line 55. Also, according to Leveque et al. (2007), only freshwater species and peripheral species add up to 13 000 and 15 000 if brackish water fish are included. According to the authors for this review, “15 000 freshwater fish species are known around the world”. This is incorrect, please change the sentence to read either as “13 000 freshwater fish species” or “15 000 species occurring from fresh to brackish waters”.<br>Response: 13,000 was used.<br><br>Comment: L51-L54: Move to the end of the sentence of L55 to read as follows: “Despite freshwater habitats only comprising of 1% of the Earth’s surface water, they support 43% (13 000 species) of the world’s freshwater fish [2,5], rich in biodiversity across all trophic levels [e.g. Vörösmarty et al., 2010; Carpenter et al., 2011]. As a result of human dependence on water, either for direct uses like irrigation and drinking or indirect uses like hydro-electrical energy production and food fishing, many freshwater fish species have become highly endangered [1, 6-9]” L57-L59: Remove sentence.<br>Responses: lines 51-59 were re-arranged and revised as recommended by the reviewer. References were added as requested.<br><br>Comment: L60-L62: Include a reference and change sentence to read as follows: “Effective management for the assessment of freshwater fish biodiversity and population status is therefore a priority, however, can be considered a great challenge.<br>Response: Text was revised as suggested, reference for this sentence was included.<br><br>Comment: L62-L65: I would not include reference number 8 (Lind et al., 2012) and number 10 (Leprieur et al., 2008) in your in-text citation for this sentence. Reasons being, the focus is still on a global point of view and you only mention fish invasions in the next sentence (L65-L66).<br>Response: The reference to Leprieur et al., 2008 was removed, however the reference to Lind et al., 2012 was kept as it does not refer specifically to fish invasions and is more general.<br><br>Comment: L65-L66: Change sentence to read as follows: “Further human derived pressure on natural fish diversity has also been documented due to introduction and invasion of exotic fish species”. Remove reference 12 (Goren and Ortal, 1999) and 14 (Ribeiro and Leunda, 2012) in your in-text citation, as again you are focusing on a global point of view here and focus specifically on Israel and the Mediterranean freshwater ecosystems in the next paragraph.<br>Response: Text was revised, and selected references were removed as suggested.<br><br>Comment: L67-L68: Insert the word “arid” before “climatic conditions.<br>Response: added.<br><br>Comments: L69-L77:<br>Comment: Insert the catchment area size in brackets to read as follows: “biggest surface freshwater reservoir in the middle east (ca. 170 km2)”.<br>Response: Catchment area size was added in brackets.<br><br>Comment: Change “are highly regulated” to “it is highly regulated”.<br>Response: Text was changed as suggested.<br><br>Comment: Include reference 12 (Goren and Ortal, 1999) in the in-text citation “[16,17]”.<br>Response: Reference was included.<br><br>Comment: L71: Instead of using the term “exotic” I would opt for using “non-native”. Also insert the word “freshwater” before “fish”<br>Response: Comment was accepted, and text changed accordingly.<br><br>Comment: L72: Remove reference 12 and 18 from in-text citation as this sentence is more specifically mentioned in reference 19.<br>Response: Requested references were removed.<br><br>Comment: L72-L74: Include references for this sentence.<br>Response: A reference for this sentence was added

Comment: L74: When including the common and scientific name for the first time, first write out the common name followed by the scientific name with the original author/identifier in brackets. Change to read as follows: “The native cyprinid Kinneret bleak Mirogrex terraesanctae (Steinitz, 1952)”.
Response: Changed here and throughout text that species are first mentioned properly. According to taxonomy conventions, the original identifier of the species was written in brackets when genus/species name were changed since first identified but without brackets when the original identification name was kept to date.

Comment: L75-L76: Change sentence to read as follows: “From the native cichlids Galilee tilapia or St. Peter’s fish Sarotherodon galilaeus (Linnaeus, 1758) is the most abundant”. Also can you specify how they are abundant? By number or biomass as was done in the previous sentence for the cyprinid.
Response: Text was revised as follows: “Based on fishery landings, the native cichlid, Galilee tilapia, or St. Peter’s fish Sarotherodon galilaeus (Linnaeus, 1758), is abundant, most valuable and sought after."

Comment: L77: Scientific names are predominantly used over common names once the species has been introduced. Also, S. galilaeus African distribution is more centralised and western, therefore, please change sentence to read as follows: “While M. terraesanctae is endemic to Israel, S. galilaeus is widespread in central and western Africa”. Please remove reference 21 (Lemoalle and de Condappa, 2010) from the in-text citation for this sentence, as this reference does not refer to S. galilaeus African distribution which is of focus here but rather to the Volta basin (west coast of Africa).
Response: Text was changed as suggested and checked that scientific rather than common names are used throughout manuscript. Reference 21 was removed.

Comments L78-L82:
Comment: Please rephrase sentence to read as follows: “Non-native species, which were unintentionally introduced, include the European eel Anguilla (Linnaeus, 1759) that hitchhiked with mullet fry caught in coastal estuaries and stocked into the lake, the hybrid tilapia Oreochromis niloticus (Linnaeus, 1758) x Oreochromis aureus (Steindachner, 1864) that escaped from aquaculture ponds and the peacock bass Cichla kelberi (Kullander and Ferreira, 2006) that was probably released in the lake by hobbyists [22]”.
Also, please include references for the introductions of the European eel and the hybrid tilapia in the in-text citation as currently you only have a reference for the peacock bass.
Response: Text was updated by relevant references and revised.

Comments L82-L83:
Comment: Here you listed Gambusia affinis as “exotic” stipulating that it is not harmful to native species, however, this is not the case as G. affinis is listed as one of the world’s worst non-native invasive species (Lowe et al., 2000). Please rephrase this sentence to read as follows: “Other non-native species, which were intentionally introduced and invasive include the mosquito fish Gambusia affinis (Baird and Girard, 1853) which were introduced to control mosquitoes. Please also include a reference for this sentence.
Response: The sentence was revised. A reference for the introduction of Gambusia fish was added
Comments L83-L91: Please rephrase this sentence to read as follows: “Nevertheless, significant non-native species which were introduced to enhance commercial fishing included the common carp Cyprinus carpio (Linnaeus, 1758), the silver carp Hypophthalmichthys molitrix (Valenciennes, 1844), the thin lip grey mullet Chelon ramada (Risso, 1827) and the flathead grey mullet Mugil cephalus (Linnaeus, 1758)”.
Response: Text was revised as suggested.

Comment: Please note that the genus name “Liza” has been updated to “Chelon”. For assurity, please check that all species mentioned in this paper are up to date by reviewing them in the Catalogue of Fishes Database.
Response: We checked all species names mentioned in this manuscript by reviewing them in the Catalogue of Fishes Database, to our knowledge all species names are currently up to date.

Powered by Editorial Manager® and ProduXion Manager® from Aries Systems Corporation

Comment: Also, please include a reference for this sentence.
Response: References for introduction of non-native species mentioned here were added.

Comment: L86-L88: Please include a reference for this sentence.
Response: A reference for this sentence was added.

Comment: L90-L91: Change “estimate” to “estimates” and change “Kinneret bleak” to “M. terraesanctae”.
Response: Text was changed as suggested.

Comment: L93-L94: Rephrase sentence to read as follows: “In recent years, DNA polymorphism, a more reliable and accurate method, is increasingly used for taxonomic and ecological research”.
Response: Comment was accepted, and text changed.

Comment: Please include a references for this sentence by providing other examples of past literature where DNA polymorphism has been used successfully.
Response: A few example references were added to text.

Comment: L94: Change “sequence” to “sequences”.
Response: Comment was accepted, and text changed.

Comment: L95: Remove “species” before “DNA barcoding”.
Response: Comment was accepted, and text changed.

Comment: L98: After “environmental water samples, include “known as E-barcoding”.
Response: Comment was accepted, and text changed.

Comment: L98-L99: Elaborate more on what reference databases have been used and are currently being used successfully, e.g. BOLD. Explain the process in more detail. This will strengthen your argument and lead nicely on to your aim for the study.
Response: The process of molecular identification of specimen was explained in more detail, and the Barcode of Life Data systems was presented and described shortly.

Comment: L101: Change “question” to “questions”.
Response: Comment was accepted, and text changed.

Methods
The experiments or intervention used was DNA barcoding. DNA barcoding was appropriate for addressing the research question. However, the data collected was not interpreted accurately. I found it very hard to follow the protocols and feel that another researcher would battle to reproduce this study based on the methodology reported. For instance, sampling methods are not clear, number of samples collected for each species per site are not clear as well as how many successful sequences were used from this sample size. No mention of whether the fish collected were stored in formalin for taxonomic identification as well as no mention of photos of samples for each species were taken to upload with BOLD. References were not provided or corresponded to the authors point. Data analysis section needs work. Please could the authors rectify the above and include them into the materials and methods section.
Response: see below a point-by-point response.

Comment: L106-L108: Firstly, I would not recommend saying “in the context”, rather say “from the Sea of Galilee and surrounding areas”. Secondly, what is this extensive sampling scheme that was carried out to obtain samples from each species that could be found in the Lake? For instance, how were these fish collected? Electrofishing? Seine netting? It is important to incorporate this detail here in the beginning of your materials and methods section as well as the years in which this was done (2014 and 2015?) to correspond to your permit numbers.
Response: Text was changed as suggested. Details regarding the sampling scheme are explained in more detail in the following lines as suggested and addressed in the next comment. Information on the permits for sampling was added in the ethical approval section.

Comments: L108-L113: What do you mean by “12 sampling attempts” and “few additional attempts”? This gives me the impression that collection was done on 12 separate occasions but nothing was collected by using the word ‘’attempts”. Also, Borovski et al. (2018), does not mention anything about “12 sampling attempts” but rather provides more detail in a table and figure of the location of the sites sampled from. I would recommend changing this sentence to read as follows: “The Sea of Galilee, the main sampling site, and surrounding sites such as Asi stream (one of Beit She’an streams, south to the lake and part of the eastern watershed of Israel), Ein Afek pools (close to Acre, i.e. Akko, part of the western watershed of Israel) and Ein Te’o spring (close to Hula Lake, northern to Lake of Galilee and part of the eastern watershed of Israel) (Fig. 1) was sampled during 2014 and 2015 to collect samples of species by means of “seine netting” (whichever method you used for collection) that predominantly occur in this region of Israel for DNA barcoding. Numbers of individual samples collected for each site and species are recorded in Table 1”.
I would also recommend including a map (Fig.1) of your sites including all the small town names and streams as mentioned above in brackets on the map for a point of reference and in Table 1 include columns labelled “site” (Sea of Galilee/Kinneret, Asi stream, Ein Afek pools, and Ein Te’o spring) and “sample size” indicating the number of samples collected for each species for each site.
Response: Sampling was done on 12 different occasions, all ‘attempts’ yielded fish for sampling. Details about sampling method, locations and yields were explained in more detail based on the recommendation for text revision given here. Additionally, a new Table 1 was added to provide details about number of fish sampled compared to number successfully barcoded from each species and from each location. Instead of providing a map as suggested we refer to figure 1 in Borovski et al. (2018) were this data was already published, as we would like to avoid publishing the same data twice. We however, modified the map in Figure 2 to include sampling site names so this information is readily available in this manuscript as well.

Comment: Within table 1 change the column “N samples” to “N samples barcoded”. This will give us a clear indication of the number of samples per species per site that were successfully barcoded from the sample size collected for each species.
Response: Within the table (now table 2) ‘N samples’ was changed to ‘N barcoded samples’.

Comment: Be consistent of your labelling and mentioning of Sea of Galilee rather than “Lake Kinneret” as this was in brackets in the introduction section (L69).
Response: Text was reviewed throughout to ensure that ‘Sea of Galilee’ is used rather than ‘Lake Kinneret’.

Comment: L113-L114: When referring to fish being taxonomically identified to species level, were they identified in the field and returned to the environment or where they sacrificed and taken for identification under lab conditions (preserved entire sample in formalin)? Please specify in this sentence. Also, under DNA barcoding methods of collection, it is important to photograph each sample and upload this photograph on the BOLD database with it its barcode index number (BIN number), was this done? If so, please include this information. Furthermore, why specifically fin clips and not a muscle tissue sample? According to Weight et al. (2012) (DNA barcoding fishes, a good reference to include here), even though fin clips work, the most effective material is a small muscle biopsy from the specimen taken from the right side of the body. Please explain in detail why a fin clip over muscle tissue was done. Lastly, if fin clips were taken, was there evidence of the fish surviving if returned to its habitat and not preserved? Were there any infections caused which may have made the individual more susceptible to predation?
Response: Text was revised to include more information about sampling procedures and fish handling. Text is now informative with regards to all these points. In most cases fish were returned to their environment after identification in the field and sampling of a fin clip. We have experience with fish (including critically endangered fish) in lab conditions showing that after recovery from anaesthesia, fish return to normal behaviour and their survival is mostly unaffected. As fins easily regenerate and fish integument is unharmed, fin clip samples were chosen over muscle and in our experience, fin clips prove to be effective for DNA isolation and barcoding. Some of the fish were sacrificed photographed and deposited in the National Natural History

Powered by Editorial Manager® and ProduXion Manager® from Aries Systems Corporation

	Collections of the Hebrew University, in the new Table 1, the information of number of specimens that were deposited and their Museum catalogue numbers was added. This data was also made available in BOLD.<br><br>Comment L118: Change to read as follows: "DNA extraction, polymerase chain reaction (PCR), purification and sequencing". Combine your original sub-headings of "DNA extraction" and "PCR products and sequencing of PCR products" into one paragraph.<br>Response: The two sub-sections were combined under the sub-heading of "DNA extraction, polymerase chain reaction (PCR), purification and sequencing".<br>Response: done as suggested.<br><br>Comment L119-L120: For the salting out method, a reference, Martinez et al. (1998) is provided, however, this citation refers to the salting out method from fish whole blood. Ideally, it would be better to provide a reference that directly links this method for extracting DNA from fin clips of fish as was stipulated, for example Taggart et al. (1992) or Sunnucks and Hales (1996) which in most cases for other references, related to this method, modified it from Miller et al. (1988).<br>Response: A reference for the salting out method from fin samples was added.<br><br>Comment L120-L126: Delete. Not necessary to go into detail if you provide the correct reference for the salting out method and protocol in which you followed.<br>Response: These lines were deleted.<br><br>Comment L132-L135: The primers indicated in the manuscript are not the same primers used as Ward et al. (2005). They used FishF1, FishF2, FishR1, FishR2 and the primers included in the manuscript were coxla_fw, coxla_rv. Hence, please do not indicate that they were "adopted" from Ward et al. (2005) but rather provide a reference that used the same primers as was indicated in this manuscript. Change sentence to read as follows: "A partial fragment of (include the number of base pairs here) of the mitochondrial cytochrome c oxidase subunit 1 gene (COI) was amplified by PCR using the primers …….".<br>Response: We used primers FishF1 and FishR1 developed by Ward et al. (2005) under the names cox1a_fw and rv, which were internal names used in our lab. The sequence, however, is identical. We therefore changed the primer names in the text to match the original names from Ward et al. Additionally text was revised according to the comment.<br><br>Comment L138: Please include the amount of water used to complete the 20µl reaction volume and change "PCR profile" to "PCR thermal protocol".<br>Response: We added the amount of water used in our PCR protocol to complete to 20 µl, and changed "PCR profile" to "PCR thermal protocol" as suggested.<br>Comment L139-L142: Please include the reference that you used this protocol from or modified it from.<br>Response: We developed a thermal protocol in our lab that was effective for amplification of products by these primers and described it in the text, therefore no reference was provided.<br><br>Comment L144-L146: Change sentence to read as follows: "Successful PCR products were purified using an Exonuclease I-Shrimp Alkaline Phosphate (ExoSAP-IT) method (USB, Cleveland, OH) and sequenced using BigDye V3.1 (add manufacturers name) terminator chemistry in the forward and reverse direction on an ABI PRISM 3730xl DNA analyser (add manufacturers name) following the manufacturers protocol".<br>Response: Text was revised as suggested and manufacturers of materials and instruments were provided where they were missing.<br><br>Comments: L148:L159<br>For the data analysis section, methodology processes are not clearly explained and a lot more work needs to be done here. For instance, under this section it needs to be mentioned that successful sequences for each species was deposited into the Barcode of Life Data (BOLD) System (Ratnasingham and Hebert, 2007; https://www.boldsystems.org) under the project name LKCOX (remove this sentence from results section under molecular barcoding, and add it under data analysis).<br>Response: Text was revised as requested, the relevant sentence was moved here

from the results section, and blended with the existing revised text.

Comment: In addition, once deposited, what exactly was the role of the BOLD database? Were the sequences from this study used to compare against available reference database sequences to find the closest/nearest matching species which was displayed as a percentage probability (0-100%)? Also, for each species, what was the number of sequences submitted into BOLD for comparison (is this the N samples column in Table 1)? Next, were the publicly available sequences with a >98% similarity/match downloaded from the BOLD reference database (and how many) to include with your sequences in running the haplotype analysis. In the results section, L196-L197 includes the above information, move this sentence here to the data analysis section. Also, L204-L206 of the results section needs to be moved here to the data analysis section.
Response: This section was rearranged, including the requested lines from the results section, as suggested. Now, the text includes all requested information. Regarding the number of sequences submitted for each species – this is described in Table 1 and referred to here.

Comment: How did you run the haplotype (phylogenetic) analysis? How did you get the relative number of haplotypes for each species? Did you use the software DnaSP?
Response: After alignment of sequences, we manually inspected polymorphisms and named polymorphic sequences of the same species as different haplotypes. This is now stated more clearly in the text.

Comment: Was the neighbour joining tree rooted or unrooted for both Figure 1 and Figure 2? Please provide a reference for the bootstrap method of 1000 replicates.
Response: Trees in both figures were unrooted, this is now stated in the methods section and also in both figure legends. A reference for the bootstrap method was provided.

Comment: How was average pairwise haplotype distances calculated? Did you use ARLEQUIN software?
Response: The average pairwise haplotype distances was calculated by summing up all relevant pairwise K2P distances, calculated using MEGA software (described in the text), and dividing them by the number of comparisons.

Comment: Please stipulate what version of the BioEdit software was used and indicate what the total length of base pairs was used or trimmed to by the software.
Response: This data is now added in the relevant places in the text.

Comment: Considering relevant species were sampled from four sites (mainly the Sea of Galilee), why was a haplotype network not included to show if some species indeed differed according to site based on their haplotype groupings?
Response: This suggestion applies mainly to the Garra complex, for which we analyzed samples from different locations showing different haplotypes. Most species have different haplotypes within the lake population (including Garra), leaving such an analysis less interesting. Nevertheless, this point for Garra, including from other countries, in our opinion, is presented in a more specific and legible way than a haplotype network in Figure 2.

Results
Results are very interesting! Figures and tables are clear and readable, however Figure 2 is not of great quality and more information needs to be incorporated in to the map (Fig 2A; see below corrections for further recommendations). Table 2 legend should be above the table and not below it.
Response: see below a point-by-point response.

Corrections:
Comment: I would recommend for Figure 1 to exclude the K2P distances combined here and provide them as separate tables as has been done for Table 2, just separate it according to family using sub-headings.
Response: We prefer to leave the K2P distances within the figure, instead of separating them in three tables. Separate tables will be small and require scrolling among them and back in reference to Fig. 1, which, we think, will be less convenient to

Powered by Editorial Manager® and ProduXion Manager® from Aries Systems Corporation

the reader. This way the distances and phylogeny can be viewed simultaneously to demonstrate better the points of interest.

Comment: Also correct acronyms for Figure 2 and Table 2 (see below corrections for further recommendations).
Response: This was corrected as per the below comments.

Comment: L169-L170: Delete.
Response: Deleted.

Comment: L171: Are there 25 fish species reported in the Lake? From the in text references reported for this sentence, Goren and Ortal (1999) reported 19 native species in the Lake; Ostrovsky et al. (2014) reported 19 native species in the lake; and I did not come across the number of native species for Ostrovsky et al. (2014) fisheries management chapter. Even Borovski et al. (2018) reports 19 native species and 8 exotic species, which gives a total of 27 and not 25. Please clarify.
Response: In Ostrovsky et al. (2014) there are 18 native species in the lake (19 stated, probably after Goren and Ortal, however, in the table actually detailing the lake species (table 16.1 in Ostrovsky et al., 2014), only 18 species are listed). In comparison to Goren and Ortal (1999), the missing species is Nun galilaeus, which was native to the Hula valley and suspected to have migrated into the lake, but anyways, since the 1950's this species is considered extinct. Therefore, we updated our introduction to report 18 native fish species and changed text here to be more specific by reporting how many of the native species we obtained barcode sequences for, and how many non-native species.

Comment: L172-L176: Please provide the original author/identifier in brackets for all species mentioned in these lines. Change “schemes” to “sites”.
Response: author/identifiers were added to each species as appropriate.

Comment: L177: At the end of the sentence add Table 1 in brackets.
Response: Added.

Comment: L177-L179: Rephrase this sentence to read as follows: “Additionally, 20 sequences were analysed from two fish species, H. molitrix (19 sequences) and O. aureus (1 sequence), that were suspected as hybrids with aquaculture fish (hereafter referred to as ‘suspected hybrids’) (Table 1)”.
Response: Text was revised as suggested.

Comment: L179-L180: Delete and move to data analysis section under materials and methods.
Response: This sentence was moved to m&m, data analysis section.

Comment: L181-L182: As previously mentioned, can the authors make clear how many specimens were collected for each species, and out of this sample size, how many were successfully sequenced and add this to Table 1 as some sequences may have been unsuccessful. Also, at the end of this sentence add Table 1 in brackets.
Response: The new Table 1 includes the requested information.

Comment: L183-L184: At the end of the sentence add Table 1 in brackets.
Response: Added, but now Table 2.

Comment: L186: Add the “number” of species where new barcodes were added to existing barcodes in BOLD.
Response: The number of species with novel barcodes was already in the sentence, however, to clarify, we changed the sentence to read as follows: “For ten species with barcodes existing in BOLD, 12 new barcodes were added. These added haplotypes were always those with the lower frequency in our data (Table 2)."

Comment: L188-L190: Change Table 1 legend to include sites and sample sizes for each species.
Response: This information is now detailed in the new Table 1.

Comment: L196-L197: Delete and move to data analysis section under materials and

Powered by Editorial Manager® and ProduXion Manager® from Aries Systems Corporation

methods.
Response: This sentence was deleted here and moved to data analysis section of methods.

Comment: L197-L199: Specify the exact number of species where molecular identification agreed with the original morphological identification.
Response: We added that for 19 out of 22 molecular identification agreed with the original morphological identification.

Comment: L199-L201: Delete and move to data analysis section under materials and methods.
Response: This sentence was deleted here and moved to data analysis section of methods.

Comment: L204-L206: Mention how many species were in disagreement. Please rephrase the sentence to read as follows: “Three species were found to have disagreements between morphological and molecular barcoding identification. Further investigation was carried using all available COI barcode records from BOLD of query and related species”.
Response: Text was revised as suggested, and the methods part was moved into data analysis section of methods.

Comment: L206: Cannot start a sentence with the genus name of a scientific name abbreviated, need to write it out in full, “Acanthobrama”. Delete “we”.
Response: Text was revised as suggested.

Comment: L211-L214: Remove from results section and add to discussion section. You are only stating the main points of results and should not discuss them in depth in this section.
Response: Text was revised as suggested.

Comment: L215-L217: Please rephrase the sentence to read as follows: “Three Gambusia affinis samples collected, all with the same haplotype (GaA), was identified by BOLD as G. holbrooki (100% fit), followed by G. affinis (99.8% fit).
Response: Text was revised as suggested.

Comment: L217-L220: Please delete the last part of this sentence and move to discussion section. Please rephrase sentence to read as follows: “Comparison to all available G. holbrooki and G. affinis records from BOLD indicated that GaA haplotype clusters together with G. holbrooki records”.
Response: the last part of the sentence was deleted and moved to the discussion section.

Comment: L222: Remove “identification module”.
Response: deleted.

Comment: L223-L224: Rephrase this sentence to read as follows: “This disagreement will be discussed further later under its own sub-section”.
Response: Text was revised as suggested.

Comment: L225-L226: Rephrase sentence to read as follows: “Molecular identification using BOLD for samples of fish suspected as hybrids provided genetic evidence to support hybridization.
Response: Text was revised as suggested.

Comment: L228: Change “into” to “in”.
Response: Text was revised as suggested.

Comment: L229-L231: Delete this sentence, starting from “This is consistent”.
Response: Text was revised as suggested.

Comment: L234-L235: Add a full stop after “in both directions” and delete the remainder of the sentence starting from “again”.
Response: Text was revised as suggested.

Comment: L236: Change “another” to “further”.
Response: Text was revised as suggested.

Comment: L239-L252: For genetic distance from barcoding data, I would start off with discussing the results from the neighbour-joining phylogenetic tree and incorporating the K2P genetic distances with this.
Response: This paragraph begins with a general statement about the scale of the K2P distances, followed by a general description of the phylogenetic tree. In the next paragraph, a more detailed description of the interesting results in the tree are combined with the K2P genetic distances. We believe this is a reasonable and logical way to present the results.

Comment: In figure 1, for certain haplotypes there as asterisk next to them. In the Figure 1 legend, please indicate what these asterisks mean.
Response: Description of the asterisks meanings were added to figure 1 legend.

Comment: L260-L263: Please rephrase this sentence to read as follows: “Unexpected results were, however, found within the Cichlidae clade of the neighbour-joining phylogenetic tree. Oreochromis aureus (Haplotype OaA) clustered together with Sarotherod galilaeus (Haplotype SgA), a species belonging to a different genus, rather than with O. niloticus (Haplotype OaB) with which its genus is shared”.
Response: Text was revised as suggested.

Comment: L263-L267: Please rephrase this sentence to read as follows: “Support from K2P distances between O. aureus and S. galilaueus (0.0095) is smaller in comparison to distances between other cichlids of Lake Galilee belonging to different genera, and even smaller compared to cyprinids belonging to different genera (0.08-0.23) (Fig. 1)”.
Response: Text was revised as suggested.

Comment: L267-L268: Delete this sentence here and move it to discussion section.
Response: This suggestion was followed.

Comment: L269: Change “G. rufa” to “Garra rufa”.
Response: Text was revised as suggested.

Comment: L270: delete “we find”.
Response: Deleted.

Comment: L271-L273: Please rephrase this sentence to read as follows: “Identical K2P distances was, however, found between Acanthobrama telavivensis and Mirogrex terraesanctae, formerly known as A. terraesanctae and later moved to a separate genus”. Reference [36] has been removed here as in the result section you do not include in text references.
Response: This suggestion was followed.

Comment: L273-L274: Please delete this sentence, starting from “This relatively” and move to the discussion section.
Response: This suggestion was followed.

Comment: L277: Delete “As mentioned before”. Start sentence with “Comparison of Garra rufa haplotypes”.
Response: Text was revised as suggested.

Comment: L281-L282: Delete “Samples identified morphologically as”. Start sentence with “Garra rufa fish”.
Response: Text was revised as suggested.

Comment: L283: After Ein Te’o put “north of the Sea of Galilee” in brackets.
Response: Text was revised as suggested.

Comment: For Figure 2A, can the authors please explicitly label Asi stream, Ein Afek and Ein Te’ o stream in text in the correct location on the map to make these locations clearer in relation to the haplotypes. Also, include labels for eastern and western

watersheds of Israel on the map (Fig. 2A). Furthermore, can you give Haplotype GrD its own circle and colour and make GrA haplotype in the Sea of Galilee the same colour as in Ein Te'o (the colours on the map look different).
Response: Figure 2 was edited, site names were included (except for Ein Afeq, since no Garra fish were sampled there), eastern and western watersheds were not marked on the map since all sampled fish are from the eastern watershed of Israel. Haplotype GrD received its own circle and colour and we made sure that GrA haplotype in the Sea of Galilee is the same colour as GrA in Ein Te'o. Furthermore, map resolution was improved.

Comment: L284: Make "the" prior to Sea of Galilee samples capitalized.
Response: Text was revised as suggested.

Comment: L284-L287: Please rephrase this sentence to read as follows: "The Sea of Galilee samples had five fish from Haplotype GrA, two fish from Haplotype GrC and one fish from Haplotype GrD. All three fish from Ein Te'o shared Haplotype GrA and all three fish from Asi stream shared Haplotype GrB (Fig. 2A).
Response: Text was revised as suggested.

Comment: L289-L298: Please rephrase Figure 2 legend to read as follows: "Sampling sites and clustering of Garra spp. Samples based on COI haplotypes. Acronyms of populations are: GrA – Garra rufa from Haplotype A; GrB – Garra rufa from Haplotype B; GrC – Garra rufa from Haplotype C; GrD – Garra rufa from Haplotype D; Gg – Garra ghorensis from the southern basin of the Dead Sea; Gj – Garra jordanica from the northern basin of the Dead Sea; GnA – Garra nana from Haplotype A; GnB – Garra nana from Haplotype B. (A) Map showing the sampling sites in a regional context. Different Garra spp. Haplotypes were denoted by differently coloured circles in each sampling site on the map of Israel, Jordan and Syria regions. (B) A dendrogram constructed for Haplotypes obtained from samples collected in this study alongside Haplotypes mined from BOLD (see Process ID's in Table S1). Next to the edges, haplotype acronyms are given as indicated above. Numbers next to edges denote percent bootstrap support. Colours of Haplotype text match the colour of the circles on the map (A)".
Response: Text of the figure legend was revised as suggested.

Comment: L300: Delete "To put into context the Garra spp. Sampled from Israel,". Start sentence with "Sequences of 16 Garra spp. From".
Response: Text was revised as suggested.

Comment: L301: Delete "a" before "geographic location".
Response: Deleted.

Comment: L302: Add "assigned" before "to them" and add "see" before "Table S1" in brackets. Add "neighbour-joining" before "phylogenetic tree (Fig 2B)".
Response: Text was revised as suggested.

Comment: L305: After 0.0381 in brackets, add "; Table 2). After "were found in fish from" add "the Sea of Galilee and a northern location in Syria (Fig 2A). Delete "two isolated locations" and "GnA, the G. nana haplotype found in the Sea of Galilee was found also in samples from a northern location in Syria".
Response: These lines were rephrased to read as follows 'The two haplotypes of G. nana (GnA and GnB) were relatively distinct from one another (K2P = 0.0381; Table 3). GnA, was found in fish from the Sea of Galilee and the more northern location in Syria, while GnB, was found in fish from an isolated location more southern in Syria (Fig 2A).'.

Comment: L308-L311: Please rephrase this sentence to read as follows: "Israel G. rufa samples from the Sea of Galilee and Ein Te'o had Haplotypes GrA, GrC and GrD. These Israel G. rufa Haplotypes clustered with BOLD Haplotypes of G. jordanica (Haplotype Gj), fish sampled from streams in the northern basin of the Dead Sea in Jordan (Fig. 2B)".
Response: Text was revised as suggested.

Comment: L311-L314: Please rephrase this sentence to read as follows: "Close by, yet

Powered by Editorial Manager® and ProduXion Manager® from Aries Systems Corporation

separated, in the phylogeny, was G. rufa fish, Haplotype GrB, from Asi stream, suggesting again greater similarity to G. jordanica, but some genetic separation from the Sea of Galilee population".
Response: Text was revised as suggested.

Comment: L318: Insert "GrAsia –" before "Turkey, Iran and Iraq" in the brackets.
Response: Inserted.

Comment: L318-L320: Delete this sentence starting from "Thus, the results indicate that" and move it to the discussion session.
Response: Deleted and moved.

Comment: L321-L327: Figure legend for Table 2 should go above the table and not below the table. Provide a reason as to why Gr North has an asterisk sign and the other locations do not. Change acronyms for populations to read as follows: "Gr North – Garra rufa from Sea of Galilee and Ein Te'o of northern Jordan valley; GrAsi – Garra rufa from Asi stream of Beit she'an valley; Gj – Garra jordanica from the northern basin of the Dead Sea; Gg – Garra ghorensis from the southern basin of the Dead Sea; Gr Asia – Garra rufa from Asian countries including Turkey, Iran and Iraq; GnA – Garra nana from Haplotype A; GnB – Garra nana from Haplotype B".
Response: Figure legend for this table (now Table 3) was placed above the table and revised as suggested.

Comment: L333-L337: Please rephrase this sentence to read as follows: "The Israeli G. rufa fish (Gr North) are somewhat genetically divergent from G. rufa fish from eastern and norther Asian countries (Gr North vs Gr Asia mean K2P distances = 0.0025 vs 0.0462; Table 2)".
Response: To clarify the point of interest, the text was revised to read as follows: 'The Israeli G. rufa fish, are genetically divergent from G. rufa fish from farther eastern and northern Asian countries (Mean K2P between Gr North and Gr Asia = 0.0462, and between Gr Asi and Gr Asia = 0.0469; Table 3).". The text following these lines was also revised similarly to improve clarity.

Comment: L340-L341: Delete "from the Northern Jordan valley" and replace it with "(Gr North)".
Response: Text was revised as suggested.

Comment: L343: Insert "south Asi stream of" before "Beit she'an valley". Delete "(Asi stream)" and replace it with "(Gr Asi)".
Response: Text was revised as suggested.

Comment: L344-L345: Delete "in the Hula valley".
Response: Text was revised as suggested.

Comment: L350: insert "(mean K2P of 0.0587) after "from Asia".
Response: Text was revised as suggested.

Comment: L351: Rephrase to read as follows: "G. rufa (mean K2P of 0.014) (Table 2)".
Response: Text was revised as suggested, mean K2P should be 0.0414, we assume this was the reviewer's intention.

Discussion
The discussion section is almost a repetition of the authors results section. More evidence needs to be provided here to support your results. Elaborate by including more examples of relevant recent literature to strengthen your arguments. Results are interesting, however a lot more work needs to be done in this section. Also, it is important to include any limitations that were raised during this this study and should be highlighted and discussed here by providing ways in the future as to how it can be avoided or improved on. Also, there is no conclusion section, which is very important to drive your take home message for this study. For instance, in the conclusion section by adding a how this study could contribute to the conservation of Israel's native species and identification of hybrid and non-native invasive species through DNA barcoding would be a good as well as how it will improve in the future from this perspective. Furthermore, before discussing the G. rufa complex in the discussion, authors need to

Powered by Editorial Manager® and ProduXion Manager® from Aries Systems Corporation

	discuss the other disagreements that were found from your results, namely A. lissneri and A. telavivensis; G. holbrooki and G. affinis; hybrids; Cichlidae; and Acanthobrama-Mirogrex, in more detail by referring to or by providing recent, relevant and supportive literature. In the results section, I indicated which sentences need to be moved to the discussion section relative to the above sentence as they should not be present in the results section.<br>Response: the discussion was considerably rewritten incorporating all the suggestions of the reviewer including a conclusions section. Also, see below a point-by-point response to specific comments.<br><br>Corrections:<br>Comment: L361: Reference [25], i.e. Ward et al. (2005) does not directly refer to DNA barcoding as being an important part of BOLD, perhaps rather refer to reference [40].<br>Response: Sentence was changed to emphasize the utility of fish DNA barcoding including relevant references.<br><br>Comment: L361-L362: Please rephrase this sentence to read as follows: "In a previous study conducted by [39], the cichlids of Israel were barcoded with success".<br>Response: Text was rephrased as suggested.<br><br>Comment: L362-L363: Please rephrase this sentence to read as follows: "In this study, focus was given to the fish found in the Sea of Galilee and surrounding water bodies in the region, including species other than cichlids".<br>Response: Text was rephrased as suggested.<br><br>Comment: L367: After "12 species", add "mostly belonging to the Cyprinidae and Cichlidae,".<br>Response: Text was rephrased as suggested.<br><br>Comment: L370: For references [24,40] did both studies focus on similar families as this study? Is this a fair comparison?<br>Response: References of studies on similar species in which similar distances were found were added.<br><br>Comment: L375-L377: Please provide a reference for this sentence.<br>Response: References for this sentence were added<br><br>Comment: L389: Please indicate how recent this isolation occurred in by means of a time frame.<br>Response: We added the dating of this isolation (~24,000 years ago) to the text, along with a supporting reference.
**Additional Information:**	
**Question**	**Response**
**Financial Disclosure**<br><br>Enter a financial disclosure statement that describes the sources of funding for the work included in this submission. Review the submission guidelines for detailed requirements. View published research articles from *PLOS ONE* for specific examples.<br><br>This statement is required for submission and **will appear in the published article** if the submission is accepted. Please make sure it is accurate.	The study was funded by the Chief Scientist of the Israeli Ministry of Agriculture and Rural Development under Grant #186-0001-11 to LD and GH. https://www.moag.gov.il/en/Ministrys%20Units/Chief%20Scientist/Pages/default.aspx. The funders had no role in study design, data collection and analysis, decision to publish, or preparation of the manuscript.

**Unfunded studies**<br>Enter: *The author(s) received no specific funding for this work.*<br><br>**Funded studies**<br>Enter a statement with the following details:<br>• Initials of the authors who received each award<br>• Grant numbers awarded to each author<br>• The full name of each funder<br>• URL of each funder website<br>• Did the sponsors or funders play any role in the study design, data collection and analysis, decision to publish, or preparation of the manuscript?<br>• **NO** - Include this sentence at the end of your statement: *The funders had no role in study design, data collection and analysis, decision to publish, or preparation of the manuscript.*<br>• **YES** - Specify the role(s) played.<br><br>* typeset	
**Competing Interests**<br><br>Use the instructions below to enter a competing interest statement for this submission. On behalf of all authors, disclose any competing interests that could be perceived to bias this work—acknowledging all financial support and any other relevant financial or non-financial competing interests.<br><br>This statement **will appear in the published article** if the submission is accepted. Please make sure it is accurate. View published research articles from *PLOS ONE* for specific examples.	The authors have declared that no competing interests exist.

Powered by Editorial Manager® and ProduXion Manager® from Aries Systems Corporation

**NO authors have competing interests**<br><br>Enter: *The authors have declared that no competing interests exist.*<br><br>**Authors with competing interests**<br><br>Enter competing interest details beginning with this statement:<br><br>*I have read the journal's policy and the authors of this manuscript have the following competing interests: [insert competing interests here]*<br><br>* typeset	
**Ethics Statement**<br><br>Enter an ethics statement for this submission. This statement is required if the study involved:<br><br>• Human participants<br>• Human specimens or tissue<br>• Vertebrate animals or cephalopods<br>• Vertebrate embryos or tissues<br>• Field research<br><br>Write "N/A" if the submission does not require an ethics statement.<br><br>General guidance is provided below. Consult the submission guidelines for detailed instructions. **Make sure that all information entered here is included in the Methods section of the manuscript.**	Fish sampling from protected nature reserves was done under special permits 2012/38733 and 2014/40233 for sampling protected wildlife as reviewed and approved by the Israel Nature and National Parks Protection Authority. Some samples were obtained from catches of a commercial fishing vessel.

Powered by Editorial Manager® and ProduXion Manager® from Aries Systems Corporation

**Format for specific study types**<br><br>**Human Subject Research (involving human participants and/or tissue)**<br>• Give the name of the institutional review board or ethics committee that approved the study<br>• Include the approval number and/or a statement indicating approval of this research<br>• Indicate the form of consent obtained (written/oral) or the reason that consent was not obtained (e.g. the data were analyzed anonymously)<br><br>**Animal Research (involving vertebrate animals, embryos or tissues)**<br>• Provide the name of the Institutional Animal Care and Use Committee (IACUC) or other relevant ethics board that reviewed the study protocol, and indicate whether they approved this research or granted a formal waiver of ethical approval<br>• Include an approval number if one was obtained<br>• If the study involved *non-human primates*, add *additional details* about animal welfare and steps taken to ameliorate suffering<br>• If anesthesia, euthanasia, or any kind of animal sacrifice is part of the study, include briefly which substances and/or methods were applied<br><br>**Field Research**<br><br>Include the following details if this study involves the collection of plant, animal, or other materials from a natural setting:<br>• Field permit number<br>• Name of the institution or relevant body that granted permission	
**Data Availability**<br><br>Authors are required to make all data underlying the findings described fully available, without restriction, and from the time of publication. PLOS allows rare exceptions to address legal and ethical concerns. See the PLOS Data Policy and FAQ for detailed information.	Yes - all data are fully available without restriction

Powered by Editorial Manager® and ProduXion Manager® from Aries Systems Corporation

A Data Availability Statement describing where the data can be found is required at submission. Your answers to this question constitute the Data Availability Statement and **will be published in the article**, if accepted.<br><br>**Important:** Stating 'data available on request from the author' is not sufficient. If your data are only available upon request, select 'No' for the first question and explain your exceptional situation in the text box.<br><br>Do the authors confirm that all data underlying the findings described in their manuscript are fully available without restriction?	
**Describe where the data may be found in full sentences. If you are copying our sample text, replace any instances of XXX with the appropriate details.**<br><br>• If the data are **held or will be held in a public repository**, include URLs, accession numbers or DOIs. If this information will only be available after acceptance, indicate this by ticking the box below. For example: *All XXX files are available from the XXX database (accession number(s) XXX, XXX.*).<br>• If the data are all contained **within the manuscript and/or Supporting Information files**, enter the following: *All relevant data are within the manuscript and its Supporting Information files.*<br>• If neither of these applies but you are able to provide **details of access elsewhere**, with or without limitations, please do so. For example:<br><br>*Data cannot be shared publicly because of [XXX]. Data are available from the XXX Institutional Data Access / Ethics Committee (contact via XXX) for researchers who meet the criteria for access to confidential data.*<br><br>*The data underlying the results presented in the study are available from (include the name of the third party*	Sample collection data and barcode sequences were deposited in Barcode Of Life Datasystems (BOLD) under project name LKCOX. Accession numbers LKCOX001-19 - LKCOX217-19 https://www.boldsystems.org/index.php

and contact information or URL).<br>• This text is appropriate if the data are owned by a third party and authors do not have permission to share the data.<br><br>* typeset	
Additional data availability information:	

Powered by Editorial Manager® and ProduXion Manager® from Aries Systems Corporation

Manuscript Click here to access/download;Manuscript;KinneretBarcoding_PLoSOne_sub

Establishing and using a genetic database for resolving identification of fish species in the Sea of Galilee, Israel

Short title: DNA barcoding the fish species of the Sea of Galilee

Roni Tadmor-Levi[1,2], Tomer Borovski[2,†], Evgeniya Marcos-Hadad[2], James Shapiro[3], Gideon Hulata[4], Daniel Golani[1], Lior David[1,2,*].

[1] National Natural History Collections and Department of Ecology, Evolution and Behavior, The Hebrew University of Jerusalem, Jerusalem, Israel.

[2] Department of Animal Sciences, RH Smith Faculty of Agriculture Food and Environment, The Hebrew University of Jerusalem, Rehovot, Israel

[3] Fishery and Aquaculture Unit, Ministry of Agriculture and Rural Development, Beit-Dagan, Israel

[4] Department of Poultry and Aquaculture, Institute of Animal Science, Agricultural Research Organization (ARO), Volcani Center, Rishon LeZion, Israel

[†] Deceased

[*] Corresponding Author

E-mail: lior.david@mail.huji.ac.il (LD)

Abstract

Freshwaters are a very valuable resource in arid areas, such as Mediterranean countries. Freshwater systems are vulnerable ecological habitats, significantly disturbed globally and especially in arid areas. The Sea of Galilee is the largest surface freshwater body in the Middle East. It is an isolated habitat supporting unique fish populations, including endemic species and populations on the edge of their distribution range. Using the Sea of Galilee for water supply, fishing and recreation has been placing pressure on these fish populations. Therefore, efficient monitoring and effective actions can make a difference in the conservation of these unique fish populations. To set a baseline and develop molecular tools to do so, in this study, DNA barcoding was used to establish a database of molecular species identification based on sequences of *Cytochrome C Oxidase subunit I* gene. DNA barcodes for 22 species were obtained and deposited in Barcode of Life Database. Among these, 12 barcodes for 10 species were new to the database and different from those already there. Barcode sequences were queried against the database and similar barcodes from the same and closely related species were obtained. Disagreements between morphological and molecular species identification were identified for five species, which were further studied by phylogenetic and genetic distances analyses. These analyses suggested the Sea of Galilee contained hybrid fish of some species and other species for which the species definition should be reconsidered. Notably, the cyprinid fish defined as *Garra rufa*, should probably be considered as *Garra jordanica*. Taken together, along with data supporting reconsideration of species definition, this study sets the basis for further using molecular tools for monitoring fish

populations, understanding their ecology, and effectively managing their conservation in this unique and important habitat and in the region.

Introduction

Despite freshwater habitats only comprising of 1% of the Earth's surface water, they support 43% (13,000 species) of the world's fish species [1, 2], rich in biodiversity across all trophic levels [3, 4]. As a result of humans long standing dependence on water, either for direct uses like irrigation and drinking or indirect uses like hydro-electrical energy production and food fishing, many freshwater fish species have become highly endangered through disturbance and diminishing of natural habitats [5-9]. Further human derived pressure on natural fish diversity has also been documented due to introduction and invasion of exotic fish species [10-13].

Assessment of freshwater fish biodiversity and population status for effective management is therefore a priority, however, can be considered a great challenge [1, 6, 14, 15]. Conservation measures intended to mitigate the impact of human derived pressures have largely been slow and inadequate, and as a result, populations of many freshwater species have been declining rapidly [5, 6, 8, 15].

Due to its arid climatic conditions, Israel have been facing growing demands for freshwater. Consequently, its limited freshwater habitats have been under great human pressure. The Sea of Galilee (Lake Kinneret) is the biggest surface freshwater reservoir in the middle east (ca. 170 km^2), and due to its crucial role in water supply, it is highly regulated [16-18]. In terms of freshwater fish, about 18 native and eight non-native species were documented in the Sea of Galilee [19]. Native species are dominated by

cyprinids and cichlids that are of mainly Asian (Mesopotamian) and African origins, respectively [16]. The endemic cyprinid Kinneret bleak, *Mirogrex terraesanctae* (Steinitz, 1952), is probably the most abundant species in terms of both numbers and biomass [20]. Based on fishery landings, the native cichlid, Galilee tilapia, or St. Peter's fish *Sarotherodon galilaeus* (Linnaeus, 1758), is abundant, most valuable and thus, sought after. While *M. terraesanctae* is endemic, *S. galilaeus* is widespread in central and western Africa [8]. Non-native species, which were unintentionally introduced, include the European eel *Anguilla anguilla* (Linnaeus, 1759) that hitchhiked with mullet fry caught in coastal estuaries and stocked into the Sea of Galilee [21], *Xiphophorus hellerii* (Heckel, 1848) [22] and *Cichla kelberi* Kullander and Ferreira, 2006, probably released into the Sea of Galilee by hobbyists [23]. The non-native invasive mosquito fish *Gambusia affinis* (Baird and Girard, 1853) was intentionally introduced to control mosquitoes [16]. Additionally, significant non-native species were introduced to enhance commercial fishing, including common carp *Cyprinus carpio* Linnaeus, 1758, silver carp *Hypophthalmichthys molitrix* (Valenciennes, 1844), thinlip grey mullet *Chelon ramada* (Risso, 1827) and flathead grey mullet *Mugil cephalus* Linnaeus, 1758 [16, 22].

The dynamics of pelagic fish populations in Sea of Galilee is monitored mainly by means of acoustic surveys that measure abundance and distribution of fish shoals and estimate size of fish based on the echo target strength [24]. These acoustic surveys do not distinguish between species, but some inferences can be made based on estimated size of individuals and shoals. Based on these surveys, some estimates for population size of main pelagic species like the *M. terraesanctae* can be obtained [19]. Other inference on the population size comes from the commercial landing of sized fish [20, 25].

In recent years, DNA polymorphism, a more reliable and accurate method, is increasingly used for taxonomic and ecological research (e.g. [26-28]). DNA sequences of specific genes are used successfully to identify biological species in a method called DNA barcoding [29-31]. As certain DNA sequences in genomes evolve at rates similar to evolution of new species, DNA barcoding can complement and enhance species identification. DNA barcoding based methods can also be utilized for efficient monitoring of freshwater fish biodiversity, as DNA can easily be extracted from various types of samples including from environmental water samples, known as E-barcoding [32, 33]. Barcode sequences obtained from unidentified samples can be matched to available records from known samples for species identification. Thus, the application of DNA barcoding for reliable molecular identification of species, requires a reference database that connects DNA sequences to specific species [34, 35]. The Barcode of life Data systems (BOLD) is a comprehensive, parameterized DNA barcode reference library [36], created to support the use of barcode data and molecular species identification for studying various aspects of biodiversity.

Here, we applied DNA barcoding to samples taken mainly from the Sea of Galilee to create a reference barcoding database, within the platform of BOLD, for molecular identification of fish in the context of the Sea of Galilee and the region, an important tool for management and conservation of fish populations.

Methods

Sampling of fish species

To genetically barcode fish species from the Sea of Galilee and surrounding areas, an extensive sampling scheme was carried out to obtain samples from each of the species that could be found in the area. The Sea of Galilee, the main sampling site, and surrounding sites such as Asi stream (one of Beit She'an streams, south to the Sea of Galilee and part of the eastern watershed of Israel), Ein Afek pools (close by Acre, i.e. Akko, part of the western watershed of Israel) and Ein Te'o spring (close by Hula Lake, northern to Sea of Galilee and part of the eastern watershed of Israel) (see figure 1A by [27]), were sampled throughout 2013, 2014 and 2015 to collect samples of species that predominantly occur in this region of Israel by means of seine netting. Number of individual samples collected for each site and species are recorded in Table 1. Fish were anesthetized with 2-phenoxy-ethanol and after taxonomically identified to the species level in the field, a fin clip was taken from every fish. The fins were stored in labeled 2 mL Eppendorf tubes in 100% ethanol at -20°C. In most cases, after identification and sampling, fish were recovered from anesthesia and returned to their environment. Some fish were also sacrificed, photographed, preserved in formalin and deposited in the National Natural History Collections of the Hebrew University of Jerusalem, Israel with a museum number (Table 1).

Table 1. Fish collected, barcoded and deposited by species and location.

Species	Sampling location	N samples collected	N Samples barcoded	N Specimens deposited	Museum numbers at NNHC-HUJ
Clarias gariepinus	Sea of Galilee	8	5	0	
Mirogrex terraesanctae	Sea of Galilee	10	8	0	
Luciobarbus longiceps	Sea of Galilee	10	7	0	
Acanthobrama telavivensis	Ein Afek	13	7	3	HUJ21008
Capoeta damascina	Sea of Galilee	5	5	0	
Cyprinus carpio carpio	Sea of Galilee	14	5	0	

Garra rufa	Sea of Galilee	8	8	3	HUJ21009
	Beit She'an streams	4	3	0	
	Ein T'eo	6	3	0	
Garra nana	Sea of Galilee	2	2	0	
Carasobarbus canis	Sea of Galilee	9	6	0	
	Ein T'eo	5	5	0	
Hypophthalmichthys molitrix	Sea of Galilee	23	23	0	
Hypophthalmichthys molitrix Suspected as hybrids with *Hypophthalmichthys nobilis*	Sea of Galilee	6	6	0	
Pseudophoxinus kervillei	Sea of Galilee	1	1	0	
Oxynoemacheilus leontinae	Sea of Galilee	5	3	0	
Gambusia affinis	Sea of Galilee	7	3	0	
Aphanius mento	Sea of Galilee	1	1	0	
Salaria fluviatilis	Sea of Galilee	10	8	0	
Mugil cephalus	Sea of Galilee	10	7	0	
Chelon ramada	Sea of Galilee	13	9	0	
Astatotilapia flaviijosephi	Beit She'an streams	10	8	3	HUJ21007
Coptodon zillii	Sea of Galilee	29	8	2	HUJ21010
Tristramella simonis	Sea of Galilee	14	6	0	
Sarotherodon galilaeus	Sea of Galilee	120	17	9	HUJ21012
	Ein Afek	19	4	0	
Oreochromis aureus	Sea of Galilee	11	7	2	HUJ21011
Oreochromis aureus Suspected as hybrids with *Oreochromis niloticus*	Sea of Galilee	1	1	0	

Given are numbers of collected, DNA barcoded and museum deposited samples, as well as museum numbers for specimens deposited at the National Natural History Collections in the Hebrew University (NNHC-HUJ).

DNA extraction, polymerase chain reaction (PCR), purification and sequencing

DNA from stored fin samples was extracted using a modified protein salting-out method [37]. DNA concentration and quality (Optical Density OD_{260}/OD_{280} ratio) were measured using NanoDrop ND-1000 (NanoDrop Technologies) and visually examined by 1.5% TBE Agarose gel electrophoresis. DNA samples were stored at −20°C until used for further analysis.

A partial fragment of approximately 700bp of the *Cytochrome C oxidase subunit I* (COI), a mitochondrially encoded gene, was amplified by PCR using primers adopted from [31] (FishF1: 5’-TCAACCACCCACAAAGACATTGGCAC-3’ and FishR1: 5’-TAGACTTCTGGGTGGCCAAAGAATCA-3’). As DNA template, 50 ng of purified DNA were used in a total reaction volume of 20 µL. PCR mix contained: 2 µL 10× PCR buffer, $MgCl_2$ (25mM), dNTPs (6.6mM of each), Taq polymerase (1.5 units), primer mix (forward + reverse, 2.5 µM of each) and 10 µL water to complete the volume. PCR thermal protocol included an initial step of 94 °C for 2 min; followed by a touchdown profile of 94 °C for 30 s, annealing from 60 to 53 °C for 1 min with a decrease of 0.5 °C per each of 14 cycles, and extension at 72 °C for 2 min; further 23 cycles at the lower annealing temperature and final elongation step at 72 °C for 10 min. PCR products were verified by electrophoresis using 1.5% TBE Agarose gel containing ethidium bromide. PCR products were visualized using Gel Doc XR+ (BIO-RAD) and using ImageLab software (BIO-RAD). Successful PCR products were purified using an Exonuclease I-Shrimp Alkaline Phosphatase (ExoSAP-IT) method (USB, Cleveland, OH) and Sanger sequenced using BigDye V3.1 (Applied Biosystems) terminator chemistry in the forward and reverse directions on an ABI PRISM 3730xl DNA Analyzer (Applied Biosystems) following the manufacturers protocol.

Data analysis

COI sequence files were assembled and aligned using clustalW algorithm with BioEdit software (Version 7.2.5) [38]. All the polymorphisms detected by the software were manually inspected on the chromatograms and dubious cases were manually removed.

Successful sequences for each species together with their collection data were deposited into Barcode Of Life Data system (BOLD, https://www.boldsystems.org) [36] under project name LKCOX. See number of deposited sequences for the different species in Table 1. All sequences were aligned, manually inspected, and polymorphic sequences from the same species were defined as different haplotypes. Each haplotype for each species was queried against species level barcode records using BOLD identification module to derive molecular species identification. In some cases, the molecular identification did not completely match the original morphological species identification. For species where morphological and barcoding identification matched, only the barcodes of sampled fish were considered. For species with disagreements, also barcodes of the same and related species were downloaded from BOLD and considered in further sequence-based analyses. From the aligned sequences, an estimated unrooted gene tree, based on 530 bp long COI haplotypes, was constructed utilizing the Molecular Evolutionary Genetics Analysis (MEGA6) freeware [39], using the Kimura-2-parameter model, and the neighbor-joining tree construction method with 1000 bootstrap replicates [40]. The tree with the strongest bootstrap support was chosen for representation. Based on all COI haplotypes and using MEGA6 software, K2P genetic distance between any pair of haplotypes was calculated. The average pairwise differences were manually calculated by summing up and averaging the pairwise haplotype distances within and between different taxa. Fish that were suspected to be hybrids according to their morphology were excluded from the average distance calculations.

Ethical approval

Fish sampling from protected nature reserves was done under special permits 2012/38733 and 2014/40233 for sampling protected wildlife as reviewed and approved by the Israel Nature and National Parks Protection Authority. Some samples were obtained from catches of a commercial fishing vessel.

Results

Molecular barcoding

Samples and barcode sequences were obtained for a total of 22 fish species, including16 out of the 18 native and five non-native species, reported in the Sea of Galilee [16, 19, 20]. In addition, *Acanthobrama telavivensis* Goren, Fishelson & Trewavas, 1973, a species not reported in the lake, was sampled outside the Sea of Galilee for comparison to the closely related *Acanthobrama lissneri* Tortonese, 1952 [41], which is one of the native fish species that was not captured in any of the Sea of Galilee samplings. Another native fish species absent from this study, *Tristramella sacra* (Gunther, 1865), is considered to be extinct [42]. Missing from the non-native species were *Anguilla anguilla* and *Xiphophorus hellerii* that were not captured in any of the Sea of Galilee samplings. Altogether, COI sequences were analyzed from 176 individual fish, belonging to 22 freshwater species (Table 2). Additionally, seven sequences were analyzed from two fish species, *H. molitrix* (six sequences) and *Oreochromis aureus* (Steindachner, 1864) (one sequence), that were suspected as hybrids with aquaculture fish (hereafter referred to as ‘suspected hybrids’) (Table 2).

For 20 out of 22 species more than one specimen was collected and sequenced, and out of those, 12 species had more than one barcode sequence variant (COI haplotypes). The K2P distances within species that were calculated for each species ranged between 0 – 0.0086 with a mean of 0.0017 (Table 2). For ten species with barcodes existing in BOLD, 12 new barcodes were added. These added haplotypes were always those with the lower frequency in our data (Table 2).

Table 2. Summary of fish species, number of barcoded samples, COI haplotypes naming, haplotype frequencies and within species genetic distances.

Order	Family	Species	N barcoded samples	Haplotype	Haplotype frequency	K2P distance within species
Siluriformes	Clariidae	*Clarias gariepinus*	5	CgA	1	0
Cypriniformes	Cyprinidae	*Mirogrex terraesanctae*	8	AtA	0.625	0.0019
				AtB*	0.125	
				AtC	0.125	
				AtD	0.125	
		Luciobarbus longiceps	7	BlA	0.571	0.0019
				BlB	0.429	
		Acanthobrama telavivensis+	7	AtlA	0.571	0.0019
				AtlB*	0.429	
		Capoeta damascina	5	CdA	0.4	0.0038
				CdB	0.4	
				CdC*	0.2	
		Cyprinus carpio carpio	5	CcA	1	0
		Garra rufa	14	GrA	0.571	0.0086
				GrB*	0.214	
				GrC	0.143	
				GrD*	0.072	
		Garra nana	2	GnA	1	0
		Carasobarbus canis	11	BcA	0.909	0.0019
				BcB*	0.091	
		Hypophthalmichthys molitrix	23	HmA	0.783	0.0057
				HmB	0.217	
		*Hypophthalmichthys molitrix***	6	HmA	0.333	0.0362
				HmB	0.5	
				HmC	0.167	
		Pseudophoxinus kervillei	1	PkA*	1	0
	Nemacheilidae	*Oxynoemacheilus leontinae*	3	NlA*	0.667	0.0019
				NlB*	0.333	
Cyprinodontiformes	Poeciliidae	*Gambusia affinis*	3	GaA	1	0
	Cyprinodontidae	*Aphanius mento*	1	AmA*	1	0
Perciformes	Blenniidae	*Salaria fluviatilis*	8	SfA	0.875	0.0038

				SfB*	0.125	
Mugiliformes	Mugilidae	*Mugil cephalus*	7	McA	1	0
		Chelon ramada	9	LrA	0.889	0.0019
				LrB	0.111	
Cichliformes	Cichlidae	*Astatotilapia flaviijosephi*	8	AfA	0.75	0.0025
				AfB*	0.125	
				AfC	0.125	
		Coptodon zillii	8	TzA	1	0
		Tristramella simonis	6	TsA	0.833	0.0019
				TsB	0.167	
		Sarotherodon galilaeus	21	SgA	1	0
		Oreochromis aureus	7	OaA	1	0
		*Oreochromis aureus****	1	OaB	1	0

Here the species of fish is based on morphological taxonomy.
+ Not known to be found in the Sea of Galilee, closely related to *Acanthobrama lissneri*.
* Newly deposited haplotypes
** Suspected hybrid of *Hypophthalmichthys molitrix* and *Hypophthalmichthys nobilis*
*** Suspected hybrid of *Oreochromis niloticus* and *Oreochromis aureus*

For 19 out of 22 species, the molecular identification agreed with the original morphological identification, including for species with more than one COI haplotype.

Disagreements with molecular barcoding

Three species had disagreements between morphological and molecular barcoding identification. Further investigation was carried out adding all available COI barcode records from BOLD of query and related species. For the species which was not reported in the Sea of Galilee, *Acanthobrama telavivensis*, fish sampled from Ein Afek spring pools had two very similar haplotypes (99.8% sequence identity), with similar frequencies, one of which (AtlB) was new to BOLD. Five records were available from BOLD, four listed for *A. lissneri* and one for *A. telavivensis*, but all sharing an identical sequence. Thus, *A. telavivensis* fish with the haplotype common to our data and BOLD (AtlA), were identified by BOLD with 100% fit for both *A. lissneri* (a species native to the Sea of Galilee) and *A. telavivensis* (not reported in the Sea of Galilee).

Three *Gambusia affinis* collected samples, all with the same haplotype (GaA), were identified by BOLD as *Gambusia holbrooki* Girard, 1859 (100% fit), followed by *G. affinis* (99.81% fit). In the analysis of all available *G. holbrooki* and *G. affinis* records (BOLD and GaA), the GaA haplotype of sampled fish clustered together with *G. holbrooki* records of fish from widely distributed places.

Garra rufa was represented by 14 samples in our collection, showing four haplotypes, of which two were new to BOLD. BOLD identified *G. rufa* samples of this study as *Garra jordanica* Hamidan, Geiger & Freyhof, 2014, with 98.7-100% sequence identity for different haplotypes. This disagreement will be analyzed further later under its own subsection.

Molecular identification using BOLD for samples of fish suspected as hybrids provided genetic evidence to support hybridization. A sample suspected as *Oreochromis niloticus* (Linnaeus, 1758) x *Oreochromis aureus* hybrid, which is neither native nor a hybrid stocked into the Sea of Galilee, had a COI sequence with a perfect match (100%) to *O. niloticus*, indicating that this fish is at least partly hybridized with *O. niloticus*, a non-native species not reported to inhabit the Sea of Galilee. Additionally, in samples from the Sea of Galilee that according to their morphology were suspected to be *Hypophthalmichthys molitrix* hybridized with *Hypophthalmichthys nobilis* (Richardson, 1845), three different COI haplotypes were found. Two of them matching BOLD *H. molitrix*, reported to be stocked into the lake, while the third matching BOLD *H. nobilis* (100%), not reported in the lake, supporting possible hybridizations in both directions. K2P distance within these hybrid fish was 0.0362, over 4-fold higher than the maximum distance found within the other species, supporting that these fish were hybrids.

Genetic distances and phylogeny from barcoding data

To evaluate the DNA barcoding efficiency in distinguishing among species, K2P genetic distances between each pair of species was calculated. The genetic distance between species ranged between 0.0095 – 0.2886, with a mean distance of 0.2295, over 100-fold higher compared to the mean within species K2P distance (0.0017). Additionally, the range of between-species distances did not overlap with that of within-species, supporting the utility of COI haplotypes in differentiation of these species. The species differentiation is evident also from the phylogenetic tree based on the COI haplotypes (Fig 1). In the tree, species were grouped similarly to the expected tree based on the NCBI taxonomy browser (https://www.ncbi.nlm.nih.gov/Taxonomy/taxonomyhome.html/). The phylogeny separated well all the different species, while grouping together multiple haplotypes of the same species. Furthermore, on the higher taxonomic levels, the phylogeny separated well the three most represented families in the Sea of Galilee, cyprinids, cichlids and mugilids.

Fig 1. A neighbor-joining unrooted tree of species based on COI haplotypes. Next to branch edges are haplotype acronyms as listed in Table 2. Numbers next to bifurcations denote percent bootstrap support. For some species, more than one haplotype was identified (denoted A, B etc.). For major families, which are represented by multiple species, a mean K2P between species distance matrix is given to the right. (*) – HmC haplotype derived from specimens suspected as *Hypophthalmichthys molitrix* x *Hypophthalmichthys nobilis* hybrids, matches *H. nobilis* sequences from BOLD. (**) - OaB haplotype derived from a specimen suspected as *Oreochromis niloticus* x *Oreochromis aureus* hybrid, matches *O. niloticus* sequences from BOLD.

However, a few discrepancies were found also in the genetic distances and phylogeny data. A genetic distance of 0.08 was found between barcodes of *Acanthobrama lisnneri/Acanthobrama telavivensis* and *Mirogrex terraesanctae*, which was a distance relatively small for between-genera and similar to the within-genera distance found between *Garra rufa* and *Garra nana* (Heckel, 1843).

Additionally, within the *Cichlidae* clade in the neighbor-joining phylogenetic tree (Fig. 1), *Oreochromis aureus* (Haplotype OaA) clustered together with species from a different genus, *S. galilaeus* (haplotype SgA), rather than with the species with which its genus is shared, *O. niloticus* (haplotype OaB). Support for this genetic similarity comes from a smaller K2P distance between *O. aureus* and *S. galilaeus* (0.0095) compared to distances between genera of other Sea of Galilee cichlids (0.06-0.16), and genera of cyprinids (0.08-0.23) (Fig. 1).

Species in the *Garra* genus

Comparison of sampled *Garra rufa* fish haplotypes to BOLD database accessions gave inconsistencies in species definition. Also, K2P distance within haplotypes of fish from Israel, identified as *G. rufa* in this study, was 0.0086, the highest value found within species groups and more than four times higher than the mean within-species value (0.0014) of all other species groups excluding *G. rufa* haplotypes. *Garra rufa* fish were collected in this study from three places: Sea of Galilee, Asi stream (one of Beit She'an streams) and Ein Te'o (North of the Sea of Galilee) (Fig 2A). These fish had four COI haplotypes (GrA – GrD) that were unevenly distributed between collection sites. Among Sea of Galilee samples, five, two and one fish had haplotypes 'GrA', 'GrC' and 'GrD',

respectively. All three fish from Ein Te'o had haplotype 'GrA' and all three fish from Asi stream had haplotype 'GrB' (Fig 2A).

Fig 2. Sampling sites and clustering of *Garra* samples based on COI haplotypes. Acronyms of populations are: GrA – *Garra rufa* from haplotype A; GrB – *Garra rufa* from haplotype B; GrC – *Garra rufa* from haplotype C; GrD – *Garra rufa* from haplotype D; Gg – *Garra ghorensis* from the southern basin of the Dead sea; Gj – *Garra Jordanica* from the northern basin of the Dead sea; GnA – *Garra nana* from haplotype A; GnB – *Garra nana* from haplotype B. **(A)** Map showing the sampling sites in a regional context. Different *Garra* haplotypes were denoted by differently colored circles in each sampling site on the regional map. Map was reprinted from DMY + NordNordWest (https://commons.wikimedia.org/wiki/File:Israel_by_israeli_law_adm_location_map.svg), under a CC BY-SA 4.0 license, original copyright 2017. **(B)** A neighbor-joining unrooted tree constructed for haplotypes obtained from samples collected in this study alongside haplotypes mined from BOLD. Next to the edges are haplotype acronyms as indicated above and next to bifurcations are percent bootstrap support values. Haplotype text colors in the tree match circle colors in the map (A).

Sequences of 16 *Garra* species samples from this study together with 33 BOLD accessions, which had geographic location information assigned to them (see Table S1), were aligned to construct a neighbor-joining phylogenetic tree (Fig 2B). Haplotypes of *G. nana* were separated from all other haplotypes defined as *G. rufa, G. jordanica* and *Garra ghorensis* Krupp, 1982. The two haplotypes of *G. nana* (GnA and GnB) were relatively distinct from one another (K2P = 0.0381; Table 3). GnA, was found in fish from the Sea of Galilee and the more northern location in Syria, while GnB, was found in fish from an isolated location more southern in Syria (Fig 2A).

Table 3. Genetic distances between geographically separate *Garra* populations.

	Gr North	Gr Asi	Gj	Gg	Gr Asia	GnA	GnB
Gr North	0.0025<br>0 - 0.0037						

Gr Asi	0.0143<br>0.013-0.0168	0					
Gj	0.0031<br>0.0018-0.0055	0.0149	0				
Gg	0.0414<br>0.0401-0.0441	0.0381	0.0421	0			
Gr Asia	0.0462<br>0.0422-0.0523	0.0469<br>0.0442-0.0502	0.0469<br>0.0442-0.0502	0.0587<br>0.0563-0.0604	0.0065<br>0.0018-0.013		
GnA	0.0792<br>0.0792-0.0792	0.0857	0.0814	0.0834	0.0635<br>0.0582-0.0685	0	
GnB	0.0899<br>0.0899-0.0899	0.0965	0.0921	0.0854	0.0692<br>0.0664-0.727	0.0381	0

In each table cell, top row is the mean K2P value and bottom row is the range of pairwise distances. Acronyms for populations are: GrNorth – *Garra rufa* from Sea of Galilee and Ein Te'o of Northern Jordan valley; GrAsi – *Garra rufa* from Asi atream of Beit She'an valley; Gj – *Garra jordanica* from the northern basin of the Dead Sea; Gg – *Garra ghorensis* from the southern basin of the Dead Sea; GrAsia – *Garra rufa* from Asian countries including Turkey, Iran and Iraq; GnA – *Garra nana* from Haplotype A; GnB – *Garra nana* from Haplotype B.

Israel *G. rufa* samples from the Sea of Galilee and Ein Te'o spring had haplotypes GrA, GrC and GrD. These haplotypes of *G. rufa* fish from Israel clustered with BOLD haplotype Gj of *G. jordanica* fish sampled from streams in the northern basin of the Dead Sea in Jordan (Fig 2B). Close by in the phylogeny, yet separated, was haplotype GrB of *G. rufa* fish from Asi stream, again similar to *G. jordanica* haplotype, but somewhat genetically separated from haplotypes of the Sea of Galilee population. Further separated from this cluster of haplotypes (Israeli *G. rufa* and non-Israeli *G. jordanica*) was the BOLD *G. ghorensis* haplotype (Gg) of fish from streams in the southern basin of the Dead Sea. Further away, clustered together were BOLD haplotypes (GrE – GrN) of fish defined as *G. rufa* from other Asian countries (GrAsia - Turkey, Iran and Iraq).

Given the genetic divergence found for geographically isolated fish, pairwise K2P distances were calculated for *Garra* and compared to the distances found within and

between species based on all species (Table 2, Fig 1). For each *Garra* combination of species and geographic location the mean, minimum and maximum values of the pairwise K2P distances were calculated (Table 3). It is evident that the genetic separation between populations of *G. rufa* increases with geographic distance. The Israeli *G. rufa* fish, are genetically divergent from *G. rufa* fish from farther eastern and northern Asian countries (Mean K2P between Gr North and Gr Asia = 0.0462, and between Gr Asi and Gr Asia = 0.0469; Table 3). The mean K2P value between these *G. rufa* clades is considerably higher than what was observed as within-species differences (Table 2), and more similar to between-species ones (Fig 1). On the other hand, mean K2P distance between *G. jordanica* BOLD haplotype and Israeli *G. rufa* fish (Gr North; Fig 2) was very low (0.0031, Table 3) and well within the range of within-species distances (Table 2).

Among *G. rufa* fish from Israel, Asi stream fish of Beit she'an valley (Gr Asi) had a slightly different haplotype (GrB) than neighboring fish from Sea of Galilee and Ein T'eo spring (mean K2P between Gr Asi and Gr North = 0.0143) and thus, were also slightly genetically different from BOLD samples defined as *G. jordanica* (mean K2P between Gr Asi and Gj = 0.0149) (Table 3). These mean K2P distances are slightly higher than within-species distances (Table 2) and considerably lower than between-species differences (Fig 1).

Samples from BOLD of *G. ghorensis* fish from the southern part of the Jordan valley were similarly genetically distant from Asian samples of *G. rufa* (mean K2P between Gg and Gr Asia = 0.0587) and from Israeli *G. rufa* samples (mean K2P between Gg and Gr North = 0.0414 and between Gg and Gr Asi = 0.0381) (Table 3). Finally,

samples of *G. nana* were genetically distant from *G. rufa*, *G. jordanica* and *G. ghorensis* (Table 3), at levels similar to between-species distances (Fig 1). From all *Garra* populations, *G. nana* samples were the least distant from Asian *G. rufa* samples (Table 3).

Discussion

The development of molecular methods and their availability have been driving an effort to augment morphological taxonomy of species with molecular taxonomy based on DNA sequencing [29, 43, 44]. Since within fish there are species-rich and diverse taxonomic groups, DNA barcoding has great potential to improve the taxonomy within these groups [30, 45]. In a previous study conducted by [46], the cichlids of Israel were barcoded with success. In this study, focus was given to the fish of the Sea of Galilee and surrounding water bodies in the region, including species other than cichlids. Altogether, COI sequences from 176 individual fish, belonging to 22 morphologically-defined freshwater species, were analyzed. From this study, 12 new barcode haplotypes were added to the public database, belonging to 10 of the 16 native species captured. Thus, minor frequency new barcodes were identified for a high proportion of the native species, an indication for the genetic divergence and uniqueness of these regional native populations. Except for a few discrepancies which will be discussed later, for 19 out of the 22 species, morphological and DNA barcoding agreed on the species definition. In theory, the 22 species, as they were defined by morphological taxonomy, should have returned 22 DNA barcodes, but in practice, 40 different COI haplotypes were recovered. For 12 species, mostly belonging to the Cyprinidae and Cichlidae, two or more COI haplotypes were

found, indicating that genetic variation had evolved not exactly at the same rate as species did. Once more populations of these species from different locations will be studied, a comparison to this study could highlight if this intraspecific variation represents the beginning of population/species divergence or is this variation ancestral/common to the species.

The mean K2P genetic distance within species was small, corroborating within-species ranges in other studies [26, 29, 45, 47]. Furthermore, the range of genetic distances between species did not overlap the range within species. Therefore, importantly, for the Sea of Galilee, COI sequencing provides a reliable method to identify species and study their ecology.

Defining the expected K2P genetic distance (mean and range) for different haplotypes within and between species (or higher taxonomic levels), can provide a useful tool for ecological and taxonomic research [29, 30]. Here we used phylogeny, genetic distances and geographic information to study a few discrepancies identified between morphological taxonomy and DNA barcoding-based taxonomy. Two species of the same genus, *A. lissneri*, native to the Sea of galilee, and *A. telavivensis*, reported only outside the lake, shared one of the barcodes found for *A. telavivensis*. The other barcode of *A. telavivensis* was also very similar, suggesting high genetic similarity of all available barcodes and thus, possible reconsideration of these fish taxonomy. Additionally, a relatively small genetic distance, typical of distances between species, was found between *A. lissneri/telavivensis* and *M. terraesanctae*, two species of different genera, suggesting these genera are closer than expected. Thus, smaller than expected molecular divergence was found both between species of the same genera and between species of

different genera, all belonging to this family. It should be noted that the genus of *Mirogrex terraesanctae* was originally defined as *Acanthobrama terraesanctae* [41, 48] and the molecular data supports the original classification. Another species, the mosquito fish *G. affinis*, invasive to the Sea of Galilee, had an identical barcode to BOLD barcodes of *G. holbrooki* from various other locations. As these fish were introduced into several freshwater bodies to combat mosquito-borne diseases, it might be worthwhile to reconsider if indeed they deserve to be separate species and if they do, then the fish in the Sea of Galilee might be *G. holbrooki* rather than *G. affinis*.

Morphological taxonomy of African cichlids, a very species-rich group of fish, was occasionally revisited [49-51]. Cichlids in the Sea of Galilee are of ancient African origin and they constitute a key group in the lake, with both ecological and commercial significance. Here, phylogeny proximity and a small genetic distance, on the order of between species of the same genera, were found between *O. aureus* and *S. galilaeus*, two species from different genera. This level of molecular relatedness was considerably smaller compared to the distance between *O. aureus* and *O. niloticus*, which share the same genus. Thus, such and other molecular methods can help further revisiting and updating cichlid phylogeny [50]. Furthermore, in this study a fish morphologically suspected as a hybrid between *O. niloticus* and *O. aureus* was molecularly identified as *O. niloticus*, supporting hybrids might exist in the lake [22], since pure *O. niloticus* are considered too cold-temperature sensitive to become established there. Also, indication for the presence of hybrid fish between *H. molitrix* and *H. nobilis* were found. Both these specific cichlid and carp hybrids are commonly used in Israeli aquaculture and as such,

fish might have somehow reached into the Sea of Galilee by unintentional introduction or as escapees from nearby ponds.

Phylogeny, genetic distances and geographic information were mainly employed for studying the discrepancies found in regional *Garra* taxonomy, for which COI sequences of samples collected in this study were analyzed together with sequences from the public BOLD database. Presence of *G. rufa* and *G. nana* has been reported in the upper Jordan valley system (including in the Sea of Galilee, Hula valley and Beit She'an valley streams) [16, 19]. *Garra jordanica* and *Garra ghorensis* were reported as endemic to freshwater springs and streams northern and southern to the Dead Sea, respectively [52, 53]. The phylogenetic tree based on COI sequences suggested some level of geographic separation between groups of haplotypes.

The genetic divergence found between *G. rufa* from the Asi stream (Beit She'an valley) south to the Sea of Galilee and the more northern Israeli fish (those from the Sea of Galilee itself and from the Hula valley) was the highest of within-species distances. This level of genetic divergence within the northern Israeli fish, is likely due to the recent (~24,000 years ago) isolation of the Beit She'an valley water system from the Jordan River system [54].

Furthermore, *G. rufa* from Turkey, Iran and Iraq formed a cluster separated from Mediterranean *Garra* samples. The range of K2P distances (0.0422-0.0523) suggests that Asian *G. rufa* and Mediterranean *G. rufa* should be considered as different species. A recent study suggested that fish described as *G. rufa* from the springs and streams northern to the Dead Sea in Jordan, are in fact a separate species described as *G. jordanica* [52]. As found here, Israeli fish defined as *G. rufa* are genetically more similar

to BOLD *G. jordanica* than to *G. rufa* from Asian countries. K2P distances between Israeli *G. rufa* haplotypes and BOLD *G. jordanica* haplotype ranged between 0.0018-0.0149, falling well inside the within-species range. Additionally, in the phylogentic tree, *G. jordanica* haplotype clustered together with haplotypes of *G. rufa* from Israel, consistent also with their geographic proximity. Thus, given the distance from Asian *G. rufa* and proximity to *G. jordanica*, a change in species definition for *G. rufa* fish from Israel, from *G. rufa* to *G. jordanica*, should be considered. This was also suggested theoretically by [52] based on the geographic distribution data, and is now supported by DNA barcoding evidence. The analyses of discrepancies between morphological and molecular species definition, primarily that of the *Garra* complex, are examples for how DNA barcoding can assist in resolving and revisiting taxonomic and ecological questions.

The utility of species identification using DNA barcoding in the context of ecological research was extensively discussed in the last decade and new implementations of DNA barcoding are being published ever since this method was standardized [28, 44, 55, 56]. The sampling scheme employed for this study allowed for the first time to develop and apply molecular genetics tools to many of the freshwater fish species of Israel in the context of their natural habitats. Studies are starting to employ molecular tools relying on or assisted by molecular species identification to address ecological questions regarding native fish populations in the Sea of Galilee, such as the spatial and temporal distribution and abundance of cichlid fry along lake shores [57] and the decline of genetic variation in the *S. galilaeus* lake population [27].

Conclusions

This study established a molecular DNA barcoding database for the ecologically isolated native and non-native fish species inhabiting the Sea of Galilee. The data obtained here exemplified how in combination with the public BOLD database, analyses of geographic patterns, morphological and molecular taxonomy can address issues in species definition on one hand, and on the other, how the molecular data could be used as a basis to address ecological questions concerning freshwater fish assemblages. Developing modern, molecular tools for identification of fish down to species level and even further, to population level, are being valuable tools for monitoring fish populations and supporting management and conservation decisions of significant populations in key freshwater bodies as the Sea of Galilee.

Acknowledgments

The authors wish to thank all who assisted in sampling of fish: David Cummings (Kinneret Limnological Laboratory, IOLR), Dr. Noam Leader, Dr. Dana Milstein, Dr. Amit Dolev and Gisele Hazzan (Israel National Parks Authority), Menachem Lev (Kibbutz Ein Gev fishing vessel) and colleagues from the laboratory and the Animal Sciences Department. LD is chair of the Vigevani Senior Lectureship in Animal Sciences.

Supporting information

S1 table. *Garra* species accessions used for analyses. Garra species accessions and geographic information for 16 sample sequences from this study and 33 BOLD accessions.

References

1. Levêque C, Oberdorff T, Paugy D, Stiassny M, Tedesco PA. Global diversity of fish (Pisces) in freshwater. Freshwater animal diversity assessment: Springer; 2007. p. 545-67.
2. Nelson J. Fishes of the world John Wiley and Sons. Inc, Hoboken, New Jersey. 2006.
3. Carpenter SR, Stanley EH, Vander Zanden MJ. State of the world's freshwater ecosystems: physical, chemical, and biological changes. Annual review of Environment and Resources. 2011;36:75-99.
4. Vörösmarty CJ, McIntyre PB, Gessner MO, Dudgeon D, Prusevich A, Green P, et al. Global threats to human water security and river biodiversity. nature. 2010;467(7315):555-61.
5. Darwall WR, Freyhof J. Lost fishes, who is counting? The extent of the threat to freshwater fish biodiversity. Conservation of freshwater fishes. 2016:1-36.
6. Dudgeon D, Arthington AH, Gessner MO, Kawabata Z-I, Knowler DJ, Lévêque C, et al. Freshwater biodiversity: importance, threats, status and conservation challenges. Biological reviews. 2006;81(2):163-82.
7. Dynesius M, Nilsson C. Fragmentation and flow regulation of river systems in the northern third of the world. Science. 1994;266(5186):753-62.
8. Lind CE, Brummett RE, Ponzoni RW. Exploitation and conservation of fish genetic resources in Africa: issues and priorities for aquaculture development and research. Reviews in Aquaculture. 2012;4(3):125-41.
9. Nilsson C, Reidy CA, Dynesius M, Revenga C. Fragmentation and flow regulation of the world's large river systems. Science. 2005;308(5720):405-8.
10. Casal CMV. Global documentation of fish introductions: the growing crisis and recommendations for action. Biological invasions. 2006;8(1):3-11.
11. Havel JE, Kovalenko KE, Thomaz SM, Amalfitano S, Kats LB. Aquatic invasive species: challenges for the future. Hydrobiologia. 2015;750(1):147-70.
12. Leprieur F, Beauchard O, Blanchet S, Oberdorff T, Brosse S. Fish invasions in the world's river systems: when natural processes are blurred by human activities. PLoS Biol. 2008;6(2):e28.
13. Thomaz SM, Kovalenko KE, Havel JE, Kats LB. Aquatic invasive species: general trends in the literature and introduction to the special issue. Hydrobiologia. 2015;746(1):1-12.
14. Reid AJ, Carlson AK, Creed IF, Eliason EJ, Gell PA, Johnson PT, et al. Emerging threats and persistent conservation challenges for freshwater biodiversity. Biological Reviews. 2019;94(3):849-73.
15. Sala OE, Chapin FS, Armesto JJ, Berlow E, Bloomfield J, Dirzo R, et al. Global biodiversity scenarios for the year 2100. science. 2000;287(5459):1770-4.

16. Goren M, Ortal R. Biogeography, diversity and conservation of the inland water fish communities in Israel. Biological Conservation. 1999;89(1):1-9.
17. Markel D, Shamir U, Green P. Operational management of Lake Kinneret and its watershed. Lake Kinneret: Springer; 2014. p. 541-60.
18. Serruya C. Lake kinneret: Springer Science & Business Media; 2012.
19. Ostrovsky I, Goren M, Shapiro J, Snovsky G, Rynskiy A. Fish biology and ecology. Lake Kinneret: Springer; 2014. p. 273-92.
20. Ostrovsky I, Zohary T, Shapiro J, Snovsky G, Markel D. Fisheries management. Lake Kinneret: Springer; 2014. p. 635-53.
21. Ben-Tuvia A. Man-induced changes in the freshwater fish fauna of Israel. Aquaculture Research. 1981;12(4):139-48.
22. Goren M, Galil B. A review of changes in the fish assemblages of Levantine inland and marine ecosystems following the introduction of non-native fishes. Journal of Applied Ichthyology. 2005;21(4):364-70.
23. Golani D, Sonin O, Snovsky G, David L, Tadmor-Levi R. The occurrence of the peacock bass (Cichla kelberi Kullander and Ferreira 2006) in Lake Kinneret (Sea of Galilee), Israel. BioInvasions Records. 2019;8(3):706-11.
24. Walline PD, Pisanty S, Lindem T. Acoustic assessment of the number of pelagic fish in Lake Kinneret, Israel. Hydrobiologia. 1992;231(3):153-63.
25. Kantoussan J, Ecoutin JM, Fontenelle G, de Morais LT, Laë R. Catch per Unit Effort and yields as indicators of exploited fish communities: application to two West A frican reservoirs. Lakes & Reservoirs: Research & Management. 2014;19(2):86-97.
26. Ali FS, Ismail M, Aly W. DNA barcoding to characterize biodiversity of freshwater fishes of Egypt. Molecular Biology Reports. 2020;47(8):5865-77.
27. Borovski T, Tadmor-Levi R, Shapiro J, Rubinstein G, Agyakwah SK, Hulata G, et al. Historical and recent reductions in genetic variation of the Sarotherodon galilaeus population in the Sea of Galilee. Conservation Genetics. 2018;19(6):1323-33.
28. Hughes JM, Finn DS, Monaghan MT, Schultheis A, Sweeney BW. Basic and applied uses of molecular approaches in freshwater ecology. Freshwater Science. 2014;33(1):168-71.
29. Hebert PD, Cywinska A, Ball SL, Dewaard JR. Biological identifications through DNA barcodes. Proceedings of the Royal Society of London Series B: Biological Sciences. 2003;270(1512):313-21.
30. Ward RD, Hanner R, Hebert PD. The campaign to DNA barcode all fishes, FISH-BOL. Journal of fish biology. 2009;74(2):329-56.
31. Ward RD, Zemlak TS, Innes BH, Last PR, Hebert PD. DNA barcoding Australia's fish species. Philosophical Transactions of the Royal Society B: Biological Sciences. 2005;360(1462):1847-57.
32. Pont D, Rocle M, Valentini A, Civade R, Jean P, Maire A, et al. Environmental DNA reveals quantitative patterns of fish biodiversity in large rivers despite its downstream transportation. Scientific reports. 2018;8(1):1-13.
33. Ushio M, Murakami H, Masuda R, Sado T, Miya M, Sakurai S, et al. Quantitative monitoring of multispecies fish environmental DNA using high-throughput sequencing. Metabarcoding and Metagenomics. 2018;2:e23297.
34. Ekrem T, Willassen E, Stur E. A comprehensive DNA sequence library is essential for identification with DNA barcodes. Molecular phylogenetics and evolution. 2007;43(2):530-42.
35. Weigand H, Beermann AJ, Čiampor F, Costa FO, Csabai Z, Duarte S, et al. DNA barcode reference libraries for the monitoring of aquatic biota in Europe: Gap-analysis and recommendations for future work. Science of the Total Environment. 2019;678:499-524.

36. Ratnasingham S, Hebert PD. BOLD: The Barcode of Life Data System (http://www.barcodinglife.org). Molecular ecology notes. 2007;7(3):355-64.
37. Lopera-Barrero NM, Povh JA, Ribeiro RP, Gomes PC, Jacometo CB, Lopes TS. Comparison of DNA extraction protocols of fish fin and larvae samples: modified salt (NaCl) extraction. Ciencia e Investigación Agraria. 2008;35(1):65-74.
38. Hall TA, editor BioEdit: a user-friendly biological sequence alignment editor and analysis program for Windows 95/98/NT. Nucleic acids symposium series; 1999: [London]: Information Retrieval Ltd., c1979-c2000.
39. Tamura K, Stecher G, Peterson D, Filipski A, Kumar S. MEGA6: molecular evolutionary genetics analysis version 6.0. Molecular biology and evolution. 2013;30(12):2725-9.
40. Felsenstein J. Confidence limits on phylogenies: an approach using the bootstrap. evolution. 1985;39(4):783-91.
41. Goren M. The cyprinid fishes of Acanthobrama Heckel and related genera. 1973.
42. Tristramella sacra. The IUCN Red List of Threatened Species [Internet]. 2014 [cited 02 December 2020].
43. Coleman CO, Radulovici AE. Challenges for the future of taxonomy: talents, databases and knowledge growth. Megataxa. 2020;1(1):28-34.
44. Hajibabaei M, Singer GA, Hebert PD, Hickey DA. DNA barcoding: how it complements taxonomy, molecular phylogenetics and population genetics. TRENDS in Genetics. 2007;23(4):167-72.
45. Hubert N, Hanner R, Holm E, Mandrak NE, Taylor E, Burridge M, et al. Identifying Canadian freshwater fishes through DNA barcodes. PLoS one. 2008;3(6):e2490.
46. Shirak A, Cohen-Zinder M, Barroso RM, Seroussi E, Ron M, Hulata G. DNA barcoding of Israeli indigenous and introduced cichlids. 2009.
47. Mojekwu TO, Cunningham MJ, Bills RI, Pretorius PC, Hoareau TB. Utility of DNA barcoding in native Oreochromis species. Journal of Fish Biology. 2021;98(2):498-506.
48. Steinitz H. XXXIII.—Acanthobrama terrae-sanctae, sp. n., from Lake Tiberias, Israel. Annals and Magazine of Natural History. 1952;5(51):293-8.
49. Dunz AR, Schliewen UK. Molecular phylogeny and revised classification of the haplotilapiine cichlid fishes formerly referred to as "Tilapia". Molecular Phylogenetics and Evolution. 2013;68(1):64-80.
50. Nyingi WD, Agnèse J-F, Ford AG, Day JJ, Ndiwa TC, Turner GF, et al. Identifying and conserving tilapiine cichlid species in the twenty-first century. The Behavior, Ecology and Evolution of Cichlid Fishes: Springer; 2021. p. 285-312.
51. Trewavas E. Tilapiine fishes of the genera Sarotherodon, Oreochromis and Danakilia. British Museum (Natural History), London. 1983.
52. Hamidan NA, Geiger MF, Freyhof J. Garra jordanica, a new species from the Dead Sea basin with remarks on the relationship of G. ghorensis, G. tibanica and G. rufa (Teleostei: Cyprinidae). Ichthyological Exploration of Freshwaters. 2014;25(3):223-36.
53. Hamidan NA, Mir S. The status of Garra ghorensis in Jordan: distribution, ecology and threats. Zoology in the Middle East. 2003;30(1):49-54.
54. Stein M. Late quaternary limnological history. Lake Kinneret: Springer; 2014. p. 39-58.
55. Andrew RL, Bernatchez L, Bonin A, Buerkle CA, Carstens BC, Emerson BC, et al. A road map for molecular ecology. Molecular ecology. 2013;22(10):2605-26.
56. Blanchet S. The use of molecular tools in invasion biology: an emphasis on freshwater ecosystems. Fisheries Management and Ecology. 2012;19(2):120-32.

57. Cummings D, Goren M, Gasith A, Zohary T. Inundated shore vegetation as habitat for cichlids breeding in a lake subjected to extreme water level fluctuations. Inland Waters. 2017;7(4):449-60.

Figure 1

Click here to access/download;Figure;Figure 1.tif

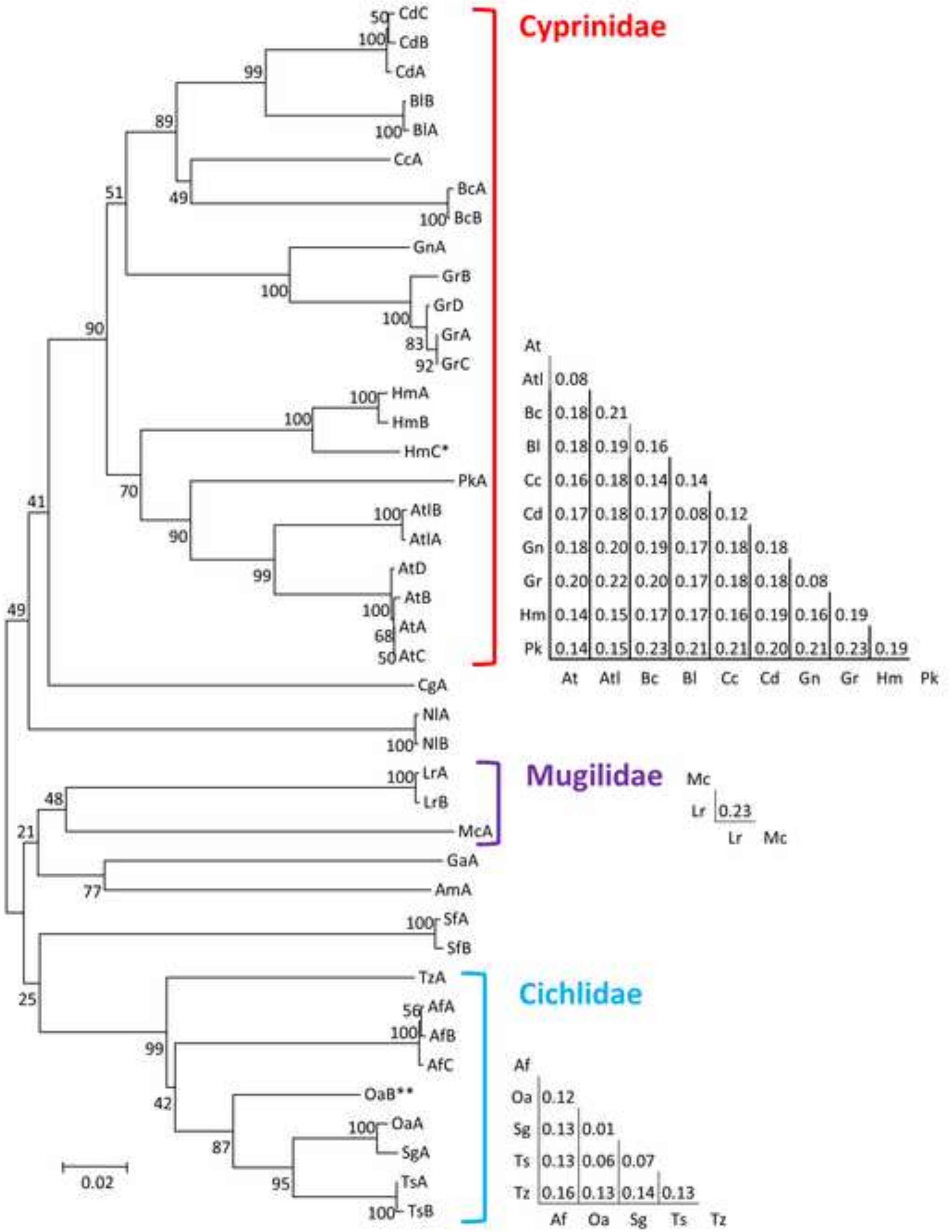

Figure 2

Click here to access/download;Figure;Figure 2.tif

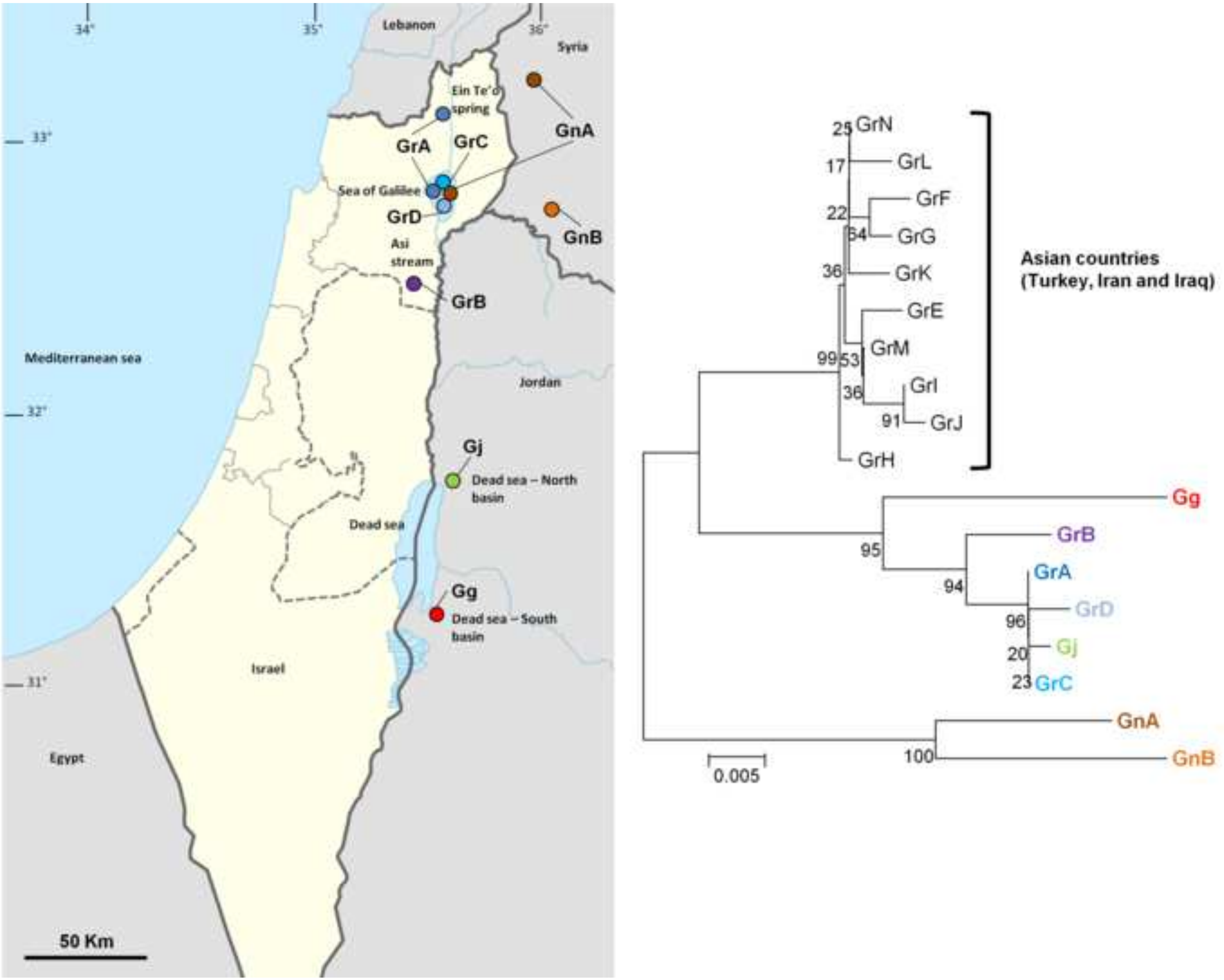

Click here to access/download
Supporting Information
S1_table.xlsx

Establishing and using a genetic database for resolving identification of fish species in the Sea of Galilee, Israel

Short title: DNA barcoding the fish species of the Sea of Galilee

Roni Tadmor-Levi[1,2], Tomer Borovski[2,†], Evgeniya Marcos-Hadad[2], James Shapiro[3], Gideon Hulata[4], Daniel Golani[1], Lior David[1,2,*].

[1] National Natural History Collections and Department of Ecology, Evolution and Behavior, The Hebrew University of Jerusalem, Jerusalem, Israel.

[2] Department of Animal Sciences, RH Smith Faculty of Agriculture Food and Environment, The Hebrew University of Jerusalem, Rehovot, Israel

[3] Fishery and Aquaculture Unit, Ministry of Agriculture and Rural Development, Beit-Dagan, Israel

[4] Department of Poultry and Aquaculture, Institute of Animal Science, Agricultural Research Organization (ARO), Volcani Center, Rishon LeZion, Israel

[†] Deceased

[*] Corresponding Author

E-mail: lior.david@mail.huji.ac.il (LD)

Abstract

Freshwaters are a very valuable resource in arid areas, such as Mediterranean countries. Freshwater systems are vulnerable ecological habitats, significantly disturbed globally and especially in arid areas. The Sea of Galilee is the largest surface freshwater body in the middle east. ~~The lake~~It is an isolated habitat supporting unique fish populations, including endemic species and populations on the edge of their distribution range. Using the ~~lake~~ Sea of Galilee for water supply, fishing and recreation has been placing pressure on these fish populations. Therefore, efficient monitoring and effective actions can make a difference in the conservation of these unique fish populations. To set a baseline and develop molecular tools to do so, in this study, DNA barcoding was used to establish a database of molecular species identification based on sequences of *Cytochrome C Oxidase subunit I* gene. DNA barcodes for 22 species were obtained and deposited in Barcode of Life Database. Among these, 12 barcodes for 10 species were new to the database and different from those already there. Barcode sequences were queried against the database and similar barcodes from the same and closely related species were obtained. Disagreements between morphological and molecular species identification were identified for ~~five~~ three species, which were further studied by phylogenetic and genetic distances analyses. These analyses suggested the ~~lake~~ Sea of Galilee contained hybrid fish of some species and other species for which the species definition should be reconsidered. Notably, the cyprinid fish defined as *Garra rufa*, should probably be considered as *Garra jordanica*. Taken together, along with data supporting reconsideration of species definition, this study sets the basis for further ~~developing~~ using molecular tools for ~~identification and~~ monitoring ~~unique~~ fish

populations, understanding their ecology and ~~for~~ effectively managing their conservation ~~of fish biodiversity~~ in this unique and important habitat and in the region.

Introduction

~~The ecological balance and sustainability of freshwater habitats are very vulnerable as they can be disturbed by many types of biotic and abiotic changes.~~ Despite freshwater habitats only comprising of 1% of the Earth's surface water, they support 43% (13,000 species) of the world's fish species (Levêque *et al.*, 2007; Nelson, 2006), rich in biodiversity across all trophic levels [Vörösmarty et al., 2010; Carpenter et al., 2011]. As a result of humans long standing dependence on water, either for direct uses like irrigation and drinking or indirect uses like hydro-electrical energy production and food fishing, many freshwater fish species have become highly endangered through disturbance and diminishing of natural habitats (Darwall, Freyhof, 2016; Dudgeon *et al.*, 2006; Dynesius, Nilsson, 1994; Lind *et al.*, 2012; Nilsson *et al.*, 2005). ~~A long standing dependence of humans on water, whether for direct uses like irrigation and drinking or for indirect uses like hydro-electrical energy production and food fishing, contributed considerably to the poor ecological state of freshwater systems all around the world~~. ~~Freshwater habitats tend to support relatively rich biodiversity at all trophic levels. This is evident, for example, from the large number of about 15,000 freshwater fish species known around the world (Levêque *et al.*, 2007; Nelson, 2006)~~. ~~As a result of human activities in and around freshwater systems, many fish species have become highly endangered through disturba~~

~~nce and diminishing of natural habitats (Darwall, Freyhof, 2016; Dudgeon *et al.*, 2006; Dynesius, Nilsson, 1994; Lind *et al.*, 2012; Nilsson *et al.*, 2005).~~

Field Code Changed

Effective management ~~of natural resources is based on information about its biotic and abiotic contents. The~~for the assessment of freshwater fish biodiversity and population status ~~as considerations for habitat management is~~is therefore a priority, however, can be considered a great challenge (Dudgeon *et al.*, 2006; Levêque *et al.*, 2007; Reid *et al.*, 2019; Sala *et al.*, 2000). Conservation measures intended to mitigate the impact of human derived pressures have largely been slow and inadequate, and as a result, populations of many freshwater species have been declining rapidly (Darwall, Freyhof, 2016; Dudgeon *et al.*, 2006; Lind *et al.*, 2012; Sala *et al.*, 2000). Further human derived pressure on natural fish diversity has also been~~was~~ documented due to introduction and invasion of exotic fish species (Casal, 2006; Havel *et al.*, 2015; Leprieur *et al.*, 2008; Thomaz *et al.*, 2015).

Due to its arid climatic conditions, Israel have been facing growing demands for freshwater. Consequently, its limited freshwater habitats have been under great human pressure. The Sea of Galilee (Lake Kinneret) is the biggest surface freshwater reservoir in the middle east (ca. 170 km2) and due to its crucial role in drinking water supply, it~~s water level and quality are~~ is highly regulated (Goren, Ortal, 1999; Markel *et al.*, 2014; Serruya, 2012). In terms of freshwater fish, about ~~19~~ 18 native and eight ~~exotic~~ non-native species were documented in the Sea of Galilee ; Ostrovsky *et al.*, 2014a). Native species are dominated by cyprinids and cichlids that are of mainly Asian (Mesopotamian) and African origins, respectively (Goren and Ortal, 1999). The native cyprinid Kinneret bleak *Mirogrex terraesanctae* (Steinitz, 1952)~~(Kinneret bleak)~~ is probably the most abundant species in terms of both numbers and biomass (Ostrovsky *et al.*, 2014b). Based on fishery landings, ~~From~~ the native cichlid~~s~~, Galilee tilapia or St. Peter's fish

Sarotherodon galilaeus (Linnaeus, 1758)~~Galilee tilapia or St. Peter's fish~~) is abundant, ~~the~~ most ~~abundant~~valuable and sought~~ for~~ after. While ~~*the Kinneret bleak*~~*M. terraesanctae* is endemic to Israel, *S. galilaeus* is widespread in central and western Africa (Lind *et al.*, 2012). ~~In terms of exotic fish, some~~Non-native species, which were unintentionally introduced, include the European eel~~.~~ *Anguilla anguilla* (Linnaeus, 1759)~~European eel~~) that hitchhiked with mullet fry caught in coastal estuaries and stocked into the ~~lake~~Sea of Galilee (Ben-Tuvia, 1981), hybrid tilapia ~~(~~*Oreochromis niloticus* (Linnaeus, 1758) x *Oreochromis aureus* (Steindachner, 1864) that escaped from aquaculture ponds (Goren and Galil, 2005) and *Cichla kelberi* Kullander and Ferreira, 2006 that was probably released in the ~~lake~~ Sea of Galilee by hobbyists (Golani *et al.*, 2019). ~~Other~~ The ~~exotic~~ non-native invasive mosquito fish *Gambusia affinis* (Baird and Girard, 1853)~~species were~~was intentionally introduced~~, for example, *Gambusia affinis*~~ to control mosquitoes (Goren and Ortal, 1999). Nevertheless, ~~the most~~ significant non-native species ~~introductions~~ which were introduced to enhance commercial fishing~~,~~ ~~including~~ included the common carp ~~(~~*Cyprinus carpio*~~)~~ Linnaeus, 1758, the silver carp ~~(~~*Hypophthalmichthys molitrix*~~)~~ (Valenciennes, 1844), the thin lip grey mullet ~~and two mullet species (*Liza*~~ *Chelon ramada* (Risso, 1827) and the flathead grey mullet *Mugil cephalus*~~)~~ Linnaeus, 1758 (Goren and Ortal, 1999; Goren and Galil, 2005).

The dynamics of pelagic fish populations in Sea of Galilee is monitored mainly by means of acoustic surveys that measure abundance and distribution of fish shoals and also estimate size of fish based on the echo target strength (Walline PD, Pisanty S, Lindem T (1992) Acoustic assessment of the number of pelagic fish in Lake Kinneret, Israel. Hydrobiologie 231:153–163). These acoustic surveys do not distinguish between species

but some inferences can be made based on estimated size of individuals and shoals. Based on these surveys, some estimates for population size of main pelagic species like the *M. terraesanctae* ~~Kinneret bleak~~ can be obtained (Ostrovsky *et al.*, 2014a). Other inference on the population size comes from the commercial landing of sized fish (Kantoussan *et al.*, 2014; Ostrovsky *et al.*, 2014b).

In recent years, DNA polymorphism, a more reliable and accurate method, is increasingly used for ~~taxonomy~~ taxonomic and ecological research (e.g. Hughes et al., 2014; Borovski et al., 2018; Ali et al., 2020). DNA sequences of specific genes are used successfully to identify biological species in a method called ~~species~~ DNA barcoding (Hebert *et al.*, 2003; Ward *et al.*, 2009; Ward *et al.*, 2005). As certain DNA sequences in genomes evolve at rates similar to evolution of new species, DNA barcoding can complement and enhance species identification. DNA barcoding based methods can also be utilized for efficient routine monitoring of freshwater fish biodiversity, as DNA can easily be extracted from various types of samples including from environmental water samples, known as E-barcoding (Pont *et al.*, 2018; Ushio *et al.*, 2018). Barcode sequences obtained from unidentified samples can be matched to available records from known samples for species identification. Thus, ~~The~~ the application of DNA barcoding for reliable molecular identification of species, requires a reference database that connects DNA sequences to specific species (Ekrem *et al.*, 2007; Weigand *et al.*, 2019). The Barcode of life Data systems (BOLD) is a comprehensive, parameterized DNA barcode reference library (Ratnasingham and Hebert, 2007), created to support the use of barcode data and molecular species identification for studying various aspects of biodiversity. ~~Thus, h~~Here, we applied DNA barcoding to samples taken mainly from the

Sea of Galilee to ~~address taxonomic questions and build~~ create a reference barcoding database, within the platform of BOLD, for molecular identification of fish in the context of the Sea of Galilee and the region, an important tool for management and conservation of fish populations.~~.~~

Methods

Sampling of fish species

To genetically barcode fish species ~~in the context of~~from the Sea of Galilee and surrounding areas, an extensive sampling scheme was carried out to obtain samples from each of the species that could be found in the lake. The Sea of Galilee, the main sampling site, and surrounding sites such as Asi stream (one of Beit She'an streams, south to the Sea of Galilee and part of the eastern watershed of Israel), Ein Afek pools (close by Acre, i.e. Akko, part of the western watershed of Israel) and Te'o spring (close by Hula Lake, northern to Sea of Galilee and part of the eastern watershed of Israel) (see figure 1A by (Borovski *et al.*, 2018)), were sample~~s~~d throughout 2013, 2014 and 2015 to collect samples of species that predominantly occur in this region of Israel by means of seine netting. Number of individual samples collected for each site and species are recorded in Table 1. ~~As part of a previous research [31] about 12 sampling attempts were done in the Sea of Galilee during and a few additional attempts were carried out in Beit She'an streams (south to the lake, and part of the eastern watershed of Israel), Ein Afek pools [close by Acre (Akko), part of the western watershed of Israel] and Te'o spring (close by the Hula Lake, northern to the Kinneret and part of the eastern watershed of Israel)~~

~~[described in more detail in [31]]~~. Fish were anesthetized with 2-phenoxy-ethanol and after taxonomically identified in the field to the species level, ~~and~~ a fin clip was taken from every fish. In most cases, after identification and sampling, fish were revived from anesthesia and returned to their environment. Some fish were also sacrificed, photographed and deposited in the National Natural History Collections of the Hebrew University of Jerusalem, Israel as detailed in Table 1. The fins were stored in labeled 2 mL Eppendorf tubes in 100% ethanol at -20°C, entire fish that were deposited in the collections were preserved in formalin.

Table 1. Fish collected and barcoded by species and location. Given also are numbers and museum numbers deposited at the National Natural History Collections in the Hebrew University (NNHC-HUJ).

Species	Samling location	N samples collected	N Samples barcoded	N Specimens deposited	Museum numbers at NNHC-HUJ
Clarias gariepinus	Sea of Galilee	8	5	0	
Mirogrex terraesanctae	Sea of Galilee	10	8	0	
Luciobarbus longiceps	Sea of Galilee	10	7	0	
Acanthobrama telavivensis	Ein afek	13	7	3	HUJ21008
Capoeta damascina	Sea of Galilee	5	5	0	
Cyprinus carpio carpio	Sea of Galilee	14	5	0	
Garra rufa	Sea of Galilee	8	8	3	HUJ21009
	Beit She'an streams	4	3	0	
	Ein T'eo	6	3	0	
Garra nana	Sea of Galilee	2	2	0	
Carasobarbus canis	Sea of Galilee	9	6	0	
	Ein T'eo	5	5	0	
Hypophthalmichthys molitrix	Sea of Galilee	23	23	0	
Hypophthalmichthys molitrix Suspected as hybrids with *Hypophthalmichthys nobilis*	Sea of Galilee	6	6	0	
Pseudophoxinus kervillei	Sea of Galilee	1	1	0	
Oxynoemacheilus leontinae	Sea of Galilee	5	3	0	
Gambusia affinis	Sea of Galilee	7	3	0	
Aphanius mento	Sea of Galilee	1	1	0	
Salaria fluviatilis	Sea of Galilee	10	8	0	
Mugil cephalus	Sea of Galilee	10	7	0	
Chelon ramada	Sea of Galilee	13	9	0	
Astatotilapia flaviijosephi	Beit She'an streams	10	8	3	HUJ21007

Coptodon zillii	Sea of Galilee	29	8	2	HUJ21010
Tristramella simonis	Sea of Galilee	14	6	0	
Sarotherodon galilaeus	Sea of Galilee	120	17	9	HUJ21012
	Ein afek	19	4	0	
Oreochromis aureus	Sea of Galilee	11	7	2	HUJ21011
Oreochromis aureus Suspected as hybrids with *Oreochromis niloticus*	Sea of Galilee	1	1	0	

DNA extraction, polymerase chain reaction (PCR), purification and sequencing

DNA from stored fin samples was extracted using a modified protein salting-out method (Lopera-Barrero et al., 2008)~~(Martínez *et al.*, 1998)~~. ~~In brief, approximately 5 mg of dry fin sample was digested at 50 °C in 550 µL cell lysis solution (50 mM Tris–HCl, pH 8.0, 50 mM EDTA, 100 mM NaCl) with 1% SDS and 1 µL of Proteinase K solution (20 mg/mL). To precipitate the proteins, 300 µL of 5 M NaCl were added and the sample was vortexed and centrifuged. DNA pellet was obtained by mixing with 900 µL of freezer-cold 100% isopropanol, 2 h incubation at −20 °C and centrifugation. The pellet was washed with 700 µL of 70% ethanol, dried for 15 min and dissolved overnight in 100 µL of double-distilled water at room temp.~~ DNA concentration and quality (Optical Density OD_{260}/OD_{280} ratio) were measured using NanoDrop ND-1000 (NanoDrop Technologies) and visually examined by 1.5% TBE Agarose gel electrophoresis. DNA samples were stored at −20°C until used for further analysis.

~~PCR procedures and sequencing of PCR products~~

A partial fragment of approximately 700bp of the *Cytochrome C oxidase subunit I* (COI), a mitochondrially encoded gene, was amplified by PCR using primers adopted from

(Ward *et al.*, 2005) (~~cox1a_fw~~FishF1: 5'-TCAACCACCCACAAAGACATTGGCAC-3' and ~~cox1a_rv~~FishR1: 5'-TAGACTTCTGGGTGGCCAAAGAATCA-3'). As DNA template, 50 ng of purified DNA were used in a total reaction volume of 20 µL. PCR mix contained: 2 µL 10× PCR buffer, $MgCl_2$ (25mM), dNTPs (6.6mM of each), Taq polymerase (1.5 units), primer mix (forward + reverse, 2.5 µM of each) and 10 µL water to complete the volume. PCR ~~profile~~ thermal protocol included an initial step of 94 °C for 2 min; followed by a touchdown profile of 94 °C for 30 s, annealing from 60 to 53 °C for 1 min with a decrease of 0.5 °C per each of 14 cycles, and extension at 72 °C for 2 min; further 23 cycles at the lower annealing temperature and final elongation step at 72 °C for 10 min. PCR products were verified by electrophoresis using 1.5% TBE Agarose gel containing ethidium bromide. PCR products were visualized using Gel Doc XR+ (BIO-RAD) and using ImageLab software (BIO-RAD). Successful PCR products were ~~cleaned~~ purified using an Exonuclease I-Shrimp Alkaline Phosphatase (ExoSAP-IT) method (USB, Cleveland, OH) and Sanger sequenced using BigDye V3.1 (Applied Biosystems) terminator chemistry in the forward and reverse directions on an ABI PRISM 3730xl DNA Analyzer (Applied Biosystems) following the manufacturers protocol.

Data analysis

COI sequence files were assembled and aligned using clustalW algorithm with BioEdit software (Version 7.2.5) (Hall, 1999). All the polymorphisms detected by the software were manually inspected on the chromatograms and dubious cases were manually removed. Successful sequences for each species together with ~~C~~their collection data ~~and~~

~~barcode sequences~~ were deposited into Barcode Of Life Data system (BOLD, https://www.boldsystems.org) (Ratnasingham, Hebert, 2007) under project name LKCOX. See number of deposited sequences for the different species in Table 1. Next, each haplotype for each species was queried against species level barcode records using BOLD identification module to derive molecular species identification. ~~However, i~~In some cases, the molecular identification did not completely match the original morphological species identification. For species where morphological and barcoding identification matched, only the barcodes of sampled fish were considered. For species with disagreements, also barcodes of the same and related species were downloaded from BOLD and considered in further sequence-based analyses. Aligned sequences of all specimens were manually inspected, and polymorphic sequences from each species were ~~regarded~~defined as different Haplotypes. An estimated unrooted gene tree based on COI haplotypes consisting on a total of 530 positions, was constructed utilizing the Molecular Evolutionary Genetics Analysis (MEGA6) freeware (Tamura *et al.*, 2013), using the Kimura-2-parameter model, and the neighbor-joining tree construction method with 1000 bootstrap replicates (Felsenstein J. (1985). Confidence limits on phylogenies: An approach using the bootstrap. Evolution 39:783-791.). The tree with the strongest bootstrap support was chosen for representation. Based on ~~the~~ all COI haplotypes and using MEGA6 software, ~~the~~ K2P genetic distance between any pair of haplotypes was calculated. The average pairwise differences ~~within and between different taxa~~ were manually calculated ~~from~~ by summing up and averaging the pairwise haplotype distances within and between different taxa. Fish that were suspected to be hybrids according to their morphology were ~~analyzed separately~~excluded from the average taxa calculations.

Ethical approval

Fish sampling from protected nature reserves was done under special permits 2012/38733 and 2014/40233 for sampling protected wildlife as reviewed and approved by the Israel Nature and National Parks Protection Authority. Some samples were obtained from catches of a commercial fishing vessel.

Results

Molecular barcoding

To establish a molecular database for identifying the fish of the Sea of Galilee, COI barcode sequences were determined for randomly collected fish samples. Samples and barcode sequences were obtained for a total of 22 fish species, including21 16 out of the 25 18 native fish species reported in the lake Sea of Galilee (Goren, Ortal, 1999; Ostrovsky *et al.*, 2014a; Ostrovsky *et al.*, 2014b), five non-native species which inhabit the Sea of galilee and *Acanthobrama telavivensis* Goren, Fishelson & Trewavas, 1973 which was sampled outside the Sea of Galilee for comparison to the closely related *Acanthobrama lissneri* Tortonese, 1952, (Goren, 1973) one of the native fish species that wascould not ben't found in any of the Sea of Galilee sampling schemes. One of the fourAnother native fish species absent from this study, *Tristramella sacra* (Gunther, 1865), is considered to be extinct (Goren, 2014). Notably missing from the non-native species and the other three (were *Acanthobrama lissneri* Tortonese, 1952, *Anguilla anguilla* (Linnaeus, 1758) and *Xiphophorus hellerii* (Heckel, 1848) that were could not

be found in any of the Sea of Galilee sampling schemes. Although *A. lissnerii* was not included in this study, the closely related species *Acanthobrama telavivensis* Goren, Fishelson & Trewavas, 1973 [36] was sampled outside the lake for comparison. Altogether, COI sequences were analyzed from 182 176 individual fish, belonging to 22 freshwater species (Table 2). Additionally, 20 sequences were analyzed from two fish species, *H. molitrix* (19 sequences) and *O. aureus* (one sequence), that were suspected as hybrids with aquaculture fish (hereafter referred to as ‘suspected hybrids’) (Table 2). Collection data and barcode sequences were deposited in Barcode Of Life Data system (BOLD) [37] under project name LKCOX.

For 20 out of 22 species more than one specimen was collected and sequenced, and out of those, 12 species had more than one barcode sequence variant (COI haplotype). Therefore, K2P distances within species were calculated for each species and ranged between 0 – 0.0086 with a mean of 0.0017 (Table 2). For ten different species, A a total of 12 barcode sequences were novel barcodes not deposited before in the BOLD public database derived from ten different species, were novel barcodes not deposited before in the BOLD public database (Table 12). For some species with barcodes existing in BOLD, we added new ones (different haplotypes). These added barcodes haplotypes were always those with the lower frequency in our data (Table 12).

Table 12. Summary of fish species, number of barcoded samples, used in COI barcoding, their haplotypes naming, haplotype frequencies and within species genetic distances. Here the species of fish is based on morphological taxonomy.

Order	Family	Species	N barcoded samples	Haplotype	Haplotype frequency	K2P distance within species
Siluriformes	Clariidae	*Clarias gariepinus*	5	CgA	1	0
Cypriniformes	Cyprinidae	*Mirogrex terraesanctae*	8	AtA	0.625	0.0019
				AtB*	0.125	
				AtC	0.125	

				AtD	0.125	
		Luciobarbus longiceps	7	BlA	0.571	0.0019
				BlB	0.429	
		Acanthobrama telavivensis+	7	AtlA	0.571	0.0019
				AtlB*	0.429	
		Capoeta damascina	5	CdA	0.4	0.0038
				CdB	0.4	
				CdC*	0.2	
		Cyprinus carpio carpio	5	CcA	1	0
		Garra rufa	14	GrA	0.571	0.0086
				GrB*	0.214	
				GrC	0.143	
				GrD*	0.072	
		Garra nana	2	GnA	1	0
		Carasobarbus canis	11	BcA	0.909	0.0019
				BcB*	0.091	
		Hypophthalmichthys molitrix	~~36~~23	HmA	0.~~75~~783	0.0057
				HmB	0.~~25~~217	
		*Hypophthalmichthys molitrix***	~~19~~6	HmA	0.~~158~~333	0.0362
				HmB	0.~~158~~5	
				HmC	0.~~684~~167	
		Pseudophoxinus kervillei	1	PkA*	1	0
	Nemacheilidae	*Oxynoemacheilus leontinae*	3	NlA*	0.667	0.0019
				NlB*	0.333	
Cyprinodontiformes	Poeciliidae	*Gambusia affinis*	3	GaA	1	0
	Cyprinodontidae	*Aphanius mento*	1	AmA*	1	0
Perciformes	Blenniidae	*Salaria fluviatilis*	8	SfA	0.875	0.0038
				SfB*	0.125	
Mugiliformes	Mugilidae	*Mugil cephalus*	7	McA	1	0
		Chelon ramada	9	LrA	0.889	0.0019
				LrB	0.111	
Cichliformes	Cichlidae	*Astatotilapia flaviijosephi*	8	AfA	0.75	0.0025
				AfB*	0.125	
				AfC	0.125	
		~~*Tilapia*~~ *Coptodon zillii*	8	TzA	1	0
		Tristramella simonis	6	TsA	0.833	0.0019
				TsB	0.167	
		Sarotherodon galilaeus	21	SgA	1	0
		Oreochromis aureus	7	OaA	1	0
		*Oreochromis aureus****	1	OaB	1	0

+Not known to be found in the ~~lake~~Sea of Galilee, closely related to *Acanthobrama lissneri*.
*Newly deposited haplotypes
** Suspected hybrid of *Hypophthalmichthys molitrix* and *Hypophthalmichthys nobilis*
*** Suspected hybrid of *Oreochromis niloticus* and *Oreochromis aureus*

~~Next, each haplotype for each species was queried against species level barcode records using BOLD identification module to derive molecular species identification.~~ For ~~most~~ 19 out of ~~our~~ 22 species, the molecular identification agreed with the original morphological identification, including for species with more than one COI haplotype.

~~However, in some cases the molecular identification did not completely match the original morphological species identification.~~

Disagreements with molecular barcoding

~~For each species with~~Three species were found to have ~~disagreement between morphological and molecular barcoding identification,~~. ~~further~~ Further ~~investigation was carried out using all available COI barcode records from BOLD of query and related species.~~ Three species were found to have disagreements between morphological and molecular barcoding identification. Further investigation was carried using all available COI barcode records from BOLD of query and related species.~~–~~ *Acanthobrama*~~*A.*~~ *telavivensis* fish ~~we~~ sampled from Ein Afek spring pools had two haplotypes with similar frequencies, one of which was new. The older haplotype, common to our data and BOLD, was identified by BOLD identification module as either *A. lissneri* or *A. telavivensis* (100% fit for both). Four *A. lissneri* and one *A. telavivensis* records were available from BOLD, all of them sharing an identical sequence. ~~Thus, molecular discrimination between these two species using COI barcoding might not be possible. *A. lissneri* was reported as one of the native Sea of Galilee species, whereas *A. telavivensis* was not. Although, *A. lissneri* from the lake was not included in our sampling, the results of our molecular analysis likely apply also to the lake *A. lissneri* population.~~

~~We collected t~~Three *Gambusia affinis* samples collected, all with the same haplotype (GaA), ~~which was~~was identified by BOLD ~~identification module~~ as *Gambusia holbrooki* Girard, 1859 (100% fit), followed by *G. affinis* (99.81% fit). Comparison to all available *G. holbrooki* and *G. affinis* records from BOLD indicated that GaA haplotype

clusters together with *G. holbrooki* records. ~~and thus, *G. affinis* from the Sea of Galilee sampled in this study and possibly more, may be *G. holbrooki*, a species that was not reported in the lake.~~

Garra rufa had 14 samples in our collection, showing four haplotypes, of which two were new. BOLD ~~identification module~~ identified our samples as *Garra jordanica* Hamidan, Geiger & Freyhof, 2014 (98.7-100% match for different haplotypes). This disagreement will be ~~more thoroughly addressed hereafter~~discussed further later under its own sub-section.

~~Interestingly, in samples of fish suspected as hybrids, molecular~~ Molecular identification using BOLD for samples of fish suspected as hybrids provided genetic evidence to support hybridization. A sample suspected as *Oreochromis niloticus* x *Oreochromis aureus* hybrid, which is neither native fish to nor stocked in~~to~~ the ~~lake~~Sea of Galilee, had a COI sequence with perfect match (100%) to *O. niloticus*, indicating that this fish is at least partly hybridized with the non-native *O. niloticus*. ~~This is consistent with the breeding scheme used for production this hybrid for aquaculture, where the female parents used are *O. niloticus*.~~ Additionally, in samples that according to their morphology were suspected to be *Hypophthalmichthys*~~*H.*~~ *molitrix* hybridized with *Hypophthalmichthys*~~*H.*~~ *nobilis*~~*Nobilis*~~ (Richardson, 1845), three different COI haplotypes were found. Two of them matching *H. molitrix*, while the third matching *H. nobilis* (100%), supporting possible hybridizations in both directions~~, –~~. ~~again often used for aquaculture fish. –~~K2P distance within these hybrid fish was 0.0362, ~~almost~~ more than ~~10~~4-fold higher than the maximum distance found within the other species, ~~another~~

further evidence to support that these fish were hybrids and such hybrids are in the ~~lake~~Sea of Galilee.

Genetic distances from barcoding data

To evaluate the DNA barcoding efficiency in distinguishing among species, K2P genetic distances between each pair of species was calculated. The genetic distance between species ranged between 0.0095 – 0.2886, with a mean distance of 0.2295, 100-fold higher compared to the mean within species K2P distance (0.0017). Additionally, the range of between-species distances did not overlap with that of within-species, supporting the utility of COI haplotypes in species differentiation. The species differentiation is evident also from the phylogenetic tree based on the COI haplotypes (Fig 1). In the tree, species were grouped similarly to the expected tree based on the NCBI taxonomy browser (https://www.ncbi.nlm.nih.gov/Taxonomy/taxonomyhome.html/). The phylogeny separates well all the different species, while grouping together multiple haplotypes of the same species. Furthermore, on the higher levels of taxonomy, the phylogeny separates well the multiple cyprinid species from cichlids or mugilids, the three most represented families in the ~~lake~~Sea of Galilee.

Fig 1. A neighbor-joining un-rooted ~~Dendrogram~~ tree of species based on COI haplotypes. Next to the tips of edges, the haplotype acronyms are given (see Table ~~1~~ 2 for acronyms). Numbers next to edges denote percent bootstrap support. For some species, more than one haplotype was identified (denoted A, B etc.). For major families, which are represented by multiple species, a mean K2P between species distance matrix is given to the right. (*) – HmC haplotype derived from specimens suspected as *Hypophthalmichthys molitrix* x *Hypophthalmichthys nobilis* hybrids, matches *H. nobilis* sequences from BOLD. (**) - OaB haplotype derived from a specimen suspected as

Oreochromis niloticus x *Oreochromis aureus* hybrid, matches *O. niloticus* sequences from BOLD.

~~Interestingly, some unexpected~~ Unexpected results were, however, found ~~in~~ within the *Cichlidae* clade of the neighbor-joining phylogenetic tree. *O. aureus* (Haplotype OaA) clustered together with *S. galilaeus* (haplotype SgA), a species belonging to a different genus, rather than with *O. niloticus* (haplotype OaB) with which its genus is shared. Support comes from K2P distances between *O. aureus* ~~(OaA)~~ and *S. galilaeus* ~~(SgA) is~~ (0.0095)~~,~~ which is smaller in comparison to distances between other cichlids of ~~the l~~L~~ake~~Sea of Galilee belonging to different genera~~, the distances of which range between~~ (0.06-0.16), and ~~even~~ also smaller compared to cyprinids belonging to different genera~~, the distances of which range between~~ (0.08-0.23) (Fig 1). ~~Therefore, molecular data suggests the taxonomy of these cichlid groups should be studied more extensively.~~

Within the cyprinid clade, *Garra*~~.~~ *rufa* and *G*~~.~~ *arra nana* (Heckel, 1843), which are species of the same genus, have K2P distance of 0.08, which indeed is a smaller value than ~~we find~~ between genera. ~~However, i~~Identical K2P distance was, however, found between *A*~~.~~ *canthobrama telavivensis* and *Mirogrex terraesanctae*.~~, formerly known as *Acanthobrama* *A.* *terraesanctae* and later moved to a separate genus [36]. This relatively low distance suggests the *Acanthobrama* – *Mirogrex* taxonomic separation could be reconsidered.~~

Species in the *Garra* ~~*spp.* complex~~genus

~~As mentioned before, c~~Comparison of *G~~.~~arra rufa* haplotypes obtained in this research with BOLD database accessions gave inconsistencies in species definition. In addition, K2P distance within haplotypes of fish identified as *G. rufa* in this study was 0.0086, the highest value found within species groups and more than four times higher than the mean within-species value (0.0014) of all other species groups excluding *G. rufa*. ~~Samples identified morphologically as~~ *G~~.~~arra rufa* fish were collected in this study from three places: Sea of Galilee, Asi stream (one of Beit She'an streams) and Ein Te'o (North of the Sea of Galilee) (Fig 2A). These fish had four COI haplotypes (GrA – GrD) that were unevenly distributed between collection sites. ~~the~~ The Sea of Galilee samples had~~,~~ five fish from Haplotype ~~had~~ 'GrA', two fish from Haplotype ~~had~~ 'GrC' and one fish from Haplotype ~~had~~ 'GrD' ~~haplotypes~~. All three fish from Ein Te'o ~~(north of the lake) fish had~~shared Haplotype 'GrA' ~~haplotype. The~~and all three fish from Asi stream ~~(Beit She'an valley) fish~~ shared ~~a unique~~Haplotype 'GrB' ~~haplotype~~ (Fig 2A).

Fig 2. Sampling sites and clustering of *Garra spp.* ~~and clustering of~~ samples based on COI haplotypes. Acronyms of populations are: GrA – *Garra~~.~~ rufa* from Haplotype A; GrB – *Garra rufa* from Haplotype B; GrC – *Garra rufa* from Haplotype C; GrD – *Garra rufa* from Haplotype D;~~(Northern Jordan valley, Asi stream in Beit She'an valley and Asian countries),~~ Gg – *G~~.~~arra ghorensis* ~~(~~from the southern basin of the Dead sea~~—southern basin);~~ Gj – *G~~.~~arra Jordanica* ~~(~~from the northern basin of the Dead sea;~~—northern basin) and~~ GnA – *Garra nana from Haplotype A; GnB – Garra nana from Haplotype B.* ~~*G. nana.*~~ **(A)** Map showing the sampling ~~locations~~ sites in ~~the~~ a regional context. Different *Garra* ~~*spp.*~~ ~~haplotypes~~ H~~h~~aplotypes were denoted by differently colored ~~points~~ circles in each sampling site on the map of Israel, ~~and~~ Jordan and Syria regions. Map of Israel and the region is reprinted from DMY + NordNordWest (https://commons.wikimedia.org/wiki/File:Israel_by_israeli_law_adm_location_map.svg) , under a CC BY-SA 4.0 license, original copyright 2017. Map of the middle east is reprinted from Виктор B (https://commons.wikimedia.org/wiki/File:Outline_map_of_Middle_East.svg) under a CC BY-SA 2.0 license, original copyright 2010. **(B)** A neighbor-joining un-rooted

dendrogram ~~was~~ constructed for ~~haplotypes~~ ~~H~~haplotypes obtained from samples collected in this study alongside ~~haplotypes~~ ~~H~~haplotypes mined from BOLD (see ProcessID's in table S1). Next to the ~~tips of~~ edges, ~~the~~ haplotype acronyms are given as indicated above. Numbers next to edges denote percent bootstrap support. Colors of ~~haplotype~~ ~~H~~haplotype text match the color of ~~points~~ circles on the map (A).

~~To put in context the *Garra spp.* samples from Israel, s~~Sequences of 16 samples from this study together with 33 BOLD accessions, which had ~~a~~ geographic location information assigned to them (see Table ~~S1~~S2), were aligned to construct a neighbor-joining phylogenetic tree (Fig 2B). Haplotypes of *G. nana* were separated from all other haplotypes defined as *G. rufa*, *G. jordanica* and *G*~~.~~ *arra* ~~*Ghorensis*~~*ghorensis* Krupp, 1982. The two haplotypes of *G. nana* (GnA and GnB) were relatively distinct from one another (K2P = 0.0381; Table 3)~~ and were found in fish from two isolated locations (Fig 2A)~~. GnA, ~~the *G. nana* haplotype~~was found in fish from the Sea of Galilee ~~was found also~~and ~~in samples from a~~the more northern location in Syria, while GnB, was found in fish from an isolated location more southern in Syria (Fig 2A).

Israel~~i~~ *G. rufa* samples from the Sea of Galilee and Ein Te'o ~~(north of the lake)~~, ~~which were morphologically identified as *G. rufa*~~, had ~~the haplotypes~~ ~~H~~haplotypes GrA, GrC and GrD. These Israel~~i~~ *G. rufa* ~~haplotypes~~ ~~H~~haplotypes clustered with BOLD ~~haplotypes~~ Haplotypes of *G. jordanica* (haplotype Gj), fish sampled from streams in the northern basin of the Dead Sea in Jordan (Fig 2B). Close by, yet separated, in the phylogeny, was *G. rufa* fish, ~~haplotype~~ Haplotype GrB, ~~of fish~~ from Asi stream ~~(Beit She'an valley)~~, ~~morphologically defined as *G. rufa*,~~ suggesting again greater similarity to *G. jordanica*, but some genetic separation from the ~~lake~~ Sea of Galilee population. Further separated from this cluster of Israeli *G. rufa* and non-Israeli *G. jordanica* BOLD samples was the BOLD *G. ghorensis* haplotype (Gg) of fish from streams in the southern

basin of the Dead Sea. Further away, clustered together were BOLD haplotypes (GrE – GrN) of fish defined as *G. rufa* from other Asian countries (GrAsia - Turkey, Iran and Iraq). ~~Thus, these results indicate that species differentiation is consistent with geographic separation of *Garra spp.* and that the species definition of Israeli *G. rufa* fish should be reconsidered.~~

Table ~~2~~3. Genetic distances between geographically separate *Garra* ~~*spp.*~~ populations. In each table cell, top row is the mean K2P value and bottom row is the range of pairwise distances.

	Gr North	Gr Asi	Gj	Gg	Gr Asia	GnA	GnB
Gr North	0.0025<br>0 - 0.0037						
Gr Asi	0.0143<br>0.013-0.0168	0					

~~*~~Acronyms for populations are: GrNorth – *G.~~-~~arra rufa* ~~(~~from Sea of Galilee and Ein Te'o of Northern Jordan valley~~, -~~;GrAsi – *Garra rufa* from Asi atream ~~in~~ of Beit She'an valley; Gj – *Garra jordanica* from the northern basin of the Dead Sea; ~~and Asian countries),~~ Gg – *G.~~-~~arra ghorensis* ~~(~~from the ~~Dead sea –~~ southern basin~~)~~ of the Dead Sea; GrAsia – *Garra rufa* from Asian countries including Turkey, Iran and Iraq; ~~Gj – G. Jordanica (Dead sea – north basin) and~~ GnA – *G.~~-~~arra nana* from Haplotype A; GnB – *Garra nana* from Haplotype B.

Gj	0.0031<br>0.0018-0.0055	0.0149	0				
Gg	0.0414<br>0.0401-0.0441	0.0381	0.0421	0			
Gr Asia	0.0462<br>0.0422-0.0523	0.0469<br>0.0442-0.0502	0.0469<br>0.0442-0.0502	0.0587<br>0.0563-0.0604	0.0065<br>0.0018-0.013		
GnA	0.0792<br>0.0792-0.0792	0.0857	0.0814	0.0834	0.0635<br>0.0582-0.0685	0	
GnB	0.0899<br>0.0899-0.0899	0.0965	0.0921	0.0854	0.0692<br>0.0664-0.727	0.0381	0

Given the genetic divergence found for geographically isolated fish, pairwise K2P distances were calculated for *Garra* ~~*spp.*~~ and compared to the distances found within and between species based on all species (Table ~~1~~2, Fig 1). For each combination of species and geographic location the mean, minimum and maximum values of the pairwise K2P distances were calculated (Table ~~2~~3). It is evident that the genetic separation between populations of *G. rufa* increases with geographic distance. The Israeli *G. rufa* fish, ~~morphologically defined as *G. rufa*, from the northern Jordan valley (Sea of Galilee, Ein T'eo in the Hula valley and Asi stream in the Beit she'an valley)~~ are ~~quite~~ somewhat genetically divergent from *G. rufa* fish from farther eastern and northern Asian countries (~~Iran, Iraq and Turkey~~Mean K2P between Gr North and Gr Asia = 0.0462, and between Gr Asi and Gr Asia = 0.0469; Table 3). The mean K2P value between these ~~two~~ *G. rufa* clades is considerably higher than what was observed as within-species differences (Table ~~1~~2), and more similar to between-species ones (Fig 1). On the other hand, the haplotype of *G. jordanica* from BOLD clustered in the phylogenetic tree together with haplotypes of Israeli *G. rufa* fish ~~from the Northern Jordan valley (~~(Gr North; Fig 2). The

mean K2P distance between these two groups was very low (0.0031, Table 23) and well within the range of within-species distances (Table 12).

Within Israeli *G. rufa*, fish from the Asi stream of Beit she'an valley (Gr Asi)(Asi stream) had a slightly different haplotype (GrB) then their neighboring fish from Sea of Galilee and Ein T'eo in the Hula valley (mean K2P between Gr Asi and Gr North of = 0.0143) and thus, also slightly genetically different from BOLD samples defined as *G. jordanica* (mean K2P between Gr Asi and Gj of = 0.0149) (Table 23). These mean K2P distances are slightly higher than within-species distances (Table 12) and considerably lower than between-species differences (Fig 1).

Samples from BOLD of *G. ghorensis* fish from the southern part of the Jordan valley were similarly genetically distant from samples of *G. rufa* from Asia (mean K2P between Gg and Gr Asia = 0.0587) and from Israeli *G. rufa* (K2P of 0.0587 and mean K2P ofbetween Gg and Gr North = 0.04210414 and between Gg and Gr Asi = 0.0381, respectively,) (Table 23). Finally, samples of *G. nana* were genetically distant from *G. rufa*, *G. jordanica* and *G. ghorensis* (Table 23) at levels similar to between-species distances (Fig 1). From all *Garra* populations, *G. nana* samples were the least distant from Asian *G. rufa* samples (Table 23).

Discussion

The development of molecular methods and their availability have been driving an effort to augment morphological taxonomy of species with molecular taxonomy based on DNA sequencing (Coleman and Radulovici, 2020; Hajibabaei *et al.*, 2007; Hebert *et al.*, 2003). Since within fish there are species-rich and diverse taxonomic groups, DNA barcoding

has great potential to improve the taxonomy within these groups~~of fish is not only an important part of the barcode of life (BOL) initiative~~ (Hubert et al., 2008; Ward *et al.*, 2009)~~, but also has great potential to improve the taxonomy within these groups~~. In a previous study conducted by~~, the cichlids of Israel were barcoded~~ (Shirak *et al.*, 2009), the cichlids of Israel were barcoded with success. In this study, focus was given to the fish found in the Sea of Galilee and surrounding water bodies in the region, including species other than cichlids. Altogether, COI sequences from ~~182~~ 176 individual fish, belonging to 22 morphologically-defined freshwater species, were analyzed. Except for a few discrepancies which will be discussed later, for 19 out of the 22 species, morphological and DNA barcoding agreed on the species definition. In theory, the 22 species, as they were defined by morphological taxonomy, should have returned 22 DNA barcodes, but in practice, 40 different COI haplotypes were recovered. For 12 species, mostly belonging to the Cyprinidae and Cichlidae, two or more COI haplotypes were found, indicating that genetic variation had evolved not exactly at the same rate as species did.

The mean K2P genetic distance within species was small, corroborating within-species ranges of other studies (Mojekwu et al., 2021; Fawzia et al., 2020); Hebert *et al.*, 2003; Hubert *et al.*, 2008). Furthermore, the range of genetic distances between species did not overlap the range within species. Therefore, although more than one COI haplotype was found for some of the species, there were no indications that merging of distinct morphologically-defined species should be considered. Thus, for the Sea of Galilee, COI sequencing provides a reliable method to identify species.

Defining the expected K2P genetic distance (mean and range) for different haplotypes within and between species (or higher taxonomic levels), can provide a useful tool for ecological and taxonomic research (Hebert et al., 2003; Ward et al., 2009). Here we used phylogeny, genetic distances and geographic information to study a few discrepancies identified between morphological taxonomy and DNA barcoding-based taxonomy. Two species of the same genus, *A. lissneri*, reported native to the lake, and *A. telavivensis*, reported only outside the lake, shared one of the barcodes found for *A. telavivensis*. The other barcode of *A. telavivensis* was also very similar, suggesting high genetic similarity and possible reconsideration of their taxonomy. Also, regarding their taxonomy, a relatively small genetic distance, typical of distances between species, was found between *A. lissneri/telavivensis* and *M. terraesanctae*, two species of different genera, suggesting these genera are closer than expected. Thus, smaller than expected molecular divergence was found both between species of the same genera and between species of different genera, all belonging to this family. It should be noted that the genus of *Mirogrex terraesanctae* was originally defined as *Acanthobrama terraesanctae* (Steinitz, 1952; Goren, 1973) and the molecular data supports the original classification. Another species, the mosquito fish *G. affinis*, invasive to the Sea of Galilee, had identical barcode to BOLD barcodes of *G. holbrooki* from various other locations. As these fish were introduced into several freshwater bodies to combat mosquito infestations, it might be worthwhile to reconsider if indeed they deserve to be separate species.

Morphological taxonomy of African cichlids, a very species-rich group of fish, was occasionally revisited (Trewavas, 1983; Nyingi et al., 2021; Dunz et al., 2013). Cichlids in the Sea of Galilee are of ancient African origin and they constitute a key

Commented [LD1]: Steinitz, H. 1952 (1 Mar.) [ref. 12806]
Acanthobrama terrae-sanctae, sp.n., from Lake Tiberias, Israel. Annals and Magazine of Natural History (Series 12) v. 5 (no. 51) (art. 33): 293-298. [Date of publication from Evenhuis 2003:52 [ref. 27365].]
Goren M (1973) The cyprinid fishes of Acanthobrama Heckel and related genera

Commented [LD2]: Trewavas E (1983) Tilapiine fishes of the genera *Sarotherodon, reochromis* and *Danakilia*. British Museum (Natural History), London
Nyingi, Wanja D., et al. "Identifying and conserving tilapiine cichlid species in the twenty-first century." *The Behavior, Ecology and Evolution of Cichlid Fishes*. Springer, Dordrecht, 2021. 285-312.
Dunz AR, Schliewen UK (2013) Molecular phylogeny and revised classification of the haplotilapiine cichlid fishes formerly referred to as "*Tilapia*". Mol Phylogenet Evol 68:64–80

group in the lake, with both ecological and commercial significance. Here, phylogeny proximity and small genetic distance, on the order of between species of the same genera, were found between *O. aureus* and *S. galilaeus*, two species from different genera. This level of molecular relatedness was considerably smaller compared to the distance between *O. aureus* and *O. niloticus*, which share the same genus. Thus, such and other molecular methods can help further revisiting and updating cichlid phylogeny (Nyingi et al., 2021). Furthermore, in this study a fish identified as *O. aureus* was molecularly identified as *O. niloticus*, suggesting hybrids might exist in the lake, since pure *O. niloticus* are considered too cold-temperature sensitive to establish in the lake. Also, indication for the presence of hybrid fish between *H. molitrix* and *H. nobilis* were found. Both these cichlid and carp hybrids are commonly used in Israeli aquaculture and such, fish might have somehow reached into the lake by introduction or as escapees from nearby ponds.

However, phylogeny, genetic distances and geographic information were mainly employed for ~~to~~ studying the regional *Garra* ~~*spp.*~~ taxonomy, for which COI sequences of samples collected in this study were analyzed together with sequences from the public BOLD database. Presence of *G. rufa* and *G. nana* has been reported in the upper Jordan valley system (including in the Sea of Galilee, Hula valley and Beit She'an valley streams). *G. jordanica* and *G. ghorensis* were reported as endemic to freshwater springs and streams northern and southern to the Dead Sea, respectively (Goren, Ortal, 1999; Hamidan, Mir, 2003; Ostrovsky *et al.*, 2014a). The phylogenetic tree based on COI sequences suggested some level of geographic separation between groups of haplotypes.

Commented [LD3]: Nyingi, Wanja D., et al. "Identifying and conserving tilapiine cichlid species in the twenty-first century." *The Behavior, Ecology and Evolution of Cichlid Fishes*. Springer, Dordrecht, 2021. 285-312.

Small genetic divergence was found between *G. rufa* from the Asi stream (Beit She'an valley) south to the Sea of Galilee and the more northern Israeli fish (those from the ~~lake~~ Sea of Galilee itself and from the Hula valley). This minor genetic divergence within the northern Israeli fish, is likely due to the recent (~24,000 years ago) isolation of the Beit She'an valley system from the Jordan River system (Stein, 2014. Lake kinneret book).

Furthermore, *G. rufa* from Turkey, Iran and Iraq formed a cluster separated from Mediterranean *Garra* ~~*spp.*~~ samples. The range of K2P distances (0.0422-0.0523) suggest that Asian *G. rufa* and Mediterranean *G. rufa* should be considered as different species. A recent study suggested that fish described as *G. rufa* from the springs and streams northern to the Dead Sea in Jordan, are in fact a separate species described as *G. jordanica* (Hamidan *et al.*, 2014). As found here, Israeli fish defined as *G.rufa* are genetically more similar to BOLD *G. jordanica* than to *G.rufa* from Asian countries. K2P distances between Israeli *G. rufa* haplotypes and BOLD *G. jordanica* haplotype range between 0.0018-0.0149, falling well inside the within-species range. Additionally, in the phylogentic tree *G. jordanica* haplotype clustered together with haplotypes of *G. rufa* from Israel, consistent also with their geographic proximity. Thus, ~~according to these results,~~given the distance from Asian *G. rufa* and proximity to *G. jordanica*, a change in species definition ~~of~~ for *G. rufa* fish from Israel from *G. rufa* to *G. jordanica* should be ~~revisited~~considered. This was also suggested theoretically by (Hamidan *et al.*, 2014) based on the geographic distribution data, and is now supported by barcoding evidence. The analyses of discrepancies between morphological and molecular species definition, primarily that of the *Garra* ~~*spp.*~~ Complex, are ~~that was analyzed here in more details is~~

~~just one~~ examples for how DNA barcoding can assist in resolving and revisiting taxonomic and ecological questions.

~~The sampling scheme employed for this study allowed for the first time to develop and apply molecular genetics tools to many of the freshwater fish species of Israel in the context of their natural habitats.~~ The utility of species identification using DNA barcoding in the context of ecological research was extensively discussed in the last decade and new implementations of DNA barcoding are being published ever since this method was standardized (Andrew *et al.*, 2013; Blanchet, 2012; Hajibabaei *et al.*, 2007; Hughes *et al.*, 2014). The sampling scheme employed for this study allowed for the first time to develop and apply molecular genetics tools to many of the freshwater fish species of Israel in the context of their natural habitats. Studies are starting to employ molecular tools relying on or assisted by molecular species identification to address ecological questions regarding native lake fish populations such as the spatial and temporal distribution and abundance of cichlid fry along lake shores (Cummings et al. 2017) and decline of genetic variation in the *S. galilaeus* lake population (Borovski et al. 2018). ~~In addition to the insights gained by the various analyses, the DNA barcodes database developed here, could serve as the basis for developing valuable tools for monitoring fish populations and to further study ecological questions regarding these fish.~~

Conclusions

This study established a molecular DNA barcoding database for native and non-native fish species inhabiting the Sea of Galilee. The data obtained here exemplified how in combination with the public BOLD database, analyses of geographic patterns,

Commented [LD4]: Cummings, D., Goren, M., Gasith, A., & Zohary, T. (2017). Inundated shore vegetation as habitat for cichlids breeding in a lake subjected to extreme water level fluctuations. *Inland Waters*, *7*(4), 449-460.
Borovski, T., Tadmor-Levi, R., Shapiro, J., Rubinstein, G., Agyakwah, S. K., Hulata, G., & David, L. (2018). Historical and recent reductions in genetic variation of the Sarotherodon galilaeus population in the Sea of Galilee. *Conservation Genetics*, *19*(6), 1323-1333.

morphological and molecular taxonomy can address issues in species definition on one hand, and on the other, how the molecular data could be used as a basis to address ecological question concerning lake species. Developing modern, molecular tools for identification of fish down to species level and further, are being valuable tools for monitoring fish populations and supporting management and conservation decisions, in such key freshwater bodies as the Sea of Galilee.

Acknowledgments

The authors wish to thank all who assisted in sampling of fish: ~~Dr. Daniel Golani (The Hebrew University of Jerusalem),~~ David Cummings (Kinneret Limnological Laboratory, IOLR), Dr. Noam Leader, Dr. Dana Milstein, Dr. Amit Dolev and ~~Gizel~~ Gisele Hazzan (Israel National Parks Authority), Menachem Lev (Kibbutz Ein Gev fishing vessel) and colleagues from the laboratory and the Animal Sciences Department. LD is chair of the Vigevani Senior Lectureship in Animal Sciences.

Supporting information captions

Supplementary table S1. *Garra* ~~*spp.*~~ species accessions and geographic information for 16 sample sequences from this study and 33 BOLD accessions.

References

Andrew RL, Bernatchez L, Bonin A*, et al.* (2013) A road map for molecular ecology. *Molecular ecology* **22**, 2605-2626.

Blanchet S (2012) The use of molecular tools in invasion biology: an emphasis on freshwater ecosystems. *Fisheries Management and Ecology* **19**, 120-132.

Borovski T, Tadmor-Levi R, Shapiro J, *et al.* (2018) Historical and recent reductions in genetic variation of the Sarotherodon galilaeus population in the Sea of Galilee. *Conservation Genetics* **19**, 1323-1333.
Casal CMV (2006) Global documentation of fish introductions: the growing crisis and recommendations for action. *Biological invasions* **8**, 3-11.
Darwall WR, Freyhof J (2016) Lost fishes, who is counting? The extent of the threat to freshwater fish biodiversity. *Conservation of freshwater fishes*, 1-36.
Dudgeon D, Arthington AH, Gessner MO, *et al.* (2006) Freshwater biodiversity: importance, threats, status and conservation challenges. *Biological reviews* **81**, 163-182.
Dynesius M, Nilsson C (1994) Fragmentation and flow regulation of river systems in the northern third of the world. *Science* **266**, 753-762.
Ekrem T, Willassen E, Stur E (2007) A comprehensive DNA sequence library is essential for identification with DNA barcodes. *Molecular phylogenetics and evolution* **43**, 530-542.
Golani D, Sonin O, Snovsky G, David L, Tadmor-Levi R (2019) The occurrence of the peacock bass (Cichla kelberi Kullander and Ferreira 2006) in Lake Kinneret (Sea of Galilee), Israel. *BioInvasions Records* **8**, 706-711.
Gophen M, Sonin O, Lev M, Snovsky G (2018) Regulated fishery is beneficial for the sustainability of fish population in Lake Kinneret (Israel). *Ecological Research in the Lake Kinneret and Hula Valley (Israel) Ecosystems*, 233.
Goren M (1973) The cyprinid fishes of Acanthobrama Heckel and related genera.
Goren M (2014) Tristramella sacra. The IUCN Red List of Threatened Species, p. e.T61372A19010617.
Goren M, Ortal R (1999) Biogeography, diversity and conservation of the inland water fish communities in Israel. *Biological Conservation* **89**, 1-9.
Hajibabaei M, Singer GA, Hebert PD, Hickey DA (2007) DNA barcoding: how it complements taxonomy, molecular phylogenetics and population genetics. *TRENDS in Genetics* **23**, 167-172.
Hall TA (1999) BioEdit: a user-friendly biological sequence alignment editor and analysis program for Windows 95/98/NT **41**, 95-98.
Hamidan NA, Geiger MF, Freyhof J (2014) Garra jordanica, a new species from the Dead Sea basin with remarks on the relationship of G. ghorensis, G. tibanica and G. rufa (Teleostei: Cyprinidae). *Ichthyological Exploration of Freshwaters* **25**, 223-236.
Hamidan NA, Mir S (2003) The status of Garra ghorensis in Jordan: distribution, ecology and threats. *Zoology in the Middle East* **30**, 49-54.
Havel JE, Kovalenko KE, Thomaz SM, Amalfitano S, Kats LB (2015) Aquatic invasive species: challenges for the future. *Hydrobiologia* **750**, 147-170.
Hebert PD, Cywinska A, Ball SL, Dewaard JR (2003) Biological identifications through DNA barcodes. *Proceedings of the Royal Society of London. Series B: Biological Sciences* **270**, 313-321.
Hubert N, Hanner R, Holm E, *et al.* (2008) Identifying Canadian freshwater fishes through DNA barcodes. *PLoS one* **3**, e2490.
Hughes JM, Finn DS, Monaghan MT, Schultheis A, Sweeney BW (2014) Basic and applied uses of molecular approaches in freshwater ecology. *Freshwater Science* **33**, 168-171.
Kantoussan J, Ecoutin JM, Fontenelle G, de Morais LT, Laë R (2014) Catch per Unit Effort and yields as indicators of exploited fish communities: application to two West A frican reservoirs. *Lakes & Reservoirs: Research & Management* **19**, 86-97.
Lemoalle J, de Condappa D (2010) Farming systems and food production in the Volta Basin. *Water International* **35**, 655-680.

Leprieur F, Beauchard O, Blanchet S, Oberdorff T, Brosse S (2008) Fish invasions in the world's river systems: when natural processes are blurred by human activities. *PLoS Biol* **6**, e28.

Levêque C, Oberdorff T, PAUGy D, Stiassny M, Tedesco PA (2007) Global diversity of fish (Pisces) in freshwater. In: *Freshwater animal diversity assessment*, pp. 545-567. Springer.

Lind CE, Brummett RE, Ponzoni RW (2012) Exploitation and conservation of fish genetic resources in Africa: issues and priorities for aquaculture development and research. *Reviews in Aquaculture* **4**, 125-141.

Markel D, Shamir U, Green P (2014) Operational management of Lake Kinneret and its watershed. In: *Lake Kinneret*, pp. 541-560. Springer.

Martínez G, Shaw EM, Carrillo M, Zanuy S (1998) Protein salting-out method applied to genomic DNA isolation from fish whole blood. *Biotechniques* **24**, 238-239.

Nelson J (2006) Fishes of the world John Wiley and Sons. *Inc., Hoboken, New Jersey*.

Nilsson C, Reidy CA, Dynesius M, Revenga C (2005) Fragmentation and flow regulation of the world's large river systems. *Science* **308**, 405-408.

Ostrovsky I, Goren M, Shapiro J, Snovsky G, Rynskiy A (2014a) Fish biology and ecology. In: *Lake Kinneret*, pp. 273-292. Springer.

Ostrovsky I, Zohary T, Shapiro J, Snovsky G, Markel D (2014b) Fisheries management. In: *Lake Kinneret*, pp. 635-653. Springer.

Pont D, Rocle M, Valentini A*, et al.* (2018) Environmental DNA reveals quantitative patterns of fish biodiversity in large rivers despite its downstream transportation. *Scientific reports* **8**, 1-13.

Ratnasingham S, Hebert PD (2007) BOLD: The Barcode of Life Data System (http://www. barcodinglife. org). *Molecular ecology notes* **7**, 355-364.

Reid AJ, Carlson AK, Creed IF*, et al.* (2019) Emerging threats and persistent conservation challenges for freshwater biodiversity. *Biological Reviews* **94**, 849-873.

Ribeiro F, Leunda P (2012) Non-native fish impacts on Mediterranean freshwater ecosystems: current knowledge and research needs. *Fisheries Management and Ecology* **19**, 142-156.

Sala OE, Chapin FS, Armesto JJ*, et al.* (2000) Global biodiversity scenarios for the year 2100. *science* **287**, 1770-1774.

Serruya C (2012) *Lake kinneret* Springer Science & Business Media.

Shirak A, Cohen-Zinder M, Barroso RM*, et al.* (2009) DNA barcoding of Israeli indigenous and introduced cichlids.

Tamura K, Stecher G, Peterson D, Filipski A, Kumar S (2013) MEGA6: Molecular Evolutionary Genetics Analysis Version 6.0. *Molecular Biology and Evolution* **30**, 2725.

Thomaz SM, Kovalenko KE, Havel JE, Kats LB (2015) Aquatic invasive species: general trends in the literature and introduction to the special issue. *Hydrobiologia* **746**, 1-12.

Ushio M, Murakami H, Masuda R*, et al.* (2018) Quantitative monitoring of multispecies fish environmental DNA using high-throughput sequencing. *Metabarcoding and Metagenomics* **2**, e23297.

Ward RD, Hanner R, Hebert PD (2009) The campaign to DNA barcode all fishes, FISH-BOL. *Journal of fish biology* **74**, 329-356.

Ward RD, Zemlak TS, Innes BH, Last PR, Hebert PD (2005) DNA barcoding Australia's fish species. *Philosophical Transactions of the Royal Society B: Biological Sciences* **360**, 1847-1857.

Weigand H, Beermann AJ, Čiampor F*, et al.* (2019) DNA barcode reference libraries for the monitoring of aquatic biota in Europe: Gap-analysis and recommendations for future work. *Science of the Total Environment* **678**, 499-524.

Response to reviewers comments: Pone-D_20_39138
Establishing and using a genetic database for resolving identification of fish species in the Sea of Galilee, Israel

The authors would like to thank the editor and reviewers for their constructive detailed comments. We followed these comments one by one revising the text accordingly. It is evident from this letter of response and also from the very busy 'track changes' version. Following these useful suggestions, we now feel that the revised version is a significant improvement over the initial submission to the level ready for publication. Bellow is a point-by-point response to the specific comments.

Response to editor's points:
1. We went over the manuscript and accompanied files to ensure they meet PLOS ONE style requirements. The revised files are submitted.
2. We would like our manuscript to be considered for the Freshwater Ecosystems collection.
3. We publicly released the repository data as stated in the Data Availability section.
4. Figure 2 was edited and provided at higher resolution. Additionally, we added a copyright statement in figure 2 legend.

Reviewer #1
Comment: This paper describes very well the DNA barcoding of the freshwater fish found in Israel (mainly the Sea of Galileea). All required analyses were well done and I only found two digitation mistakes in species names: line 304 ghorensis, and line 353: Garra (italic). I also do not think that the authors should use names as Garra spp. because this ssp. means species of this genus and when we cite the genus we are already talking about its species. Response: We appreciate the recommendation. The two digitations mistakes were fixed and spp. was deleted from the text.

Reviewer #2:
Abstract
The authors summarize the main research questions and key findings very well. Using the abstract as a foundation, the introduction and discussion should highlight these points in order to strengthen them.
Response: Accordingly, we almost did not change the abstract, except for a few sentences to emphasizing the points recommended by the reviewer in the discussion section.

Introduction
DNA barcoding was used to establish a database of molecular species identification based on sequences of CO1 gene for the Sea of Galilee, Israel. Applied DNA barcoding to samples taken mainly from the Sea of Galilee to address taxonomic questions and build a reference barcoding database for molecular identification of fish in the context of the Sea of Galilee and the region.
Comment: The study should more explicitly indicate that it is not building a new database but rather adding to an existing database such as BOLD. More information of the BOLD

database should be included in the introduction as well as the standardised rules that apply to DNA barcoding. The study fails to relate to previous research in this area. The authors should include more examples, information and scenarios as well as reference the related literature, especially by including more recently published work. Hence, a stronger link between the literature and the topic of this manuscript is required. In the materials and methods section, you mention that methods were adopted from Borovski et al. (2018), however, no mention of this manuscript was included in the Introduction and Lines 71-74 were directly taken from it with no changes. The main point of the study which is to identify fish species from the Sea of Galilee and surrounding regions using DNA barcoding to build a reference database for effective management/conservation does not come across strongly. In other words, the exact reason for this study other than building a database is not explicitly clear, is it for the management of invasive species, monitoring of native species? The link to this being mentioned earlier in the introduction needs to be made clearer towards the end of the introduction.
Response: see below a point-by-point response.

Corrections:
Comment: L50-L51: Remove sentence.
Response: Sentence was removed and replaced by the suggested one.

Comments: L55: Please add a reference for the last bit of this sentence and change the sentence to read as follows, "Despite freshwater habitats only comprising of 1% of the Earth's surface water, they support 43% (13 000 species) of the world's freshwater fish [2,5], rich in biodiversity across all trophic levels [reference, e.g. Vörösmarty et al., 2010; Carpenter et al., 2011]".
Response: sentence was changed as suggested.

Comment: L55-L57: Remove this sentence, as the content has been incorporated in the previous sentence at the beginning of line 55. Also, according to Leveque et al. (2007), only freshwater species and peripheral species add up to 13 000 and 15 000 if brackish water fish are included. According to the authors for this review, "15 000 freshwater fish species are known around the world". This is incorrect, please change the sentence to read either as "13 000 freshwater fish species" or "15 000 species occurring from fresh to brackish waters".
Response: 13,000 was used.

Comment: L51-L54: Move to the end of the sentence of L55 to read as follows: "Despite freshwater habitats only comprising of 1% of the Earth's surface water, they support 43% (13 000 species) of the world's freshwater fish [2,5], rich in biodiversity across all trophic levels [e.g. Vörösmarty et al., 2010; Carpenter et al., 2011]. As a result of human dependence on water, either for direct uses like irrigation and drinking or indirect uses like hydro-electrical energy production and food fishing, many freshwater fish species have become highly endangered [1, 6-9]" L57-L59: Remove sentence.

Responses: lines 51-59 were re-arranged and revised as recommended by the reviewer. References were added as requested.

Comment: L60-L62: Include a reference and change sentence to read as follows: "Effective management for the assessment of freshwater fish biodiversity and population status is therefore a priority, however, can be considered a great challenge.
Response: Text was revised as suggested, reference for this sentence was included.

Comment: L62-L65: I would not include reference number 8 (Lind et al., 2012) and number 10 (Leprieur et al., 2008) in your in-text citation for this sentence. Reasons being, the focus is still on a global point of view and you only mention fish invasions in the next sentence (L65-L66).
Response: The reference to Leprieur et al., 2008 was removed, however the reference to Lind et al., 2012 was kept as it does not refer specifically to fish invasions and is more general.

Comment: L65-L66: Change sentence to read as follows: "Further human derived pressure on natural fish diversity has also been documented due to introduction and invasion of exotic fish species". Remove reference 12 (Goren and Ortal, 1999) and 14 (Ribeiro and Leunda, 2012) in your in-text citation, as again you are focusing on a global point of view here and focus specifically on Israel and the Mediterranean freshwater ecosystems in the next paragraph.
Response: Text was revised, and selected references were removed as suggested.

Comment: L67-L68: Insert the word "arid" before "climatic conditions.
Response: added.

Comments: L69-L77:
Comment: Insert the catchment area size in brackets to read as follows: "biggest surface freshwater reservoir in the middle east (ca. 170 km^2)".
Response: Catchment area size was added in brackets.

Comment: Change "are highly regulated" to "it is highly regulated".
Response: Text was changed as suggested.

Comment: Include reference 12 (Goren and Ortal, 1999) in the in-text citation "[16,17]".
Response: Reference was included.

Comment: L71: Instead of using the term "exotic" I would opt for using "non-native". Also insert the word "freshwater" before "fish"
Response: Comment was accepted, and text changed accordingly.

Comment: L72: Remove reference 12 and 18 from in-text citation as this sentence is more specifically mentioned in reference 19.

Response: Requested references were removed.

Comment: L72-L74: Include references for this sentence.
Response: A reference for this sentence was added

Comment: L74: When including the common and scientific name for the first time, first write out the common name followed by the scientific name with the original author/identifier in brackets. Change to read as follows: “The native cyprinid Kinneret bleak *Mirogrex terraesanctae* (Steinitz, 1952)”.
Response: Changed here and throughout text that species are first mentioned properly. According to taxonomy conventions, the original identifier of the species was written in brackets when genus/species name were changed since first identified but without brackets when the original identification name was kept to date.

Comment: L75-L76: Change sentence to read as follows: “From the native cichlids Galilee tilapia or St. Peter’s fish *Sarotherodon galilaeus* (Linnaeus, 1758) is the most abundant”. Also can you specify how they are abundant? By number or biomass as was done in the previous sentence for the cyprinid.
Response: Text was revised as follows: “Based on fishery landings, the native cichlid, Galilee tilapia, or St. Peter’s fish *Sarotherodon galilaeus* (Linnaeus, 1758), is abundant, most valuable and sought after."

Comment: L77: Scientific names are predominantly used over common names once the species has been introduced. Also, *S. galilaeus* African distribution is more centralised and western, therefore, please change sentence to read as follows: “While *M. terraesanctae* is endemic to Israel, *S. galilaeus* is widespread in central and western Africa”. Please remove reference 21 (Lemoalle and de Condappa, 2010) from the in-text citation for this sentence, as this reference does not refer to *S. galilaeus* African distribution which is of focus here but rather to the Volta basin (west coast of Africa).
Response: Text was changed as suggested and checked that scientific rather than common names are used throughout manuscript. Reference 21 was removed.

Comments L78-L82:
Comment: Please rephrase sentence to read as follows: “Non-native species, which were unintentionally introduced, include the European eel *Anguilla* (Linnaeus, 1759) that hitchhiked with mullet fry caught in coastal estuaries and stocked into the lake, the hybrid tilapia *Oreochromis niloticus* (Linnaeus, 1758) x *Oreochromis aureus* (Steindachner, 1864) that escaped from aquaculture ponds and the peacock bass *Cichla kelberi* (Kullander and Ferreira, 2006) that was probably released in the lake by hobbyists [22]”.
Also, please include references for the introductions of the European eel and the hybrid tilapia in the in-text citation as currently you only have a reference for the peacock bass.
Response: Text was updated by relevant references and revised.

Comments L82-L83:

Comment: Here you listed *Gambusia affinis* as “exotic” stipulating that it is not harmful to native species, however, this is not the case as *G. affinis* is listed as one of the world’s worst non-native invasive species (Lowe et al., 2000). Please rephrase this sentence to read as follows: “Other non-native species, which were intentionally introduced and invasive include the mosquito fish *Gambusia affinis* (Baird and Girard, 1853) which were introduced to control mosquitoes. Please also include a reference for this sentence.
Response: The sentence was revised. A reference for the introduction of *Gambusia* fish was added
Comments L83-L91: Please rephrase this sentence to read as follows: “Nevertheless, significant non-native species which were introduced to enhance commercial fishing included the common carp *Cyprinus carpio* (Linnaeus, 1758), the silver carp *Hypophthalmichthys molitrix* (Valenciennes, 1844), the thin lip grey mullet *Chelon ramada* (Risso, 1827) and the flathead grey mullet *Mugil cephalus* (Linnaeus, 1758)”.
Response: Text was revised as suggested.

Comment: Please note that the genus name “Liza” has been updated to “Chelon”. For assurity, please check that all species mentioned in this paper are up to date by reviewing them in the Catalogue of Fishes Database.
Response: We checked all species names mentioned in this manuscript by reviewing them in the Catalogue of Fishes Database, to our knowledge all species names are currently up to date.

Comment: Also, please include a reference for this sentence.
Response: References for introduction of non-native species mentioned here were added.

Comment: L86-L88: Please include a reference for this sentence.
Response: A reference for this sentence was added.

Comment: L90-L91: Change “estimate” to “estimates” and change “Kinneret bleak” to “*M. terraesanctae*”.
Response: Text was changed as suggested.

Comment: L93-L94: Rephrase sentence to read as follows: “In recent years, DNA polymorphism, a more reliable and accurate method, is increasingly used for taxonomic and ecological research”.
Response: Comment was accepted, and text changed.

Comment: Please include a references for this sentence by providing other examples of past literature where DNA polymorphism has been used successfully.
Response: A few example references were added to text.

Comment: L94: Change “sequence” to “sequences”.
Response: Comment was accepted, and text changed.

Comment: L95: Remove "species" before "DNA barcoding".
Response: Comment was accepted, and text changed.

Comment: L98: After "environmental water samples, include "known as E-barcoding".
Response: Comment was accepted, and text changed.

Comment: L98-L99: Elaborate more on what reference databases have been used and are currently being used successfully, e.g. BOLD. Explain the process in more detail. This will strengthen your argument and lead nicely on to your aim for the study.
Response: The process of molecular identification of specimen was explained in more detail, and the Barcode of Life Data systems was presented and described shortly.

Comment: L101: Change "question" to "questions".
Response: Comment was accepted, and text changed.

Methods
The experiments or intervention used was DNA barcoding. DNA barcoding was appropriate for addressing the research question. However, the data collected was not interpreted accurately. I found it very hard to follow the protocols and feel that another researcher would battle to reproduce this study based on the methodology reported. For instance, sampling methods are not clear, number of samples collected for each species per site are not clear as well as how many successful sequences were used from this sample size. No mention of whether the fish collected were stored in formalin for taxonomic identification as well as no mention of photos of samples for each species were taken to upload with BOLD. References were not provided or corresponded to the authors point. Data analysis section needs work. Please could the authors rectify the above and include them into the materials and methods section.
Response: see below a point-by-point response.

Comment: L106-L108: Firstly, I would not recommend saying "in the context", rather say "from the Sea of Galilee and surrounding areas". Secondly, what is this extensive sampling scheme that was carried out to obtain samples from each species that could be found in the Lake? For instance, how were these fish collected? Electrofishing? Seine netting? It is important to incorporate this detail here in the beginning of your materials and methods section as well as the years in which this was done (2014 and 2015?) to correspond to your permit numbers.
Response: Text was changed as suggested. Details regarding the sampling scheme are explained in more detail in the following lines as suggested and addressed in the next comment. Information on the permits for sampling was added in the ethical approval section.

Comments: L108-L113: What do you mean by "12 sampling attempts" and "few additional attempts"? This gives me the impression that collection was done on 12 separate occasions but nothing was collected by using the word ''attempts". Also, Borovski et al. (2018), does

not mention anything about “12 sampling attempts” but rather provides more detail in a table and figure of the location of the sites sampled from. I would recommend changing this sentence to read as follows: “The Sea of Galilee, the main sampling site, and surrounding sites such as Asi stream (one of Beit She’an streams, south to the lake and part of the eastern watershed of Israel), Ein Afek pools (close to Acre, i.e. Akko, part of the western watershed of Israel) and Ein Te’o spring (close to Hula Lake, northern to Lake of Galilee and part of the eastern watershed of Israel) (Fig. 1) was sampled during 2014 and 2015 to collect samples of species by means of “seine netting” (whichever method you used for collection) that predominantly occur in this region of Israel for DNA barcoding. Numbers of individual samples collected for each site and species are recorded in Table 1”.
I would also recommend including a map (Fig.1) of your sites including all the small town names and streams as mentioned above in brackets on the map for a point of reference and in Table 1 include columns labelled “site” (Sea of Galilee/Kinneret, Asi stream, Ein Afek pools, and Ein Te’o spring) and “sample size” indicating the number of samples collected for each species for each site.
Response: Sampling was done on 12 different occasions, all ‘attempts’ yielded fish for sampling. Details about sampling method, locations and yields were explained in more detail based on the recommendation for text revision given here. Additionally, a new Table 1 was added to provide details about number of fish sampled compared to number successfully barcoded from each species and from each location. Instead of providing a map as suggested we refer to figure 1 in Borovski et al. (2018) were this data was already published, as we would like to avoid publishing the same data twice. We however, modified the map in Figure 2 to include sampling site names so this information is readily available in this manuscript as well.

Comment: Within table 1 change the column “N samples” to “N samples barcoded”. This will give us a clear indication of the number of samples per species per site that were successfully barcoded from the sample size collected for each species.
Response: Within the table (now table 2) ‘N samples’ was changed to ‘N barcoded samples’.

Comment: Be consistent of your labelling and mentioning of Sea of Galilee rather than “Lake Kinneret” as this was in brackets in the introduction section (L69).
Response: Text was reviewed throughout to ensure that ‘Sea of Galilee’ is used rather than ‘Lake Kinneret’.

Comment: L113-L114: When referring to fish being taxonomically identified to species level, were they identified in the field and returned to the environment or where they sacrificed and taken for identification under lab conditions (preserved entire sample in formalin)? Please specify in this sentence. Also, under DNA barcoding methods of collection, it is important to photograph each sample and upload this photograph on the BOLD database with it its barcode index number (BIN number), was this done? If so, please include this information. Furthermore, why specifically fin clips and not a muscle tissue sample? According to Weight et al. (2012) (DNA barcoding fishes, a good reference to include here), even though fin clips work, the most effective material is a small muscle biopsy from the

specimen taken from the right side of the body. Please explain in detail why a fin clip over muscle tissue was done. Lastly, if fin clips were taken, was there evidence of the fish surviving if returned to its habitat and not preserved? Were there any infections caused which may have made the individual more susceptible to predation?
Response: Text was revised to include more information about sampling procedures and fish handling. Text is now informative with regards to all these points. In most cases fish were returned to their environment after identification in the field and sampling of a fin clip. We have experience with fish (including critically endangered fish) in lab conditions showing that after recovery from anaesthesia, fish return to normal behaviour and their survival is mostly unaffected. As fins easily regenerate and fish integument is unharmed, fin clip samples were chosen over muscle and in our experience, fin clips prove to be effective for DNA isolation and barcoding. Some of the fish were sacrificed photographed and deposited in the National Natural History Collections of the Hebrew University, in the new Table 1, the information of number of specimens that were deposited and their Museum catalogue numbers was added. This data was also made available in BOLD.

Comment L118: Change to read as follows: “DNA extraction, polymerase chain reaction (PCR), purification and sequencing”. Combine your original sub-headings of “DNA extraction” and “PCR products and sequencing of PCR products” into one paragraph.
Response: The two sub-sections were combined under the sub-heading of “DNA extraction, polymerase chain reaction (PCR), purification and sequencing”.
Response: done as suggested.

Comment L119-L120: For the salting out method, a reference, Martinez et al. (1998) is provided, however, this citation refers to the salting out method from fish whole blood. Ideally, it would be better to provide a reference that directly links this method for extracting DNA from fin clips of fish as was stipulated, for example Taggart et al. (1992) or Sunnucks and Hales (1996) which in most cases for other references, related to this method, modified it from Miller et al. (1988).
Response: A reference for the salting out method from fin samples was added.

Comment L120-L126: Delete. Not necessary to go into detail if you provide the correct reference for the salting out method and protocol in which you followed.
Response: These lines were deleted.

Comment L132-L135: The primers indicated in the manuscript are not the same primers used as Ward et al. (2005). They used FishF1, FishF2, FishR1, FishR2 and the primers included in the manuscript were coxla_fw, coxla_rv. Hence, please do not indicate that they were “adopted” from Ward et al. (2005) but rather provide a reference that used the same primers as was indicated in this manuscript. Change sentence to read as follows: “A partial fragment of (include the number of base pairs here) of the mitochondrial cytochrome c oxidase subunit 1 gene (COI) was amplified by PCR using the primers …….”.
Response: We used primers FishF1 and FishR1 developed by Ward et al. (2005) under the names cox1a_fw and rv, which were internal names used in our lab. The sequence,

however, is identical. We therefore changed the primer names in the text to match the original names from Ward et al. Additionally text was revised according to the comment.

Comment L138: Please include the amount of water used to complete the 20μl reaction volume and change "PCR profile" to "PCR thermal protocol".
Response: We added the amount of water used in our PCR protocol to complete to 20 μl, and changed "PCR profile" to "PCR thermal protocol" as suggested.
Comment L139-L142: Please include the reference that you used this protocol from or modified it from.
Response: We developed a thermal protocol in our lab that was effective for amplification of products by these primers and described it in the text, therefore no reference was provided.

Comment L144-L146: Change sentence to read as follows: "Successful PCR products were purified using an Exonuclease I-Shrimp Alkaline Phosphate (ExoSAP-IT) method (USB, Cleveland, OH) and sequenced using BigDye V3.1 (add manufacturers name) terminator chemistry in the forward and reverse direction on an ABI PRISM 3730xl DNA analyser (add manufacturers name) following the manufacturers protocol".
Response: Text was revised as suggested and manufacturers of materials and instruments were provided where they were missing.

Comments: L148:L159
For the data analysis section, methodology processes are not clearly explained and a lot more work needs to be done here. For instance, under this section it needs to be mentioned that successful sequences for each species was deposited into the Barcode of Life Data (BOLD) System (Ratnasingham and Hebert, 2007; https://www.boldsystems.org) under the project name LKCOX (remove this sentence from results section under molecular barcoding, and add it under data analysis).
Response: Text was revised as requested, the relevant sentence was moved here from the results section, and blended with the existing revised text.

Comment: In addition, once deposited, what exactly was the role of the BOLD database? Were the sequences from this study used to compare against available reference database sequences to find the closest/nearest matching species which was displayed as a percentage probability (0-100%)? Also, for each species, what was the number of sequences submitted into BOLD for comparison (is this the N samples column in Table 1)? Next, were the publicly available sequences with a >98% similarity/match downloaded from the BOLD reference database (and how many) to include with your sequences in running the haplotype analysis. In the results section, L196-L197 includes the above information, move this sentence here to the data analysis section. Also, L204-L206 of the results section needs to be moved here to the data analysis section.
Response: This section was rearranged, including the requested lines from the results section, as suggested. Now, the text includes all requested information. Regarding the

number of sequences submitted for each species – this is described in Table 1 and referred to here.

Comment: How did you run the haplotype (phylogenetic) analysis? How did you get the relative number of haplotypes for each species? Did you use the software DnaSP? Response: After alignment of sequences, we manually inspected polymorphisms and named polymorphic sequences of the same species as different haplotypes. This is now stated more clearly in the text.

Comment: Was the neighbour joining tree rooted or unrooted for both Figure 1 and Figure 2? Please provide a reference for the bootstrap method of 1000 replicates.
Response: Trees in both figures were unrooted, this is now stated in the methods section and also in both figure legends. A reference for the bootstrap method was provided.

Comment: How was average pairwise haplotype distances calculated? Did you use ARLEQUIN software?
Response: The average pairwise haplotype distances was calculated by summing up all relevant pairwise K2P distances, calculated using MEGA software (described in the text), and dividing them by the number of comparisons.

Comment: Please stipulate what version of the BioEdit software was used and indicate what the total length of base pairs was used or trimmed to by the software.
Response: This data is now added in the relevant places in the text.

Comment: Considering relevant species were sampled from four sites (mainly the Sea of Galilee), why was a haplotype network not included to show if some species indeed differed according to site based on their haplotype groupings?
Response: This suggestion applies mainly to the Garra complex, for which we analyzed samples from different locations showing different haplotypes. Most species have different haplotypes within the lake population (including Garra), leaving such an analysis less interesting. Nevertheless, this point for Garra, including from other countries, in our opinion, is presented in a more specific and legible way than a haplotype network in Figure 2.

Results

Results are very interesting! Figures and tables are clear and readable, however Figure 2 is not of great quality and more information needs to be incorporated in to the map (Fig 2A; see below corrections for further recommendations). Table 2 legend should be above the table and not below it.
Response: see below a point-by-point response.

Corrections:

Comment: I would recommend for Figure 1 to exclude the K2P distances combined here and provide them as separate tables as has been done for Table 2, just separate it according to family using sub-headings.
Response: We prefer to leave the K2P distances within the figure, instead of separating them in three tables. Separate tables will be small and require scrolling among them and back in reference to Fig. 1, which, we think, will be less convenient to the reader. This way the distances and phylogeny can be viewed simultaneously to demonstrate better the points of interest.

Comment: Also correct acronyms for Figure 2 and Table 2 (see below corrections for further recommendations).
Response: This was corrected as per the below comments.

Comment: L169-L170: Delete.
Response: Deleted.

Comment: L171: Are there 25 fish species reported in the Lake? From the in text references reported for this sentence, Goren and Ortal (1999) reported 19 native species in the Lake; Ostrovsky et al. (2014) reported 19 native species in the lake; and I did not come across the number of native species for Ostrovsky et al. (2014) fisheries management chapter. Even Borovski et al. (2018) reports 19 native species and 8 exotic species, which gives a total of 27 and not 25. Please clarify.
Response: In Ostrovsky et al. (2014) there are 18 native species in the lake (19 stated, probably after Goren and Ortal, however, in the table actually detailing the lake species (table 16.1 in Ostrovsky et al., 2014), only 18 species are listed). In comparison to Goren and Ortal (1999), the missing species is *Nun galilaeus*, which was native to the Hula valley and suspected to have migrated into the lake, but anyways, since the 1950's this species is considered extinct. Therefore, we updated our introduction to report 18 native fish species and changed text here to be more specific by reporting how many of the native species we obtained barcode sequences for, and how many non-native species.

Comment: L172-L176: Please provide the original author/identifier in brackets for all species mentioned in these lines. Change “schemes” to “sites”.
Response: author/identifiers were added to each species as appropriate.

Comment: L177: At the end of the sentence add Table 1 in brackets.
Response: Added.

Comment: L177-L179: Rephrase this sentence to read as follows: “Additionally, 20 sequences were analysed from two fish species, *H. molitrix* (19 sequences) and *O. aureus* (1 sequence), that were suspected as hybrids with aquaculture fish (hereafter referred to as ‘suspected hybrids’) (Table 1)”.
Response: Text was revised as suggested.

Comment: L179-L180: Delete and move to data analysis section under materials and methods.
Response: This sentence was moved to m&m, data analysis section.

Comment: L181-L182: As previously mentioned, can the authors make clear how many specimens were collected for each species, and out of this sample size, how many were successfully sequenced and add this to Table 1 as some sequences may have been unsuccessful. Also, at the end of this sentence add Table 1 in brackets.
Response: The new Table 1 includes the requested information.

Comment: L183-L184: At the end of the sentence add Table 1 in brackets.
Response: Added, but now Table 2.

Comment: L186: Add the "number" of species where new barcodes were added to existing barcodes in BOLD.
Response: The number of species with novel barcodes was already in the sentence, however, to clarify, we changed the sentence to read as follows: "For ten species with barcodes existing in BOLD, 12 new barcodes were added. These added haplotypes were always those with the lower frequency in our data (Table 2)."

Comment: L188-L190: Change Table 1 legend to include sites and sample sizes for each species.
Response: This information is now detailed in the new Table 1.

Comment: L196-L197: Delete and move to data analysis section under materials and methods.
Response: This sentence was deleted here and moved to data analysis section of methods.

Comment: L197-L199: Specify the exact number of species where molecular identification agreed with the original morphological identification.
Response: We added that for 19 out of 22 molecular identification agreed with the original morphological identification.

Comment: L199-L201: Delete and move to data analysis section under materials and methods.
Response: This sentence was deleted here and moved to data analysis section of methods.

Comment: L204-L206: Mention how many species were in disagreement. Please rephrase the sentence to read as follows: "Three species were found to have disagreements between morphological and molecular barcoding identification. Further investigation was carried using all available COI barcode records from BOLD of query and related species".
Response: Text was revised as suggested, and the methods part was moved into data analysis section of methods.

Comment: L206: Cannot start a sentence with the genus name of a scientific name abbreviated, need to write it out in full, "*Acanthobrama*". Delete "we".
Response: Text was revised as suggested.

Comment: L211-L214: Remove from results section and add to discussion section. You are only stating the main points of results and should not discuss them in depth in this section.
Response: Text was revised as suggested.

Comment: L215-L217: Please rephrase the sentence to read as follows: "Three *Gambusia affinis* samples collected, all with the same haplotype (GaA), was identified by BOLD as *G. holbrooki* (100% fit), followed by *G. affinis* (99.8% fit).
Response: Text was revised as suggested.

Comment: L217-L220: Please delete the last part of this sentence and move to discussion section. Please rephrase sentence to read as follows: "Comparison to all available *G. holbrooki* and *G. affinis* records from BOLD indicated that GaA haplotype clusters together with *G. holbrooki* records".
Response: the last part of the sentence was deleted and moved to the discussion section.

Comment: L222: Remove "identification module".
Response: deleted.

Comment: L223-L224: Rephrase this sentence to read as follows: "This disagreement will be discussed further later under its own sub-section".
Response: Text was revised as suggested.

Comment: L225-L226: Rephrase sentence to read as follows: "Molecular identification using BOLD for samples of fish suspected as hybrids provided genetic evidence to support hybridization.
Response: Text was revised as suggested.

Comment: L228: Change "into" to "in".
Response: Text was revised as suggested.

Comment: L229-L231: Delete this sentence, starting from "This is consistent".
Response: Text was revised as suggested.

Comment: L234-L235: Add a full stop after "in both directions" and delete the remainder of the sentence starting from "again".
Response: Text was revised as suggested.

Comment: L236: Change "another" to "further".
Response: Text was revised as suggested.

Comment: L239-L252: For genetic distance from barcoding data, I would start off with discussing the results from the neighbour-joining phylogenetic tree and incorporating the K2P genetic distances with this.
Response: This paragraph begins with a general statement about the scale of the K2P distances, followed by a general description of the phylogenetic tree. In the next paragraph, a more detailed description of the interesting results in the tree are combined with the K2P genetic distances. We believe this is a reasonable and logical way to present the results.

Comment: In figure 1, for certain haplotypes there as asterisk next to them. In the Figure 1 legend, please indicate what these asterisks mean.
Response: Description of the asterisks meanings were added to figure 1 legend.

Comment: L260-L263: Please rephrase this sentence to read as follows: "Unexpected results were, however, found within the Cichlidae clade of the neighbour-joining phylogenetic tree. *Oreochromis* aureus (Haplotype OaA) clustered together with *Sarotherod galilaeus* (Haplotype SgA), a species belonging to a different genus, rather than with *O. niloticus* (Haplotype OaB) with which its genus is shared".
Response: Text was revised as suggested.

Comment: L263-L267: Please rephrase this sentence to read as follows: "Support from K2P distances between *O. aureus* and *S. galilaueus* (0.0095) is smaller in comparison to distances between other cichlids of Lake Galilee belonging to different genera, and even smaller compared to cyprinids belonging to different genera (0.08-0.23) (Fig. 1)".
Response: Text was revised as suggested.

Comment: L267-L268: Delete this sentence here and move it to discussion section.
Response: This suggestion was followed.

Comment: L269: Change "*G. rufa*" to "*Garra rufa*".
Response: Text was revised as suggested.

Comment: L270: delete "we find".
Response: Deleted.

Comment: L271-L273: Please rephrase this sentence to read as follows: "Identical K2P distances was, however, found between *Acanthobrama telavivensis* and *Mirogrex terraesanctae*, formerly known as *A. terraesanctae* and later moved to a separate genus". Reference [36] has been removed here as in the result section you do not include in text references.
Response: This suggestion was followed.

Comment: L273-L274: Please delete this sentence, starting from "This relatively" and move to the discussion section.
Response: This suggestion was followed.

Comment: L277: Delete "As mentioned before". Start sentence with "Comparison of Garra rufa haplotypes".
Response: Text was revised as suggested.

Comment: L281-L282: Delete "Samples identified morphologically as". Start sentence with "Garra rufa fish".
Response: Text was revised as suggested.

Comment: L283: After Ein Te'o put "north of the Sea of Galilee" in brackets.
Response: Text was revised as suggested.

Comment: For Figure 2A, can the authors please explicitly label Asi stream, Ein Afek and Ein Te' o stream in text in the correct location on the map to make these locations clearer in relation to the haplotypes. Also, include labels for eastern and western watersheds of Israel on the map (Fig. 2A). Furthermore, can you give Haplotype GrD its own circle and colour and make GrA haplotype in the Sea of Galilee the same colour as in Ein Te'o (the colours on the map look different).
Response: Figure 2 was edited, site names were included (except for Ein Afeq, since no Garra fish were sampled there), eastern and western watersheds were not marked on the map since all sampled fish are from the eastern watershed of Israel. Haplotype GrD received its own circle and colour and we made sure that GrA haplotype in the Sea of Galilee is the same colour as GrA in Ein Te'o. Furthermore, map resolution was improved.

Comment: L284: Make "the" prior to Sea of Galilee samples capitalized.
Response: Text was revised as suggested.

Comment: L284-L287: Please rephrase this sentence to read as follows: "The Sea of Galilee samples had five fish from Haplotype GrA, two fish from Haplotype GrC and one fish from Haplotype GrD. All three fish from Ein Te'o shared Haplotype GrA and all three fish from Asi stream shared Haplotype GrB (Fig. 2A).
Response: Text was revised as suggested.

Comment: L289-L298: Please rephrase Figure 2 legend to read as follows: "Sampling sites and clustering of Garra spp. Samples based on COI haplotypes. Acronyms of populations are: GrA – *Garra rufa* from Haplotype A; GrB – *Garra rufa* from Haplotype B; GrC – *Garra rufa* from Haplotype C; GrD – *Garra rufa* from Haplotype D; Gg – *Garra ghorensis* from the southern basin of the Dead Sea; Gj – *Garra jordanica* from the northern basin of the Dead Sea; GnA – *Garra nana* from Haplotype A; GnB – *Garra nana* from Haplotype B. (A) Map showing the sampling sites in a regional context. Different *Garra spp.* Haplotypes were denoted by differently coloured circles in each sampling site on the map of Israel, Jordan and Syria regions. (B) A dendrogram constructed for Haplotypes obtained from samples collected in this study alongside Haplotypes mined from BOLD (see Process ID's in Table S1). Next to the edges, haplotype acronyms are given as indicated above. Numbers next to

edges denote percent bootstrap support. Colours of Haplotype text match the colour of the circles on the map (A)".
Response: Text of the figure legend was revised as suggested.

Comment: L300: Delete "To put into context the *Garra spp.* Sampled from Israel,". Start sentence with "Sequences of 16 *Garra spp.* From".
Response: Text was revised as suggested.

Comment: L301: Delete "a" before "geographic location".
Response: Deleted.

Comment: L302: Add "assigned" before "to them" and add "see" before "Table S1" in brackets. Add "neighbour-joining" before "phylogenetic tree (Fig 2B)".
Response: Text was revised as suggested.

Comment: L305: After 0.0381 in brackets, add "; Table 2). After "were found in fish from" add "the Sea of Galilee and a northern location in Syria (Fig 2A). Delete "two isolated locations" and "GnA, the *G. nana* haplotype found in the Sea of Galilee was found also in samples from a northern location in Syria".
Response: These lines were rephrased to read as follows 'The two haplotypes of *G. nana* (GnA and GnB) were relatively distinct from one another (K2P = 0.0381; Table 3). GnA, was found in fish from the Sea of Galilee and the more northern location in Syria, while GnB, was found in fish from an isolated location more southern in Syria (Fig 2A).'.

Comment: L308-L311: Please rephrase this sentence to read as follows: "Israel *G. rufa* samples from the Sea of Galilee and Ein Te'o had Haplotypes GrA, GrC and GrD. These Israel *G. rufa* Haplotypes clustered with BOLD Haplotypes of *G. jordanica* (Haplotype Gj), fish sampled from streams in the northern basin of the Dead Sea in Jordan (Fig. 2B)".
Response: Text was revised as suggested.

Comment: L311-L314: Please rephrase this sentence to read as follows: "Close by, yet separated, in the phylogeny, was *G. rufa* fish, Haplotype GrB, from Asi stream, suggesting again greater similarity to *G. jordanica*, but some genetic separation from the Sea of Galilee population".
Response: Text was revised as suggested.

Comment: L318: Insert "GrAsia –" before "Turkey, Iran and Iraq" in the brackets.
Response: Inserted.

Comment: L318-L320: Delete this sentence starting from "Thus, the results indicate that" and move it to the discussion session.
Response: Deleted and moved.

Comment: L321-L327: Figure legend for Table 2 should go above the table and not below the table. Provide a reason as to why Gr North has an asterisk sign and the other locations do not. Change acronyms for populations to read as follows: "Gr North – *Garra rufa* from Sea of Galilee and Ein Te'o of northern Jordan valley; GrAsi – *Garra rufa* from Asi stream of Beit she'an valley; Gj – *Garra jordanica* from the northern basin of the Dead Sea; Gg – *Garra ghorensis* from the southern basin of the Dead Sea; Gr Asia – *Garra rufa* from Asian countries including Turkey, Iran and Iraq; GnA – *Garra nana* from Haplotype A; GnB – *Garra nana* from Haplotype B".
Response: Figure legend for this table (now Table 3) was placed above the table and revised as suggested.

Comment: L333-L337: Please rephrase this sentence to read as follows: "The Israeli *G. rufa* fish (Gr North) are somewhat genetically divergent from *G. rufa* fish from eastern and norther Asian countries (Gr North vs Gr Asia mean K2P distances = 0.0025 vs 0.0462; Table 2)".
Response: To clarify the point of interest, the text was revised to read as follows: 'The Israeli *G. rufa* fish, are genetically divergent from *G. rufa* fish from farther eastern and northern Asian countries (Mean K2P between Gr North and Gr Asia = 0.0462, and between Gr Asi and Gr Asia = 0.0469; Table 3).". The text following these lines was also revised similarly to improve clarity.

Comment: L340-L341: Delete "from the Northern Jordan valley" and replace it with "(Gr North)".
Response: Text was revised as suggested.

Comment: L343: Insert "south Asi stream of" before "Beit she'an valley". Delete "(Asi stream)" and replace it with "(Gr Asi)".
Response: Text was revised as suggested.

Comment: L344-L345: Delete "in the Hula valley".
Response: Text was revised as suggested.

Comment: L350: insert "(mean K2P of 0.0587) after "from Asia".
Response: Text was revised as suggested.

Comment: L351: Rephrase to read as follows: "*G. rufa* (mean K2P of 0.014) (Table 2)".
Response: Text was revised as suggested, mean K2P should be 0.0414, we assume this was the reviewer's intention.

Discussion

The discussion section is almost a repetition of the authors results section. More evidence needs to be provided here to support your results. Elaborate by including more examples of relevant recent literature to strengthen your arguments. Results are interesting, however a lot more work needs to be done in this section. Also, it is important to include any

limitations that were raised during this this study and should be highlighted and discussed here by providing ways in the future as to how it can be avoided or improved on. Also, there is no conclusion section, which is very important to drive your take home message for this study. For instance, in the conclusion section by adding a how this study could contribute to the conservation of Israel's native species and identification of hybrid and non-native invasive species through DNA barcoding would be a good as well as how it will improve in the future from this perspective.
Furthermore, before discussing the *G. rufa* complex in the discussion, authors need to discuss the other disagreements that were found from your results, namely *A. lissneri* and *A. telavivensis*; *G. holbrooki* and *G. affinis*; hybrids; Cichlidae; and *Acanthobrama-Mirogrex*, in more detail by referring to or by providing recent, relevant and supportive literature. In the results section, I indicated which sentences need to be moved to the discussion section relative to the above sentence as they should not be present in the results section.
Response: the discussion was considerably rewritten incorporating all the suggestions of the reviewer including a conclusions section. Also, see below a point-by-point response to specific comments.

Corrections:
Comment: L361: Reference [25], i.e. Ward et al. (2005) does not directly refer to DNA barcoding as being an important part of BOLD, perhaps rather refer to reference [40].
Response: Sentence was changed to emphasize the utility of fish DNA barcoding including relevant references.

Comment: L361-L362: Please rephrase this sentence to read as follows: "In a previous study conducted by [39], the cichlids of Israel were barcoded with success".
Response: Text was rephrased as suggested.

Comment: L362-L363: Please rephrase this sentence to read as follows: "In this study, focus was given to the fish found in the Sea of Galilee and surrounding water bodies in the region, including species other than cichlids".
Response: Text was rephrased as suggested.

Comment: L367: After "12 species", add "mostly belonging to the Cyprinidae and Cichlidae,".
Response: Text was rephrased as suggested.

Comment: L370: For references [24,40] did both studies focus on similar families as this study? Is this a fair comparison?
Response: References of studies on similar species in which similar distances were found were added.

Comment: L375-L377: Please provide a reference for this sentence.
Response: References for this sentence were added

Comment: L389: Please indicate how recent this isolation occurred in by means of a time frame.
Response: We added the dating of this isolation (~24,000 years ago) to the text, along with a supporting reference.
