# Supplementary material for: Establishing and using a genetic database for resolving identification of fish species in the Sea of Galilee, Israel
Source: PLoS One. 2022 May 19;17(5):e0267021. doi: 10.1371/journal.pone.0267021 (PMC9119452; doi:10.1371/journal.pone.0267021)
Supplement: Attachment — Submitted filename: Review corrections_6 Nov 2021.docx [file pone.0267021.s002.docx]

**Review: Pone-D_20_39138**

**Establishing and using a genetic database for resolving identification of fish species in the Sea of Galilee, Israel**

**Abstract**

The authors summarize the main research questions and key findings very well. Using the abstract as a foundation, the introduction and discussion should highlight these points in order to strengthen them.

**Introduction**

DNA barcoding was used to establish a database of molecular species identification based on sequences of CO1 gene for the Sea of Galilee, Israel. Applied DNA barcoding to samples taken mainly from the Sea of Galilee to address taxonomic questions and build a reference barcoding database for molecular identification of fish in the context of the Sea of Galilee and the region. The study should more explicitly indicate that it is not building a new database but rather adding to an existing database such as BOLD. More information of the BOLD database should be included in the introduction as well as the standardised rules that apply to DNA barcoding. The study fails to relate to previous research in this area. The authors should include more examples, information and scenarios as well as reference the related literature, especially by including more recently published work. Hence, a stronger link between the literature and the topic of this manuscript is required. In the materials and methods section, you mention that methods were adopted from Borovski et al. (2018), however, no mention of this manuscript was included in the Introduction and Lines 71-74 were directly taken from it with no changes. The main point of the study which is to identify fish species from the Sea of Galilee and surrounding regions using DNA barcoding to build a reference database for effective management/conservation does not come across strongly. In other words, the exact reason for this study other than building a database is not explicitly clear, is it for the management of invasive species, monitoring of native species? The link to this being mentioned earlier in the introduction needs to be made clearer towards the end of the introduction.

Corrections

- L50-L51: Remove sentence.
- L55: Please add a reference for the last bit of this sentence and change the sentence to read as follows, “Despite freshwater habitats only comprising of 1% of the Earth’s surface water, they support 43% (13 000 species) of the world’s freshwater fish [2,5], rich in biodiversity across all trophic levels [reference, e.g. Vörösmarty et al., 2010; Carpenter et al., 2011]”.
- L55-L57: Remove this sentence, as the content has been incorporated in the previous sentence at the beginning of line 55. Also, according to Leveque et al. (2007), only freshwater species and peripheral species add up to 13 000 and 15 000 if brackish water fish are included. According to the authors for this review, “15 000 freshwater fish species are known around the world”. This is incorrect, please change the sentence to read either as “13 000 freshwater fish species” or “15 000 species occurring from fresh to brackish waters”.
- L51-L54: Move to the end of the sentence of L55 to read as follows: “Despite freshwater habitats only comprising of 1% of the Earth’s surface water, they support 43% (13 000 species) of the world’s freshwater fish [2,5], rich in biodiversity across all trophic levels [e.g. Vörösmarty et al., 2010; Carpenter et al., 2011]. As a result of human dependence on water, either for direct uses like irrigation and drinking or indirect uses like hydro-electrical energy production and food fishing, many freshwater fish species have become highly endangered [1, 6-9]”
- L57-L59: Remove sentence.
- L60-L62: Include a reference and change sentence to read as follows: “Effective management for the assessment of freshwater fish biodiversity and population status is therefore a priority, however, can be considered a great challenge.
- L62-L65: I would not include reference number 8 (Lind et al., 2012) and number 10 (Leprieur et al., 2008) in your in-text citation for this sentence. Reasons being, the focus is still on a global point of view and you only mention fish invasions in the next sentence (L65-L66).
- L65-L66: Change sentence to read as follows: “Further human derived pressure on natural fish diversity has also been documented due to introduction and invasion of exotic fish species”. Remove reference 12 (Goren and Ortal, 1999) and 14 (Ribeiro and Leunda, 2012) in your in-text citation, as again you are focusing on a global point of view here and focus specifically on Israel and the Mediterranean freshwater ecosystems in the next paragraph.
- L67-L68: Insert the word “arid” before “climatic conditions.
- L69-L71: Insert the catchment area size in brackets to read as follows: “biggest surface freshwater reservoir in the middle east (ca. 170 km^2^)”. Change “are highly regulated” to “it is highly regulated”. Include reference 12 (Goren and Ortal, 1999) in the in-text citation “[16,17]”.
- L71: Instead of using the term “exotic” I would opt for using “non-native”. Also insert the word “freshwater” before “fish”
- L72: Remove reference 12 and 18 from in-text citation as this sentence is more specifically mentioned in reference 19.
- L72-L74: Include references for this sentence.
- L74: When including the common and scientific name for the first time, first write out the common name followed by the scientific name with the original author/identifier in brackets. Change to read as follows: “The native cyprinid Kinneret bleak *Mirogrex terraesanctae* (Steinitz, 1952)”.
- L75-L76: Change sentence to read as follows: “From the native cichlids Galilee tilapia or St. Peter’s fish *Sarotherodon galilaeus* (Linnaeus, 1758) is the most abundant”. Also can you specify how they are abundant? By number or biomass as was done in the previous sentence for the cyprinid.
- L77: Scientific names are predominantly used over common names once the species has been introduced. Also, *S. galilaeus* African distribution is more centralised and western, therefore, please change sentence to read as follows: “While *M. terraesanctae* is endemic to Israel, *S. galilaeus* is widespread in central and western Africa”. Please remove reference 21 (Lemoalle and de Condappa, 2010) from the in-text citation for this sentence, as this reference does not refer to *S. galilaeus* African distribution which is of focus here but rather to the Volta basin (west coast of Africa).
- L78-L82: Please rephrase sentence to read as follows: “Non-native species, which were unintentionally introduced, include the European eel *Anguilla* (Linnaeus, 1759) that hitchhiked with mullet fry caught in coastal estuaries and stocked into the lake, the hybrid tilapia *Oreochromis niloticus* (Linnaeus, 1758) x *Oreochromis aureus* (Steindachner, 1864) that escaped from aquaculture ponds and the peacock bass *Cichla kelberi* (Kullander and Ferreira, 2006) that was probably released in the lake by hobbyists [22]”. Also, please include references for the introductions of the European eel and the hybrid tilapia in the in-text citation as currently you only have a reference for the peacock bass.
- L82-L83: Here you listed *Gambusia affinis* as “exotic” stipulating that it is not harmful to native species, however, this is not the case as *G. affinis* is listed as one of the world’s worst non-native invasive species (Lowe et al., 2000). Please rephrase this sentence to read as follows: “Other non-native species, which were intentionally introduced and invasive include the mosquito fish *Gambusia affinis* (Baird and Girard, 1853) which were introduced to control mosquitoes. Please also include a reference for this sentence.
- L83-L85: Please rephrase this sentence to read as follows: “Nevertheless, significant non-native species which were introduced to enhance commercial fishing included the common carp *Cyprinus carpio* (Linnaeus, 1758), the silver carp *Hypophthalmichthys molitrix* (Valenciennes, 1844), the thin lip grey mullet *Chelon ramada* (Risso, 1827) and the flathead grey mullet *Mugil cephalus* (Linnaeus, 1758)”. Please note that the genus name “Liza” has been updated to “Chelon”. For assurity, please check that all species mentioned in this paper are up to date by reviewing them in the Catalogue of Fishes Database. Also, please include a reference for this sentence.
- L86-L88: Please include a reference for this sentence.
- L90-L91: Change “estimate” to “estimates” and change “Kinneret bleak” to “*M. terraesanctae*”.
- L93-L94: Rephrase sentence to read as follows: “In recent years, DNA polymorphism, a more reliable and accurate method, is increasingly used for taxonomic and ecological research”. Please include a references for this sentence by providing other examples of past literature where DNA polymorphism has been used successfully.
- L94: Change “sequence” to “sequences”.
- L95: Remove “species” before “DNA barcoding”.
- L98: After “environmental water samples, include “known as E-barcoding”.
- L98-L99: Elaborate more on what reference databases have been used and are currently being used successfully, e.g. BOLD. Explain the process in more detail. This will strengthen your argument and lead nicely on to your aim for the study.
- L101: Change “question” to “questions”.

**Methods**

The experiments or intervention used was DNA barcoding. DNA barcoding was appropriate for addressing the research question. However, the data collected was not interpreted accurately. I found it very hard to follow the protocols and feel that another researcher would battle to reproduce this study based on the methodology reported. For instance, sampling methods are not clear, number of samples collected for each species per site are not clear as well as how many successful sequences were used from this sample size. No mention of whether the fish collected were stored in formalin for taxonomic identification as well as no mention of photos of samples for each species were taken to upload with BOLD. References were not provided or corresponded to the authors point. Data analysis section needs work. Please could the authors rectify the above and include them into the materials and methods section

Corrections:

L106-L108: Firstly, I would not recommend saying “in the context”, rather say “from the Sea of Galilee and surrounding areas”. Secondly, what is this extensive sampling scheme that was carried out to obtain samples from each species that could be found in the Lake? For instance, how were these fish collected? Electrofishing? Seine netting? It is important to incorporate this detail here in the beginning of your materials and methods section as well as the years in which this was done (2014 and 2015?) to correspond to your permit numbers.

L108-L113: What do you mean by “12 sampling attempts” and “few additional attempts”? This gives me the impression that collection was done on 12 separate occasions but nothing was collected by using the word ‘’attempts”. Also, Borovski et al. (2018), does not mention anything about “12 sampling attempts” but rather provides more detail in a table and figure of the location of the sites sampled from. I would recommend changing this sentence to read as follows: “The Sea of Galilee, the main sampling site, and surrounding sites such as Asi stream (one of Beit She’an streams, south to the lake and part of the eastern watershed of Israel), Ein Afek pools (close to Acre, i.e. Akko, part of the western watershed of Israel) and Ein Te’o spring (close to Hula Lake, northern to Lake of Galilee and part of the eastern watershed of Israel) (Fig. 1) was sampled during 2014 and 2015 to collect samples of species by means of “seine netting” (whichever method you used for collection) that predominantly occur in this region of Israel for DNA barcoding. Numbers of individual samples collected for each site and species are recorded in Table 1”. I would also recommend including a map (Fig.1) of your sites including all the small town names and streams as mentioned above in brackets on the map for a point of reference and in Table 1 include columns labelled “site” (Sea of Galilee/Kinneret, Asi stream, Ein Afek pools, and Ein Te’o spring) and “sample size” indicating the number of samples collected for each species for each site. Within table 1 change the column “N samples” to “N samples barcoded”. This will give us a clear indication of the number of samples per species per site that were successfully barcoded from the sample size collected for each species. Be consistent of your labelling and mentioning of Sea of Galilee rather than “Lake Kinneret” as this was in brackets in the introduction section (L69).

L113-L114: When referring to fish being taxonomically identified to species level, were they identified in the field and returned to the environment or where they sacrificed and taken for identification under lab conditions (preserved entire sample in formalin)? Please specify in this sentence. Also, under DNA barcoding methods of collection, it is important to photograph each sample and upload this photograph on the BOLD database with it its barcode index number (BIN number), was this done? If so, please include this information. Furthermore, why specifically fin clips and not a muscle tissue sample? According to Weight et al. (2012) (DNA barcoding fishes, a good reference to include here), even though fin clips work, the most effective material is a small muscle biopsy from the specimen taken from the right side of the body. Please explain in detail why a fin clip over muscle tissue was done. Lastly, if fin clips were taken, was there evidence of the fish surviving if returned to its habitat and not preserved? Were there any infections caused which may have made the individual more susceptible to predation?

L118: Change to read as follows: “DNA extraction, polymerase chain reaction (PCR), purification and sequencing”. Combine your original sub-headings of “DNA extraction” and “PCR products and sequencing of PCR products” into one paragraph.

L119-L120: For the salting out method, a reference, Martinez et al. (1998) is provided, however, this citation refers to the salting out method from fish whole blood. Ideally, it would be better to provide a reference that directly links this method for extracting DNA from fin clips of fish as was stipulated, for example Taggart et al. (1992) or Sunnucks and Hales (1996) which in most cases for other references, related to this method, modified it from Miller et al. (1988).

L120-L126: Delete. Not necessary to go into detail if you provide the correct reference for the salting out method and protocol in which you followed.

L132-L135: The primers indicated in the manuscript are not the same primers used as Ward et al. (2005). They used FishF1, FishF2, FishR1, FishR2 and the primers included in the manuscript were coxla_fw, coxla_rv. Hence, please do not indicate that they were “adopted” from Ward et al. (2005) but rather provide a reference that used the same primers as was indicated in this manuscript. Change sentence to read as follows: “A partial fragment of (include the number of base pairs here) of the mitochondrial cytochrome c oxidase subunit 1 gene (COI) was amplified by PCR using the primers …….”.

L138: Please include the amount of water used to complete the 20$\mu$l reaction volume and change “PCR profile” to “PCR thermal protocol”.

L139-L142: Please include the reference that you used this protocol from or modified it from.

L144-L146: Change sentence to read as follows: “Successful PCR products were purified using an Exonuclease I-Shrimp Alkaline Phosphate (ExoSAP-IT) method (USB, Cleveland, OH) and sequenced using BigDye V3.1 (add manufacturers name) terminator chemistry in the forward and reverse direction on an ABI PRISM 3730xl DNA analyser (add manufacturers name) following the manufacturers protocol”.

L148:L159: For the data analysis section, methodology processes are not clearly explained and a lot more work needs to be done here. For instance, under this section it needs to be mentioned that successful sequences for each species was deposited into the Barcode of Life Data (BOLD) System (Ratnasingham and Hebert, 2007; <https://www.boldsystems.org>) under the project name LKCOX (remove this sentence from results section under molecular barcoding, L179-180 and add it under data analysis). In addition, once deposited, what exactly was the role of the BOLD database? Were the sequences from this study used to compare against available reference database sequences to find the closest/nearest matching species which was displayed as a percentage probability (0-100%)? Also, for each species, what was the number of sequences submitted into BOLD for comparison (is this the N samples column in Table 1)? Next, were the publicly available sequences with a >98% similarity/match downloaded from the BOLD reference database (and how many) to include with your sequences in running the haplotype analysis. In the results section, L196-L197 includes the above information, move this sentence here to the data analysis section. Also, L204-L206 of the results section needs to be moved here to the data analysis section.

How did you run the haplotype (phylogenetic) analysis? How did you get the relative number of haplotypes for each species? Did you use the software DnaSP? Was the neighbour joining tree rooted or unrooted for both Figure 1 and Figure 2? Please provide a reference for the bootstrap method of 1000 replicates. How was average pairwise haplotype distances calculated? Did you use ARLEQUIN software? Please stipulate what version of the BioEdit software was used and indicate what the total length of base pairs was used or trimmed to by the software.

Considering relevant species were sampled from four sites (mainly the Sea of Galilee), why was a haplotype network not included to show if some species indeed differed according to site based on their haplotype groupings?

**Results**

Results are very interesting! Figures and tables are clear and readable, however Figure 2 is not of great quality and more information needs to be incorporated in to the map (Fig 2A; see below corrections for further recommendations). Table 2 legend should be above the table and not below it. I would recommend for Figure 1 to exclude the K2P distances combined here and provide them as separate tables as has been done for Table 2, just separate it according to family using sub-headings. Also correct acronyms for Figure 2 and Table 2 (see below corrections for further recommendations).

Corrections:

L169-L170: Delete.

L171: Are there 25 fish species reported in the Lake? From the in text references reported for this sentence, Goren and Ortal (1999) reported 19 native species in the Lake; Ostrovsky et al. (2014) reported 19 native species in the lake; and I did not come across the number of native species for Ostrovsky et al. (2014) fisheries management chapter. Even Borovski et al. (2018) reports 19 native species and 8 exotic species, which gives a total of 27 and not 25. Please clarify.

L172-L176: Please provide the original author/identifier in brackets for all species mentioned in these lines. Change “schemes” to “sites”.

L177: At the end of the sentence add Table 1 in brackets.

L177-L179: Rephrase this sentence to read as follows: “Additionally, 20 sequences were analysed from two fish species, *H. molitrix* (19 sequences) and *O. aureus* (1 sequence), that were suspected as hybrids with aquaculture fish (hereafter referred to as ‘suspected hybrids’) (Table 1)”.

L179-L180: Delete and move to data analysis section under materials and methods.

L181-L182: As previously mentioned, can the authors make clear how many specimens were collected for each species, and out of this sample size, how many were successfully sequenced and add this to Table 1 as some sequences may have been unsuccessful. Also, at the end of this sentence add Table 1 in brackets.

L183-L184: At the end of the sentence add Table 1 in brackets.

L186: Add the “number” of species where new barcodes were added to existing barcodes in BOLD.

L188-L190: Change Table 1 legend to include sites and sample sizes for each species.

L196-L197: Delete and move to data analysis section under materials and methods.

L197-L199: Specify the exact number of species where molecular identification agreed with the original morphological identification.

L199-L201: Delete and move to data analysis section under materials and methods.

L204-L206: Mention how many species were in disagreement. Please rephrase the sentence to read as follows: “Three species were found to have disagreements between morphological and molecular barcoding identification. Further investigation was carried using all available COI barcode records from BOLD of query and related species”.

L206: Cannot start a sentence with the genus name of a scientific name abbreviated, need to write it out in full, “*Acanthobrama*”. Delete “we”.

L211-L214: Remove from results section and add to discussion section. You are only stating the main points of results and should not discuss them in depth in this section.

L215-L217: Please rephrase the sentence to read as follows: “Three *Gambusia affinis* samples collected, all with the same haplotype (GaA), was identified by BOLD as *G. holbrooki* (100% fit), followed by *G. affinis* (99.8% fit).

L217-L220: Please delete the last part of this sentence and move to discussion section. Please rephrase sentence to read as follows: “Comparison to all available *G. holbrooki* and *G. affinis* records from BOLD indicated that GaA haplotype clusters together with *G. holbrooki* records”.

L222: Remove “identification module”.

L223-L224: Rephrase this sentence to read as follows: “This disagreement will be discussed further later under its own sub-section”.

L225-L226: Rephrase sentence to read as follows: “Molecular identification using BOLD for samples of fish suspected as hybrids provided genetic evidence to support hybridization.

L228: Change “into” to “in”.

L229-L231: Delete this sentence, starting from “This is consistent”.

L234-L235: Add a full stop after “in both directions” and delete the remainder of the sentence starting from “again”.

L236: Change “another” to “further”.

L239-L252: For genetic distance from barcoding data, I would start off with discussing the results from the neighbour-joining phylogenetic tree and incorporating the K2P genetic distances with this. In figure 1, for certain haplotypes there as asterisk next to them. In the Figure 1 legend, please indicate what these asterisks mean.

L260-L263: Please rephrase this sentence to read as follows: “Unexpected results were, however, found within the Cichlidae clade of the neighbour-joining phylogenetic tree. *Oreochromis* aureus (Haplotype OaA) clustered together with *Sarotherod galilaeus* (Haplotype SgA), a species belonging to a different genus, rather than with *O. niloticus* (Haplotype OaB) with which its genus is shared”.

L263-L267: Please rephrase this sentence to read as follows: “Support from K2P distances between *O. aureus* and *S. galilaueus* (0.0095) is smaller in comparison to distances between other cichlids of Lake Galilee belonging to different genera, and even smaller compared to cyprinids belonging to different genera (0.08-0.23) (Fig. 1)”.

L267-L268: Delete this sentence here and move it to discussion section.

L269: Change “*G. rufa”* to *“Garra rufa”*.

L270: delete “we find”.

L271-L273: Please rephrase this sentence to read as follows: “Identical K2P distances was, however, found between *Acanthobrama telavivensis* and *Mirogrex terraesanctae*, formerly known as *A. terraesanctae* and later moved to a separate genus”. Reference [36] has been removed here as in the result section you do not include in text references.

L273-L274: Please delete this sentence, starting from “This relatively” and move to the discussion section.

L277: Delete “As mentioned before”. Start sentence with “Comparison of Garra rufa haplotypes”.

L281-L282: Delete “Samples identified morphologically as”. Start sentence with “Garra rufa fish”.

L283: After Ein Te’o put “north of the Sea of Galilee” in brackets. For Figure 2A, can the authors please explicitly label Asi stream, Ein Afek and Ein Te’ o stream in text in the correct location on the map to make these locations clearer in relation to the haplotypes. Also, include labels for eastern and western watersheds of Israel on the map (Fig. 2A). Furthermore, can you give Haplotype GrD its own circle and colour and make GrA haplotype in the Sea of Galilee the same colour as in Ein Te’o (the colours on the map look different).

L284: Make “the” prior to Sea of Galilee samples capitalized.

L284-L287: Please rephrase this sentence to read as follows: “The Sea of Galilee samples had five fish from Haplotype GrA, two fish from Haplotype GrC and one fish from Haplotype GrD. All three fish from Ein Te’o shared Haplotype GrA and all three fish from Asi stream shared Haplotype GrB (Fig. 2A).

L289-L298: Please rephrase Figure 2 legend to read as follows: “Sampling sites and clustering of Garra spp. Samples based on COI haplotypes. Acronyms of populations are: GrA – *Garra rufa* from Haplotype A; GrB – *Garra rufa* from Haplotype B; GrC – *Garra rufa* from Haplotype C; GrD – *Garra rufa* from Haplotype D; Gg – *Garra ghorensis* from the southern basin of the Dead Sea; Gj – *Garra jordanica* from the northern basin of the Dead Sea; GnA – *Garra nana* from Haplotype A; GnB – *Garra nana* from Haplotype B. (A) Map showing the sampling sites in a regional context. Different *Garra spp.*  Haplotypes were denoted by differently coloured circles in each sampling site on the map of Israel, Jordan and Syria regions. (B) A dendrogram constructed for Haplotypes obtained from samples collected in this study alongside Haplotypes mined from BOLD (see Process ID’s in Table S1). Next to the edges, haplotype acronyms are given as indicated above. Numbers next to edges denote percent bootstrap support. Colours of Haplotype text match the colour of the circles on the map (A)”.

L300: Delete “To put into context the *Garra spp.* Sampled from Israel,”. Start sentence with “Sequences of 16 *Garra spp.* From”.

L301: Delete “a” before “geographic location”.

L302: Add “assigned” before “to them” and add “see” before “Table S1” in brackets. Add “neighbour-joining” before “phylogenetic tree (Fig 2B)”.

L305: After 0.0381 in brackets, add “; Table 2). After “were found in fish from” add “the Sea of Galilee and a northern location in Syria (Fig 2A). Delete “two isolated locations” and “GnA, the *G. nana* haplotype found in the Sea of Galilee was found also in samples from a northern location in Syria”.

L308-L311: Please rephrase this sentence to read as follows: “Israel *G. rufa* samples from the Sea of Galilee and Ein Te’o had Haplotypes GrA, GrC and GrD. These Israel *G. rufa* Haplotypes clustered with BOLD Haplotypes of *G. jordanica* (Haplotype Gj), fish sampled from streams in the northern basin of the Dead Sea in Jordan (Fig. 2B)”.

L311-L314: Please rephrase this sentence to read as follows: “Close by, yet separated, in the phylogeny, was *G. rufa* fish, Haplotype GrB, from Asi stream, suggesting again greater similarity to *G. jordanica*, but some genetic separation from the Sea of Galilee population”.

L318: Insert “GrAsia –“ before “Turkey, Iran and Iraq” in the brackets.

L318-L320: Delete this sentence starting from “Thus, the results indicate that” and move it to the discussion session.

L321-L327: Figure legend for Table 2 should go above the table and not below the table. Provide a reason as to why Gr North has an asterisk sign and the other locations do not. Change acronyms for populations to read as follows: “Gr North – *Garra rufa* from Sea of Galilee and Ein Te’o of northern Jordan valley; GrAsi – *Garra rufa* from Asi stream of Beit she’an valley; Gj – *Garra jordanica* from the northern basin of the Dead Sea; Gg – *Garra ghorensis* from the southern basin of the Dead Sea; Gr Asia – *Garra rufa* from Asian countries including Turkey, Iran and Iraq; GnA – *Garra nana* from Haplotype A; GnB – *Garra nana* from Haplotype B”.

L333-L337: Please rephrase this sentence to read as follows: “The Israeli *G. rufa* fish (Gr North) are somewhat genetically divergent from *G. rufa* fish from eastern and norther Asian countries (Gr North vs Gr Asia mean K2P distances = 0.0025 vs 0.0462; Table 2)”.

L340-L341: Delete “from the Northern Jordan valley” and replace it with “(Gr North)”.

L343: Insert “south Asi stream of” before “Beit she’an valley”. Delete “(Asi stream)” and replace it with “(Gr Asi)”.

L344-L345: Delete “in the Hula valley”.

L350: insert “(mean K2P of 0.0587) after “from Asia”.

L351: Rephrase to read as follows: “*G. rufa* (mean K2P of 0.014) (Table 2)”.

**Discussion**

The discussion section is almost a repetition of the authors results section. More evidence needs to be provided here to support your results. Elaborate by including more examples of relevant recent literature to strengthen your arguments. Results are interesting, however a lot more work needs to be done in this section. Also, it is important to include any limitations that were raised during this this study and should be highlighted and discussed here by providing ways in the future as to how it can be avoided or improved on. Also, there is no conclusion section, which is very important to drive your take home message for this study. For instance, in the conclusion section by adding a how this study could contribute to the conservation of Israel’s native species and identification of hybrid and non-native invasive species through DNA barcoding would be a good as well as how it will improve in the future from this perspective.

Furthermore, before discussing the *G. rufa* complex in the discussion, authors need to discuss the other disagreements that were found from your results, namely *A. lissneri* and *A. telavivensis*; *G. holbrooki* and *G. affinis*; hybrids; Cichlidae; and *Acanthobrama-Mirogrex*, in more detail by referring to or by providing recent, relevant and supportive literature. In the results section, I indicated which sentences need to be moved to the discussion section relative to the above sentence as they should not be present in the results section.

Corrections:

L361: Reference [25], i.e. Ward et al. (2005) does not directly refer to DNA barcoding as being an important part of BOLD, perhaps rather refer to reference [40].

L361-L362: Please rephrase this sentence to read as follows: “In a previous study conducted by [39], the cichlids of Israel were barcoded with success”.

L362-L363: Please rephrase this sentence to read as follows: “In this study, focus was given to the fish found in the Sea of Galilee and surrounding water bodies in the region, including species other than cichlids”.

L367: After “12 species”, add “mostly belonging to the Cyprinidae and Cichlidae,”.

L370: For references [24,40] did both studies focus on similar families as this study? Is this a fair comparison?

L375-L377: Please provide a reference for this sentence.

L389: Please indicate how recent this isolation occurred in by means of a time frame.
