# Supplementary material for: Establishing and using a genetic database for resolving identification of fish species in the Sea of Galilee, Israel
Source: PLoS One. 2022 May 19;17(5):e0267021. doi: 10.1371/journal.pone.0267021 (PMC9119452; doi:10.1371/journal.pone.0267021)
Supplement: Attachment — Submitted filename: Response to authors revision 1 from reviewer 2.docx [file pone.0267021.s004.docx]

Response to authors revision 1_reviewer 2

**Introduction**

Comment: L8: Change “derived pressure” to “derived pressures”.

Comment: After “Further human derived pressures on natural fish diversity has also been documented due to introduction and invasion of exotic fish species [10-13].” continue with the following sentence, “Conservation measures intended to mitigate the impact of human derived pressures have largely been slow and inadequate, and as a result, populations of many freshwater species have been declining rapidly [5, 6, 8, 15].”, then end the paragraph with “Assessment of freshwater biodiversity and population status for effective management is, therefore a priority, however, can be considered a great challenge [1, 6, 14, 15].”

Comment: Start “Due to its arid climatic conditions ……” as a new paragraph.

Comment: L19: Change “about” to “approximately”.

Comment: L22-L24: Please change sentence to read as follows: “The native cyprinid Kinneret bleak *Mirogrex terraesanctae* (Steinitz, 1952), which is endemic to the sea of Galilee, is probably the most abundant species in terms of both numbers and biomass [20].”

Comment: L24-L26: Please change sentence to read as follows: “The native cichlid, Galilee tilapia, St. peter’s fish or Mango tilapia *Sarotherodon galilaeus* (Linnaeus, 1758), which is widespread in central and western Africa [8], is also abundant in terms of numbers and biomass, based on fishery landings, and is considered an important species to local fisheries [25].

Comment: L26-L27: Delete the sentence “While *M. terraesanctae* is endemic, *S. galilaeus* is widespread in central and western Africa [8]”.

Comment: L27-L31: Please change sentence to read as follows: “Non-native species, which were unintentionally introduced, include the European eel *Anguilla anguilla* (Linnaeus, 1759) that hitchhiked with mullet fry caught in coastal estuaries and stocked in the Sea of Galilee [21], the green swordtail *Xiphophorus helleri* (Heckel, 1848) [22] and the Peacock bass *Cichla kelberi* (Kullander and Ferreira, 2006), probably released into the Sea of Galilee by hobbyists [23]”. Out of curiosity, why did you replace *X. helleri* (spelling is with one “I” at the end, not two “I’s”) with the Oreochromis hybrid? Is it because the Oreochromis hybrid is not an exotic species and this was your focus? Would still be good to include the Oreochromis hybrid example in this paragraph.

Comment: L33-L36: Please change sentence to read as follows: “Additionally, significant non-native species including the common carp *Cyprinus carpio* Linnaeus 1758, the silver carp *Hypophthalmichthys molitrix* (Valenciennes, 1844), the thinlip grey mullet *Chelon ramada* (Risso, 1827) and the flathead grey mullet *Mugil cephalus* Linnaeus, 1758 were introduced to enhance commercial fishing [16, 22].”

Comment: L45: rewrite as “[e.g. 26-28].

Comment: L59: Replace “Here” with “For this study”.

**Materials and methods**

Comment: L75: Please include the dosage used for anaesthesia.

Comment: L77: Please change “100% ethanol” to “99.9% ethanol”.

Comment: L77-L78: Please change sentence to read as follows: “In most cases, after identification and sampling, fish recovered from anaesthesia and were returned to the environment after a monitoring period of ……. (please include this)”.

Comment: L78-L81: Please change sentence to read as follows: “Some fish, however, were sacrificed, photographed and fixed in formalin to be deposited in the National Natural History Collections of the Hebrew University of Jerusalem, Israel with a museum number (Table 1), serving as voucher specimens (transferred to 99.9% ethanol)”.

Comment: L79: Why were only some fish photographed and not all fish collected for fin clips? To my understanding, for DNA barcoding protocols, all individuals that were fin clipped should have photographs to be uploaded to the BOLD database. Also, why for so many of your species collected were no individuals taken as voucher specimens/deposited into the National collection? Is it because there are already these species present in the National Collection? I would expect for field sampling records in a study, at least 2 individuals of each species should be accessioned into the Natural collection. This allows other researchers to compare across years and study sites where a particular species is distributed.

Comments: Table 1:

- Please rephrase table heading to, “Fish species collected, barcoded and deposited at the National Natural History Collections of the Hebrew University of Jerusalem (NNHH-HUJ) by species and location”.
- Can you add the columns “Order” and “Family” in Table 1 to allow for the same format in Table 2?
- Can asterisks be added to the hybrids and a cross to A. *lissneri* as done in Table 2.
- Relabel “N samples barcoded” to “N successfully barcoded samples”
- Incorrect numbers of successful barcodes are reported in table 1 for *A. flaviijosephi* (9 instead of 8 for Beit She’an), *G. rufa* (7 instead of 8 for Sea of Galilee), *H. molitrix*, *H. nobilis*, and *S. galilaeus* when compared to the numbers of samples present in the LKCOX project on the BOLD database. For *S. galilaeus*, in the table you recorded 17 from the Sea of Galilee when in the BOLD database there are 14 records from this site; and for Ein Afek you recorded 4 in the table when there are 2 for this site. Also, on the BOLD database there are other sites including Ginosar Research (4), Naman River (2), and Volta Lake (4), could you have added some of these samples by mistake to the Sea of Galilee and Ein Afek samples?
- Double check the number of 176 published records is correct, in relation to above statement, and explain the difference between this number and 217 published records.
- *Garra jordanica* is not included in the table but is present in the LKCOX project on the BOLD database
- To my understanding, when depositing vouchers into a National collection, each individual gets a museum accession number. Why is there only one accession number for *A. telavivensis* when three specimens were deposited? The same for *G. rufa*, *A. flaviijosephi*, *C. zillii*, *S. galilaeus* and *O. aureus*.
- IS *Cyprinus carpio carpio* a sub-species of *Cyprinus carpio*? According to Eschmeyers Catalog of Fishes, there is no *Cyprinus carpio carpio* listed. Is this meant to be *Cyprinus carpio?*

**Results**

Comment: Double check the number of CO1 sequences (176) from the 22 freshwater species, does not add up to the 217 published records on the BOLD database.

Comment: Table 2: Change “N barcoded samples” to “N successfully barcoded samples”. Check the numbers reported under this heading are correct and correlate to the BOLD database published records.

Comment: Figure 2: Can you make GrA a darker purple. Also, can you make GrD a different colour like pink or yellow, currently the blue colour used is too similar to GrC.

**Discussion**

Thank you for including some of the limitations to your study as well as a conclusion section. This section brings the findings concisely together.

Comment: Please check that 176 CO1 sequences is correct from the 22 freshwater fish species.

Comment: Please rephrase the following to: “Thus, minor frequency new barcodes were identified for a high proportion of the native species, an indication for the genetic divergence and uniqueness of these regional native populations, except for a few discrepancies which will be discussed later on”.

Comment: Please rephrase the following sentence to: “For 19 out of the 22 species, morphological and DNA barcoding agreed on the species definition”.
