# Supplementary material for: Establishing and using a genetic database for resolving identification of fish species in the Sea of Galilee, Israel
Source: PLoS One. 2022 May 19;17(5):e0267021. doi: 10.1371/journal.pone.0267021 (PMC9119452; doi:10.1371/journal.pone.0267021)
Supplement: Attachment — Submitted filename: Response to reviewers_r2_20220330.docx [file pone.0267021.s006.docx]

**Response to reviewer’s comments:**

Firstly, we thank again for the constructive comments on the revised version. We addressed all comments, as detailed below, and submitted a revised version integrating all these modifications. Reading over the revised version, indeed reads better and therefore, we believe it is ready to be accepted.

**Reviewer #1**

Responding to comments made in-text.

Comment: In the abstract the word ‘probably’ was highlighted in the sentence: “Notably, the cyprinid fish defined as *Garra rufa*, should be considered as *Garra jordanica*.”.

Response: There was no comment attached, however, we decided to omit the word ‘probably’ from the revised text.

Comment: 8% is very high! (Referring to the sentence – “A genetic distance of 0.08 was found between barcodes of *Acanthobrama lisnneri/Acanthobrama telavivensis* and *Mirogrex terraesanctae*, which was a distance relatively small for between-genera and similar to the within-genera distance found between *Garra rufa* and *Garra nana* (Heckel, 1843).” )

Response: The sentence was revised to: “A genetic distance of 0.08 was found between barcodes of *Acanthobrama lisnneri/Acanthobrama telavivensis* and *Mirogrex terraesanctae*, similar to the within-genus distance found between *Garra rufa* and *Garra nana* (Heckel, 1843).

Comment: I don't like the term 'molecular taxonomy' because it refers to a field that do not exist! It's better to use molecular identification or something like this... (Referring to the first sentence in the discussion.)

Response: The term ‘molecular taxonomy’ was changed to ‘molecular identification’. Also, we reviewed the text, found another instance of ‘molecular taxonomy’ in the conclusions section and made a similar change.

Comment: What do you mean with 'anciente origin'? Fishes were introduced? Do you have a reference here? (Referring to the sentence “Cichlids in the Sea of Galilee are of ancient African origin and they constitute a key group in the lake, with both ecological and commercial significance.”).

Response: As the Sea of Galilee is younger than its native species, it is likely that all of them migrated (not introduced) and inhabited the lake. The original meaning in the text was to state that the cichlids were not introduced to the lake, but are native to it and from an African origin. However, to reduce misunderstandings the word ‘ancient’ was removed from the sentence. Additionally, a reference was added (Goren and Ortal, 1999).

Comment: Please provide a reference to “since pure O. niloticus are considered too cold-temperature sensitive to become established there.”

Response: A reference was provided in text (Zengeya et al., 2013)

**Reviewer #2**

**Introduction**

Comment: L8: Change “derived pressure” to “derived pressures”.

Response: Text was revised as suggested.

Comment: After “Further human derived pressures on natural fish diversity has also been documented due to introduction and invasion of exotic fish species [10-13].” continue with the following sentence, “Conservation measures intended to mitigate the impact of human derived pressures have largely been slow and inadequate, and as a result, populations of many freshwater species have been declining rapidly [5, 6, 8, 15].”, then end the paragraph with “Assessment of freshwater biodiversity and population status for effective management is, therefore a priority, however, can be considered a great challenge [1, 6, 14, 15].”

Response: Text was revised as suggested.

Comment: Start “Due to its arid climatic conditions ……” as a new paragraph.

Response: Text was revised as suggested.

Comment: L19: Change “about” to “approximately”.

Response: Text was revised as suggested.

Comment: L22-L24: Please change sentence to read as follows: “The native cyprinid Kinneret bleak *Mirogrex terraesanctae* (Steinitz, 1952), which is endemic to the sea of Galilee, is probably the most abundant species in terms of both numbers and biomass [20].”

Response: Text was revised as suggested.

Comment: L24-L26: Please change sentence to read as follows: “The native cichlid, Galilee tilapia, St. peter’s fish or Mango tilapia *Sarotherodon galilaeus* (Linnaeus, 1758), which is widespread in central and western Africa [8], is also abundant in terms of numbers and biomass, based on fishery landings, and is considered an important species to local fisheries [25].

Response: Text was revised as suggested.

Comment: L26-L27: Delete the sentence “While *M. terraesanctae* is endemic, *S. galilaeus* is widespread in central and western Africa [8]”.

Response: Text was revised as suggested.

Comment: L27-L31: Please change sentence to read as follows: “Non-native species, which were unintentionally introduced, include the European eel *Anguilla anguilla* (Linnaeus, 1759) that hitchhiked with mullet fry caught in coastal estuaries and stocked in the Sea of Galilee [21], the green swordtail *Xiphophorus helleri* (Heckel, 1848) [22] and the Peacock bass *Cichla kelberi* (Kullander and Ferreira, 2006), probably released into the Sea of Galilee by hobbyists [23]”. Out of curiosity, why did you replace *X. helleri* (spelling is with one “I” at the end, not two “I’s”) with the Oreochromis hybrid? Is it because the Oreochromis hybrid is not an exotic species and this was your focus? Would still be good to include the Oreochromis hybrid example in this paragraph.

Response: Text was revised as suggested. The hybrid tilapia was added back to the text with the appropriate reference to support it.

Comment: L33-L36: Please change sentence to read as follows: “Additionally, significant non-native species including the common carp *Cyprinus carpio* Linnaeus 1758, the silver carp *Hypophthalmichthys molitrix* (Valenciennes, 1844), the thinlip grey mullet *Chelon ramada* (Risso, 1827) and the flathead grey mullet *Mugil cephalus* Linnaeus, 1758 were introduced to enhance commercial fishing [16, 22].”

Response: Text was revised as suggested.

Comment: L45: rewrite as “[e.g. 26-28].

Response: Text was revised as suggested.

Comment: L59: Replace “Here” with “For this study”.

Response: Text was revised as suggested.

**Materials and methods**

Comment: L75: Please include the dosage used for anaesthesia.

Response: The anaesthesia dosage was added to the text.

Comment: L77: Please change “100% ethanol” to “99.9% ethanol”.

Response: Text was revised as suggested.

Comment: L77-L78: Please change sentence to read as follows: “In most cases, after identification and sampling, fish recovered from anaesthesia and were returned to the environment after a monitoring period of ……. (please include this)”.

Response: Text was revised as suggested. The monitoring period for the recovery of fish was added.

Comment: L78-L81: Please change sentence to read as follows: “Some fish, however, were sacrificed, photographed and fixed in formalin to be deposited in the National Natural History Collections of the Hebrew University of Jerusalem, Israel with a museum number (Table 1), serving as voucher specimens (transferred to 99.9% ethanol)”.

Response: Text was revised as suggested.

Comment: L79: Why were only some fish photographed and not all fish collected for fin clips? To my understanding, for DNA barcoding protocols, all individuals that were fin clipped should have photographs to be uploaded to the BOLD database. Also, why for so many of your species collected were no individuals taken as voucher specimens/deposited into the National collection? Is it because there are already these species present in the National Collection? I would expect for field sampling records in a study, at least 2 individuals of each species should be accessioned into the Natural collection. This allows other researchers to compare across years and study sites where a particular species is distributed.

Response: Specimens of all sampled species are already present (including collection voucher types) in the NNHC-HUJ. Furthermore, most of the fish were sampled and returned to the environment, including fish that had novel DNA barcodes. Only fish that were not returned were pictured, sampled for DNA extraction and then fixed in formalin and deposited in the collection.

Ideally, it would be best if for all barcodes in BOLD there would also be voucher specimens deposited in collections for possible future comparative studies. However, restriction on sampling of natural resources and practical considerations, since barcoding is done on very large numbers of specimens, make this ideal situation currently not feasible.

Comments: Table 1:

- Please rephrase table heading to, “Fish species collected, barcoded and deposited at the National Natural History Collections of the Hebrew University of Jerusalem (NNHH-HUJ) by species and location”.

Response: Text was revised as suggested.

- Can you add the columns “Order” and “Family” in Table 1 to allow for the same format in Table 2?

Response: These columns were added as suggested.

- Can asterisks be added to the hybrids and a cross to A. *lissneri* as done in Table 2.

Response: Text was revised as suggested.

- Relabel “N samples barcoded” to “N successfully barcoded samples”

Response: Text was revised as suggested.

- Incorrect numbers of successful barcodes are reported in table 1 for *A. flaviijosephi* (9 instead of 8 for Beit She’an).

Response: We thank the reviewer for thoroughly going over the data and identifying this mismatch. This number was corrected to 9 both in table 1 and in table 2.

- *G. rufa* (7 instead of 8 for Sea of Galilee).

Response: This was not a mistake, there are 8 G. rufa samples from the Sea of Galilee, one was accidently stored as G. jordanica. This was fixed in the database as per the comment below.

- *H. molitrix*, *H. nobilis*, and *S. galilaeus* when compared to the numbers of samples present in the LKCOX project on the BOLD database.

Response: Some of the samples that were deposited to BOLD were not analysed in this study since they were either belonging to specimen sampled from aquaculture farms (13 H. molitrix, 7 H. nobilis and 13 H.molitrix x H.nobilis hybrids) or they were not regional (4 S. galilaeus samples from Volta Lake in Ghana). Thus, LKCOX BOLD project contains more samples than reported in the manuscript. However, if one would like to know which are reported in the manuscript and which are not, the sampling location will immediately tell which samples are not from the locations reported in the manuscript.

- For *S. galilaeus*, in the table you recorded 17 from the Sea of Galilee when in the BOLD database there are 14 records from this site; and for Ein Afek you recorded 4 in the table when there are 2 for this site. Also, on the BOLD database there are other sites including Ginosar Research (4), Naman River (2), and Volta Lake (4), could you have added some of these samples by mistake to the Sea of Galilee and Ein Afek samples?

Response: There are 13 S. galilaeus records from the Sea of Galilee and 4 additional records from a sub-location belonging to the Sea of Galilee (Ginosar Research). For Ein Afek (part of the Naaman River system) there were also two sub-locations (Ein Afek and Naaman River). We prefer to keep the sub – location in the BOLD database, but coordinates were provided so people can see that it’s actually the same ecosystem.

- Double check the number of 176 published records is correct, in relation to above statement, and explain the difference between this number and 217 published records.

Response: After comparing the records deposited in BOLD with the reported data in tables 1 and 2, we noticed three samples of *Carasobarbus canis* (cc001-cc003) which were deposited to BOLD but were suspected as contaminants and therefore were not used for the analysis in the paper. To reduce further misunderstandings or possible mistakes, their sequences were removed from the BOLD database.

Without these three C. canis records and without the 37 records detailed in the above comments, after fixing the *A. flaviijosephi* numbers, the total number of sequences stands on 177 and not 176. This was corrected throughout the text.

We prefer to keep the sub – location in the BOLD database, but coordinates were provided so people can see that it’s actually the same ecosystem.

- *Garra jordanica* is not included in the table but is present in the LKCOX project on the BOLD database

Response: This record was mistakenly stored as G. jordanica, it was fixed in the database to have the same identification like the rest of the G. rufa from Israel.

- To my understanding, when depositing vouchers into a National collection, each individual gets a museum accession number. Why is there only one accession number for *A. telavivensis* when three specimens were deposited? The same for *G. rufa*, *A. flaviijosephi*, *C. zillii*, *S. galilaeus* and *O. aureus*.

Response: In the NNHC-HUJ, each collection event gets an accession number. Meaning that if fish of the same species were collected in the same time and place they all get the same accession number. Sometimes these are sub-divided into HUJXXXXX-01, HUJXXXXX-02 etc. For that reason, there is only one accession number for multiple fish of the same species.

- IS *Cyprinus carpio carpio* a sub-species of *Cyprinus carpio*? According to Eschmeyers Catalog of Fishes, there is no *Cyprinus carpio carpio* listed. Is this meant to be *Cyprinus carpio?*

Response: This was meant to be *Cyprinus carpio*, therefore *Cyprinus carpio carpio* was changed here and in table 2.

**Results**

Comment: Double check the number of CO1 sequences (176) from the 22 freshwater species, does not add up to the 217 published records on the BOLD database.

Response: We double checked the numbers as detailed above (in response to comments on table 1), and corrected this number to 177.

Comment: Table 2: Change “N barcoded samples” to “N successfully barcoded samples”. Check the numbers reported under this heading are correct and correlate to the BOLD database published records.

Response: The text was revised as suggested.

Comment: Figure 2: Can you make GrA a darker purple. Also, can you make GrD a different colour like pink or yellow, currently the blue colour used is too similar to GrC.

Response: GrA, GrC and GrD are haplotypes found for the same species in the same ecosystem (The Sea of Galilee). To make this point, we decided to assign all three of them with different shades of the same color (blue), in contrast to different haplotypes of the same or related species that were labelled by different colors. Therefore, we prefer to leave it in the same color scheme. The figure is also compatible with color blind readers since the haplotype designations are also provided in the figure.

**Discussion**

Thank you for including some of the limitations to your study as well as a conclusion section. This section brings the findings concisely together.

Comment: Please check that 176 CO1 sequences is correct from the 22 freshwater fish species.

Response: We double checked the numbers as detailed above (in response to comments on table 1), and corrected this number to 177.

Comment: Please rephrase the following to: “Thus, minor frequency new barcodes were identified for a high proportion of the native species, an indication for the genetic divergence and uniqueness of these regional native populations, except for a few discrepancies which will be discussed later on”.

Response: We did not change the text as suggested. This sentence describes the uniqueness of the native populations in the sea of galilee. The next sentence starts with the few discrepancies found between the morphological and the molecular identification and does not belong with the previous sentence.

Comment: Please rephrase the following sentence to: “For 19 out of the 22 species, morphological and DNA barcoding agreed on the species definition”.

Response: We changed the order of this sentence to start with the point that in most cases the morphological and the molecular identification agreed so it now reads as follows: “For 19 out of the 22 species, morphological and DNA barcoding agreed on the species definition, except for a few discrepancies which will be discussed later on”.
